# Helical polyamines†

**DOI:** 10.1039/d4sc05129g

**Published:** 2024-09-09

**Authors:** Daniël Hagedoorn, Sandra Michel-Souzy, Bartłomiej Gostyński, Hubert Gojzewski, Piotr Paneth, Jeroen J. L. M. Cornelissen, Frederik R. Wurm

**Affiliations:** a Department of Molecules and Materials, Sustainable Polymer Chemistry (SPC), MESA+ Institute for Nanotechnology, Faculty of Science and Technology, University of Twente P. O. Box 217 7500 AE Enschede The Netherlands frederik.wurm@utwente.nl; b Department of Molecules and Materials, Biomolecular Nanotechnology (BNT), MESA+ Institute for Nanotechnology, Faculty of Science and Technology, University of Twente P. O. Box 217 7500 AE Enschede The Netherlands; c International Center of Research on Innovative Biobased Materials (ICRI-BioM)—International Research Agenda, Lodz University of Technology Zeromskiego 116 90-924 Lodz Poland

## Abstract

Polymer microstructures rely on tacticity, yet exploration in polyamines has focused predominantly on atactic polymers. We introduce a method to synthesize a diverse library of *ortho* and *para*-cyanobenzenesulfonyl-activated-methyl aziridines using *R*, *S*, and racemic alaninol. Living anionic ring-opening polymerization of racemic sulfonyl aziridines yields soluble polymers, while enantiomerically-pure sulfonyl aziridines follow a dispersion polymerization with complete monomer conversion giving access to stereoblock copolymers. Removal of activation groups is achieved using dodecanethiol and *tert*-butylimino-tri(pyrrolidino)phosphorane to obtain isotactic or atactic linear polypropylene imines (LPPIs). High-purity L-PPIs are obtained in salt and neutral forms with high yields. Stereoblock copolymers of poly-*R-block-S*-polysulfonamides and respective polypropylene imine stereoblocks are synthesized, revealing helical structures in water influenced by the monomer type and sequence in CD spectroscopy. Molecular dynamics simulations confirm the helical nature of isotactic LPPIs in water. Bulk characterization demonstrates the first crystalline isotactic polyamines *via* spherulite growth in polarized light, atomic force microscopy and XRD analyses. In cell-transfection studies, the synthesized isotactic LPPIs exhibit lower toxicity and transfection efficiency than commercial hyperbranched polyethylene imine, with longer chains showing increased transfection efficiency. These isotactic polymers open avenues for complex macromolecular architectures with optically active polyamines akin to poly(amino acid)s but lacking hydrolytically cleavable amide links.

## Abstract 2

The first report on isotactic poly(propylene imine) and its stereoblock copolymers prepared by living anionic polymerization.

## Graphical abstract

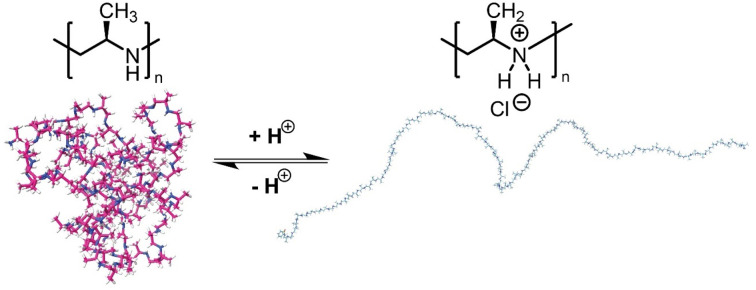

## Introduction

Tacticity is one of the fundamental properties of polymeric materials.^1^ Tacticity brings about supramolecular organization, which adds new properties to existing materials.^2^ This drives applications such as chiral-catalysis, separation and sensing.^3–5^

**Fig. 1:**
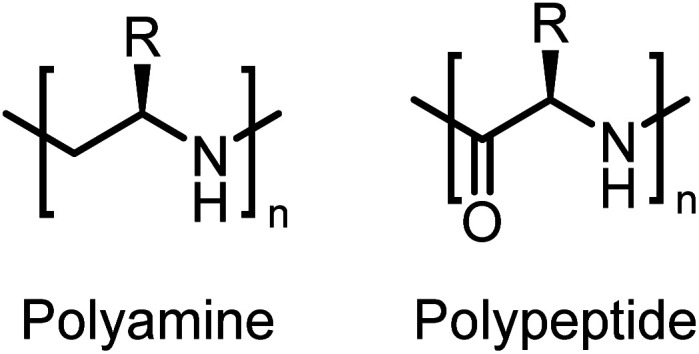
Chemical structures of polyamines and polypeptides.

Here, we present the first enantiomerically pure polypropylene imines and their stereoblock copolymers, *i.e.* isotactic main-chain polyamines, prepared from activated aziridines. Such polymers resemble a polyamine-substitute of polypeptides without the labile peptide links in the main chain (Fig. 1). Chiral oligoamines have been reported before from the reduction of polypeptides, multistep coupling or oxazolines,^6–10^ however no polymers or stereo-block copolymers have been reported to our knowledge.

Linear, mostly atactic, polyamines are typically prepared by the cationic ring-opening polymerization (CROP) of oxazolines.^11–14^ Attempts to polymerize aziridines had been already reported in the 1970s.^13^ However, for the resulting substituted polyamides no further investigation towards removal of the activating groups or potential applications was conducted and the field of activated aziridines was not further pursued at that time. In 2005, Bergman and Toste reported the unprecedented living anionic polymerization of tosyl-activated aziridines to insoluble polysulfonamides, but did not continue,^15^ thus, till today CROP of oxazolines is the leading road towards linear polyamines.^16^ Over the last few years, our group expanded the initial strategy on the living anionic polymerization (LAP) of activated aziridines, studied (co)polymerization kinetics, and developed different activating groups that were removed to obtain linear polyamines.^17,18^ However, when tosyl groups were used as activation groups, harsh conditions of desulfonylation led to partial chain scission making the preparation of well-defined and more sophisticated macromolecular architectures impossible.^17^ More recently, electron-withdrawing sulfonamide activating groups that could be removed mildly, thus preventing any chain scission, were introduced by us and Rupar's group, independently.^18,19^

Here, we present the first isotactic, *i.e.* enantiomerically pure, polypropylene imines prepared by LAP, using cyanosulfonyl-activated aziridines. As LAP prevents side reactions, perfectly linear polypropylene imines with high control over molar mass and microstructures are accessible.^18,19^ We also show the first stereoblock copolymers, *i.e.* poly-*R-b*-poly-*S*-propylene imine and *vice versa* and study their bulk properties and supramolecular organization in water and correlate these findings with molecular simulations. In addition, visualization of the secondary structures with AFM was conducted as has been shown before for other isotactic polymers, revealing that isotactic or stereoblock copolymers are semicrystalline materials.^20,21^

We start from amino alcohols (*i.e.* biobased alaninol) and using a two-step one-pot procedure activated aziridines were synthesized. In this optimized procedure, first, the activation with the sulfonyl group takes place and then ring-closure occurs. The monomers were polymerized using different monomer:initiator ratios (with degrees of polymerization of 60 to 300) and the sulfonyl activating groups were removed under mild conditions to obtain a systematic library of isotactic polyamines with different stereochemistry. The polyamines were investigated using various analytical techniques to study their molecular structure, organization, and other physical properties (NMR, GPC, TGA, DSC, optical and atomic force microscopies, XRD and CD). In particular, CD spectroscopy and AFM revealed a complex secondary structure of the isotactic polyamines, both block and homopolymer. Furthermore, DNA transfection was conducted using isotactic and atactic polymers and compared to hyperbranched polyethylenimine (hbPEI).

We propose a versatile synthetic method for producing isotactic polyamines with moderate to high yields. Enantiomerically pure polyamines serve as versatile building blocks for advanced macromolecular architectures *via* living or controlled polymerizations, giving access to block copolymers with various commodity monomers. Resilient against hydrolysis or enzymatic degradation, these chiral structures offer durable and partially biobased alternatives, akin to polypeptides.

## Results and discussion

### Monomer synthesis

To allow anionic ring-opening polymerization (AROP), the aziridine ring needs to be activated towards nucleophilic attack, which is usually achieved by electron-withdrawing sulfonamide groups.^15^ They stabilize the evolving aza-anion after ring-opening at the growing chain end by electron delocalization. Propagation then continues *via* such sulfonamide anions. Benzenesulfonamide-aziridines equipped with electron-withdrawing groups are the most promising monomers as the mild desulfonylation gives access to polyamines.^16,18,19^ There was one recent attempt using the *tert*-butyloxycarbonyl protecting group (BOC) as an activation group,^22^ however, only oligomers with relatively high molar mass dispersity were obtained.

**Scheme 1:**
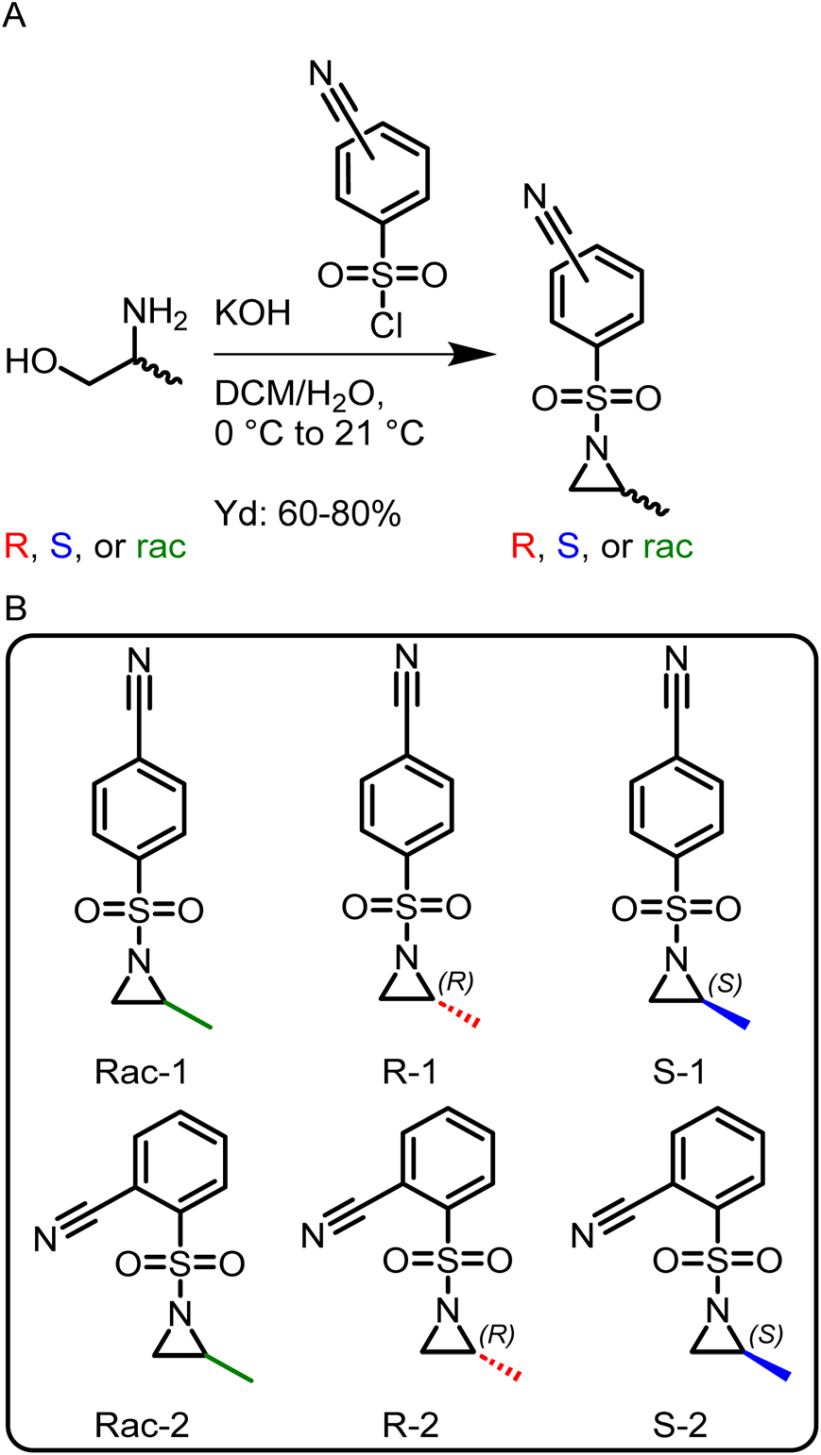
(A) Synthesis of activated aziridines in a one-pot, two-step procedure starting from racemic and enantiopure alaninol. (B) Overview of the monomer library prepared in this study.

Here, a library of activated aziridines was prepared from enantiomerically pure or racemic alaninol. To investigate the influence on solubility and desulfonylation, both *ortho*- and *para*-cyanobenzenesulfonyl chloride were used as the activating group (Scheme 1A). As Rupar *et al.* reported a higher solubility of *ortho*-nitrobenzenesulfonamide polymers but low stability of the respective monomer, we expected a similar trend for the less activated cyano-derivatives.^19^ Instead, however, the solubility of the *ortho*-substituted polymers was even lower compared to the *para*-derivatives, and no difference in the stabilities of the monomers was observed under ambient conditions over the course of at least 6 months by ^1^H NMR spectroscopy (Fig. S22 and S23†). Cyanobenzenesulfonyl chloride was used as it can be removed under mild conditions.^18,23^ To synthesize the activated aziridines, alaninol was treated with three equivalents of cyanobenzenesulfonyl chloride, to first activate the nitrogen and then transform the alcohol group into a leaving group for the ring-closure to the activated aziridine (see Scheme S2† for reaction details). A similar method for tosyl-substituted aziridines had been reported by Bieber *et al.*^24^ and was here adapted for the more electrophilic cyano-derivatives. The monomers (Scheme 1B) were obtained as off-white solids with yields up to 80%. The retention of the chirality was investigated with chiral HPLC. For the *ortho*-substituted monomers, there was a clear separation observed (Fig. S13 (rac-2), S17 (*R*-2), S21 (*S*-2)†). For the *para*-substituted monomers, no separation was observed on our setup under the used conditions.

### Polymerization and desulfonylation

**Scheme 2:**
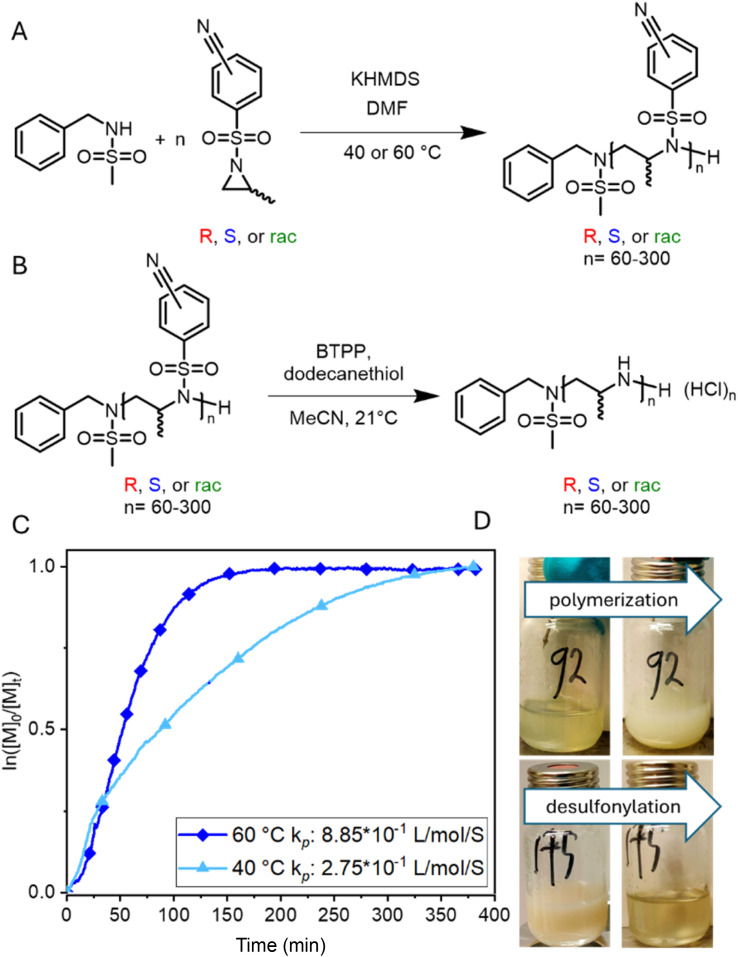
Synthesis of polysulfonamides and polymerization kinetics of activated aziridines. (A) polymerization or enantiomerically pure or racemic alaninol-based aziridines; (B) desulfonylation of polysulfonamides towards polyamines; (C) real-time ^1^H NMR kinetics of the anionic polymerization of enantiomer *S*-1 (conducted at 40 °C and 60 °C in DMF-*d*_7_ with *N*-benzyl methane sulfonamide as the initiator directly inside the NMR tube), and (D) photos of the dispersion polymerization of *S*-1 (top) and the desulfonylation reaction (changing from the dispersion to a solution).

**Scheme 3:**
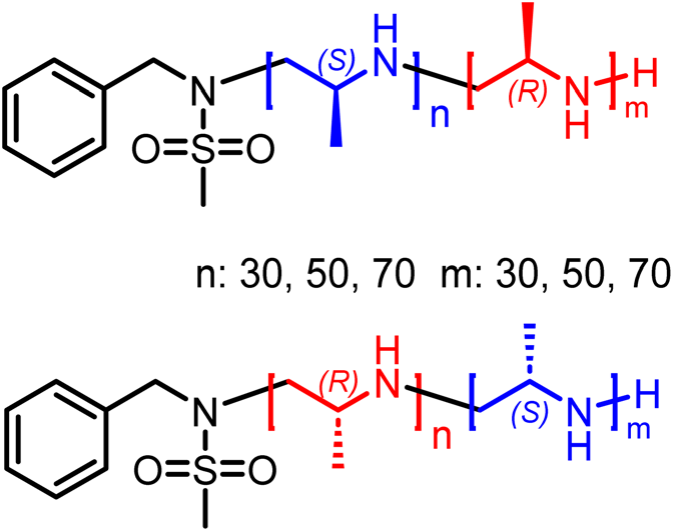
Overview of stereo-block copolymers.

The living anionic ring-opening polymerization of both racemic and enantiomerically pure activated aziridines was conducted at either 40 or 60 °C in DMF or acetonitrile with *N*-benzylmethansulfonamide as the initiator and potassium bis(trimethylsilyl)amide (KHMDS) as the respective base. We targeted different degrees of polymerization (DP), ranging from 60 to 300 repeating units (Table S1†). Despite the low probability of water acting as an unwanted initiator during the anionic polymerization of activated aziridines, as reported earlier by our group,^25^ we chose to use a pre-dried monomer and initiator for the higher DPs by azeotropic distillation from benzene at high vacuum for several hours before the polymerization (Scheme 2A). While the polymerization of racemic monomers occurred in solution, the polymerizations of the chiral monomers followed the characteristics of dispersion polymerization (Scheme 2D), producing a stable suspension several minutes after the addition of the initiator (Scheme S3†). Interestingly, ^1^H NMR spectra after 15 hours proved complete consumption of the monomers. The monomer conversion was also quantified by real-time ^1^H NMR kinetics (*S*-1 as the monomer, targeted DP of 100), indicating the living nature of the dispersion polymerization at 60 °C with a constant propagation rate (Scheme 2C and Fig. S25†). At 40 °C the propagation rate varied, which could be due to lower reactivity in the heterogeneous suspension particles and the reaction being conducted inside a NMR tube instead of a vial with constant stirring. However, full conversion was still observed suggesting that it could mean loss of mass control. An interesting observation was that the *k*_p_ of the dispersion polymerization at 60 °C kinetics was *ca.* 10 lower compared to the previously reported rate constant of the racemic monomer, which polymerizes in a homogeneous solution leading to presumably higher reaction rates.^26^ LAP and the complete monomer conversion also allow the synthesis of the first, aziridine-based stereoblock copolymers by sequential addition of the *R*- and *S*-enantiomers (Scheme 3). We targeted stereoblocks with different ratios of *S*- and *R*-monomers and an overall degree of polymerization of 100 (Table S1†). After consumption of the first monomer (confirmed *via*^1^H NMR), the corresponding monomer with the opposite chirality was added to the living dispersion polymerization leading to, again, full monomer conversion. ^1^H and ^13^C NMR characterization of the racemic polymers were conducted in solution, while isotactic polysulfonamides were insoluble in all common organic solvents.

**Fig. 2:**
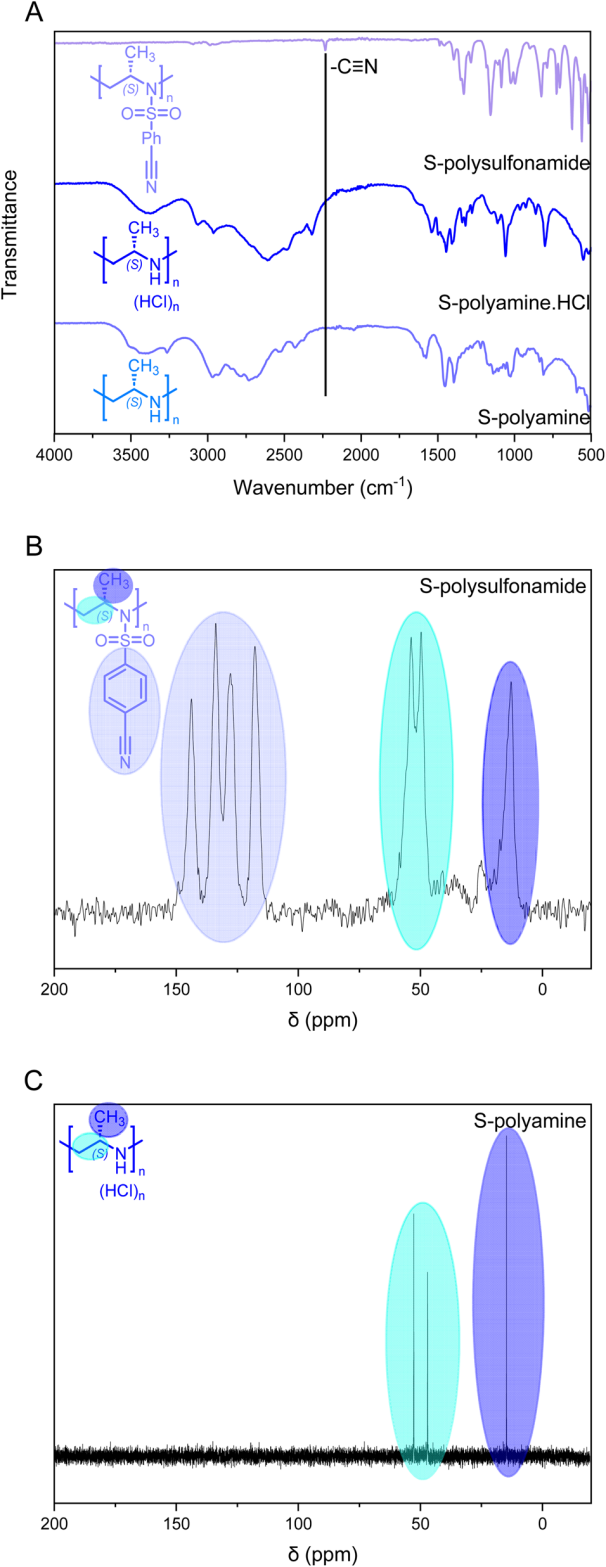
Evidence of the removal of the cyanobenzenesulfonyl group using a *S*-configured polymer with a DP of 150. (A) FTIR indicating the removal of the cyanobenzenesulfonyl group. (B) Solid-state ^13^C NMR spectrum of polysulfonamide (150 MHz) *vs.* (C) ^13^C solution NMR of polyamine (P13) (D_2_O, 100 MHz).

Solid-state ^13^C NMR of the isotactic polysulfonamides revealed the anticipated structure (Fig. 2C). The resonances of the activation group (*ca.* 150–120 ppm), the backbone (*ca.* 50 ppm), and the side chain (*ca.* 20 ppm) were clearly identified in all samples. Further identification by FTIR clearly indicated the nitrile group with absorption at 2230 cm^−1^ (Fig. 2A), which is in line with absorption charts. No difference in tacticity was observed for the polysulfonamides in FTIR.

Size exclusion chromatography (SEC) for the atactic polysulfonamides revealed a typical trace of a monomodal polymer as expected for a living polymerization with low molar mass dispersities between 1.09 and 1.14 (Fig. S26†).

The polysulfonamides were desulfonylated using an adapted procedure reported by Schmidt *et al.*^23^ and Gleede *et al.*^18^ (Scheme 2), which goes *via* a Meisenheimer intermediate to remove the activation group (Scheme S4†). We used the stronger non-nucleophilic phosphazene base *tert*-butylimino-tri(pyrrolidino)phosphorane (BTPP), compared to 1,8-diazabicyclo(5.4.0)undec-7-ene (DBU) as in the reported procedures, as it was mentioned by Schmidt *et al.* that the strength and solubility of the base are important parameters for the efficiency of desulfonylation.^23^ With BTPP, we found that the final polyamine was obtained with less impurities (as determined by ^1^H NMR after purification), compared to the samples treated with DBU (Fig. S29†). To purify the deprotected polyamine, the reaction mixture was treated with 2 M HCl(aq) to form the HCl salt of the polyamine. The impurities were then washed off with a 1 : 1 acetone : methanol mixture and the polymer was lyophilized to yield colourless, fluffy powders for the isotactic polymers and yellow solids for the atactic polymers (Table S3† for yields). The polyamines were stored as HCl salts because it improves shelf life as reported for PEI previously.^27^ We could isolate the free base of the polyamine by suspending it in DCM and adding excess triethylamine. The clear solution was evaporated and the solids were washed with acetone and lyophilized to yield off-white solids in all cases in yields up to 80%.

*Via* FTIR, the removal of the cyanobenzenesulfonyl group was observed; while the cyano-derivative exhibited a clear vibration at *ca.* 2230 cm^−1^ (Fig. 2A), the polyamine does not show this signal anymore and broad vibration signals for the resulting amine groups were observed. The desulfonylation was further confirmed by ^13^C NMR spectroscopy with the absence of the aromatic resonances (Fig. 2C and D). The solid-state ^13^C NMR spectrum of the precursor polymer clearly shows the resonances of the aromatic bonds for the cyanobenezensulfonyl group, while no aromatic resonances were detected in ^13^C NMR for the free polyamine in D_2_O; also ^1^H NMR underlines these findings in all cases. Furthermore, a clear difference in thermal stability was observed between the polysulfonamides and polyamines in TGA (Fig. S30†). The polyamines started to decompose at *ca.* 250 °C, while the polysulfonamides started at 350 °C.

**Fig. 3:**
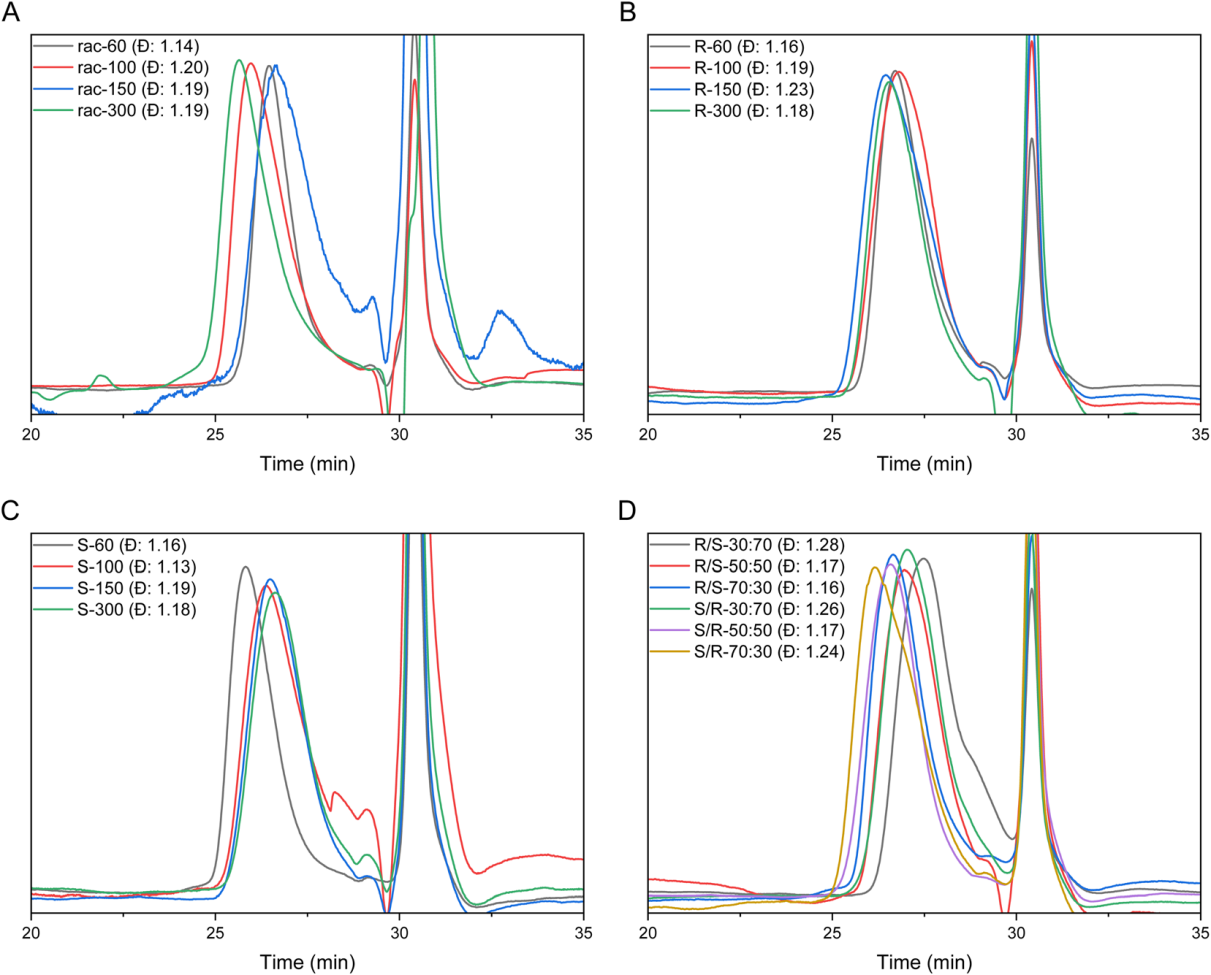
SEC elugrams of (A) atactic polyamines with DP ranging from 60 to 300 (P1–4). (B) Isotactic-*R*-polyamines with DP ranging from 60 to 300 (P5–8). (C) Isotactic-*S*-polyamines with DP ranging from 60 to 300 (P9–12). (D) Block polyamines in different ratios with DP of 100 (P13–18) (MiliQ with 0.1 M NaCl and 0.1% TFA, RI detection). The peak at 31 min is HCl.

All polyamine salts were characterized by SEC (Table 1 and Fig. 3) and ^1^H NMR (Table 1) under aqueous conditions. The SEC elugrams of all (co)polymers exhibit moderate to narrow monomodal molar mass dispersity. The molar masses of the polyamine salts were determined using end group analysis *via* the ^1^H NMR spectra (Table 1 and Fig. S32†).

**Table 1:** Molar mass and dispersity characterization of the polypropylene imines (as HCl salts) by NMR and SEC

P#	Polyamine·HCl (tacticity-DP)	Theoretical *M*_n_ (g mol^−1^)	*M*_n_ a (g mol^−1^)	*M*_n_ b (g mol^−1^)	*Đ* c
1	Rac-60	3600	4200	9300	1.14
2	Rac-100	5900	7400	11 000	1.20
3	Rac-150	8800	8800	8000	1.19
4	Rac-300	17 300	24 700	13 500	1.19
5	*R*-60	3600	3800	7800	1.16
6	*R*-100	5900	7200	7500	1.19
7	*R*-150	8800	9400	8300	1.23
8	*R*-300	17 300	16 300	8500	1.18
9	*S*-60^d^	3600	6200	12 800	1.16
10	*S*-100	5900	6500	10 300	1.13
11	*S*-150	8800	10 300	8800	1.19
12	*S*-300	17 300	21 700	8600	1.18
13	*R*-30/*S*-70	5900	6600	4600	1.28
14	*R*-50/*S*-50	5900	7600	6900	1.17
15	*R*-70/*S*-30	5900	7100	8300	1.16
16	*S*-30/*R*-70	5900	6300	6000	1.26
17	*S*-50/*R*-50	5900	6000	9100	1.17
18	*S*-70/*R*-30	5900	6800	9400	1.24

a Absolute number-average molar mass (in g mol^−1^) determined *via*^1^H NMR in D_2_O.

b Number-average molar mass (in g mol^−1^) determined *via* SEC in H_2_O (*vs.* pullulan standards).

c Dispersity determined *via* SEC in H_2_O.

d One-pot procedure, free base.

One-pot polymerization and desulfonylation were also conducted (Scheme S5†) using BTPP as the basic catalyst for the polymerization. The polymerization of the *S*-monomer was initiated with BTPP instead of KHMDS in acetonitrile with a targeted DP of 60 (P9). After confirmation of completion by ^1^H NMR extra BTPP and dodecanethiol were added to start the desulfonylation reaction. The resulting polyamine was directly washed free of HCl and the yield was 80% starting from the monomer with a dispersity of 1.16 which is comparable to the polymers synthesized in the two-step procedure as described in Scheme 2.

### Bulk and solution properties of polysulfonamides and polypropylene imines

#### Bulk properties

TGA of the polysulfonamides revealed a clear difference between the isotactic and atactic polymers. The atactic polymers started to decompose at *ca.* 349 °C, which is 10 to 30 °C earlier, depending on DP, compared to the isotactic counterparts (Fig. S27†). Interestingly, the atactic polysulfonamides revealed also a higher char yield of *ca.* 10% compared to the isotactic analogs (5%), indicating a slight influence of the supramolecular organization on thermal decomposition. After removal of the activation groups, the resulting PPI exhibited a thermal stability of up to *ca.* 250 °C, while also at *ca.* 100 °C some water was released, indicating the hygroscopic nature of the polyamines.

DSC revealed glass transition temperatures (*T*_g_) of *ca.* 64 °C at a heating rate of 10 °C min^−1^ for the polysulfonamides (Fig. S28†).^28^ While no melting peak was detected for atactic, amorphous polymers, as expected, also the isotactic polymers did not show any melting endotherm. We assume that the melting points are higher due to the scanning range of the DSC experiments or that the degradation temperature is reached before the isotactic polysulfonamides melt (*cf.* below for isotactic and atactic polyamines).

Tacticity can be studied through different techniques, such as FTIR and ^13^C NMR spectroscopy. While FTIR spectra for the sulfonylated samples did not show a difference in tacticity, the desulfonylated samples exhibited a difference between isotactic and atactic PPI (Fig. S33†).^29–32^ As observed by Stulz *et al.* the bands between 500 and 700 cm^−1^ allow for a clear distinction between isotactic and atactic polyleucine, for our polyamines a clear distinction was observed between 700 and 900 cm^−1^.^30^ Also ^13^C NMR spectra underlined the tacticity of the LPPIs. While in the atactic LPPIs, two resonances for every carbon atom were observed (Fig. S34A†), in the isotactic and stereoblocks only one resonance was observed (Fig. S34B–D†). In addition, the degree of protonation plays a prominent role in the folding. The difference between the ^1^H NMR spectra of the HCl salt and the free base (Fig. S35†) clearly points to a different structure. The HCl salt of polyamine has a clear peak separation and multiplicity, while the free base has broad peaks without splitting, suggesting that the former is better defined.

**Fig. 4:**
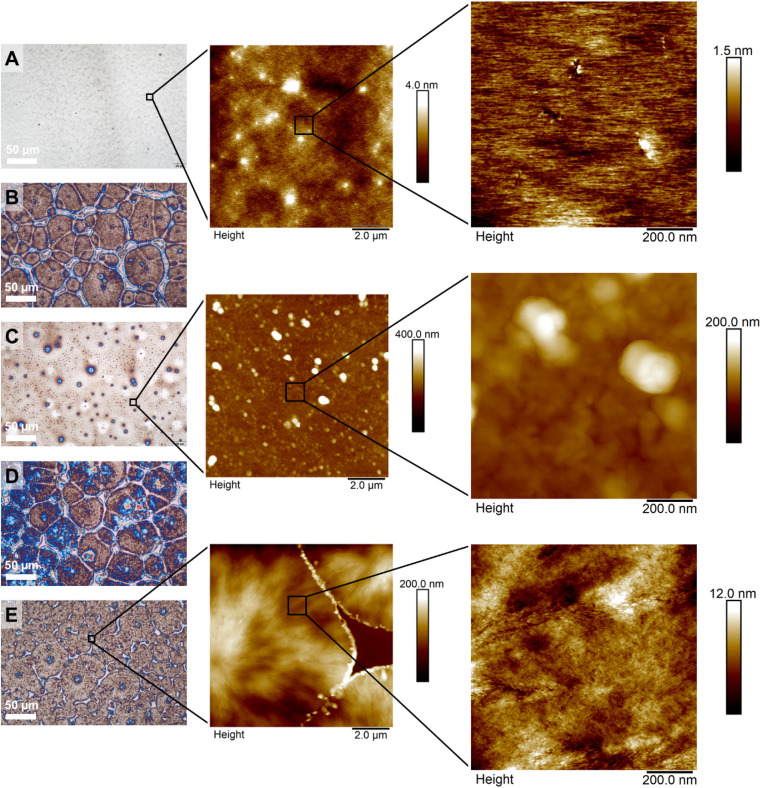
Optical microscopy (left column) and AFM height (middle and right columns) images of homopolyamines with a DP of 300 and block copolymers with a DP of 100. (A) Depicts a racemic polyamine·HCl (P4), (B) depicts a *R*-configured polyamine·HCl (P8), (C) depicts a *S*-configured polyamine·HCl (P12), (D) depicts a block copolymer (*R*-50/*S*-50, P14) and (E) depicts a block copolymer (*S*-50/*R*-50, P17). Black squares represent zoom-in images.

To visualize the crystallization of the PPIs and the surface structure, films were prepared by drop casting and investigated using optical microscopy and atomic force microscopy (AFM). We studied the polyamines, both atactic and isotactic (homo- and block copolymers, Fig. 4). For the atactic PPI (DP 300, P4), a homogeneous and continuous film was observed (Fig. 4A), indicating an amorphous polymer. For the isotactic PPIs (both *R* and *S* configurations) optical microscopy and AFM showed the formation of crystalline domains (developed spherulites in Fig. 4B and S34A;† limited crystallization, to small spherulites and organized structures between them in Fig. 4C). The stereoblock copolymers crystallized effectively towards spherulites, visible using optical microscopy (Fig. 4D and E) and AFM (Fig. S36B† and 4E). Besides microscopy, X-ray scattering data underlined the crystallinity of the isotactic homopolymers and the stereoblock copolymers (Fig. S37†).

#### Solution properties

**Fig. 5:**
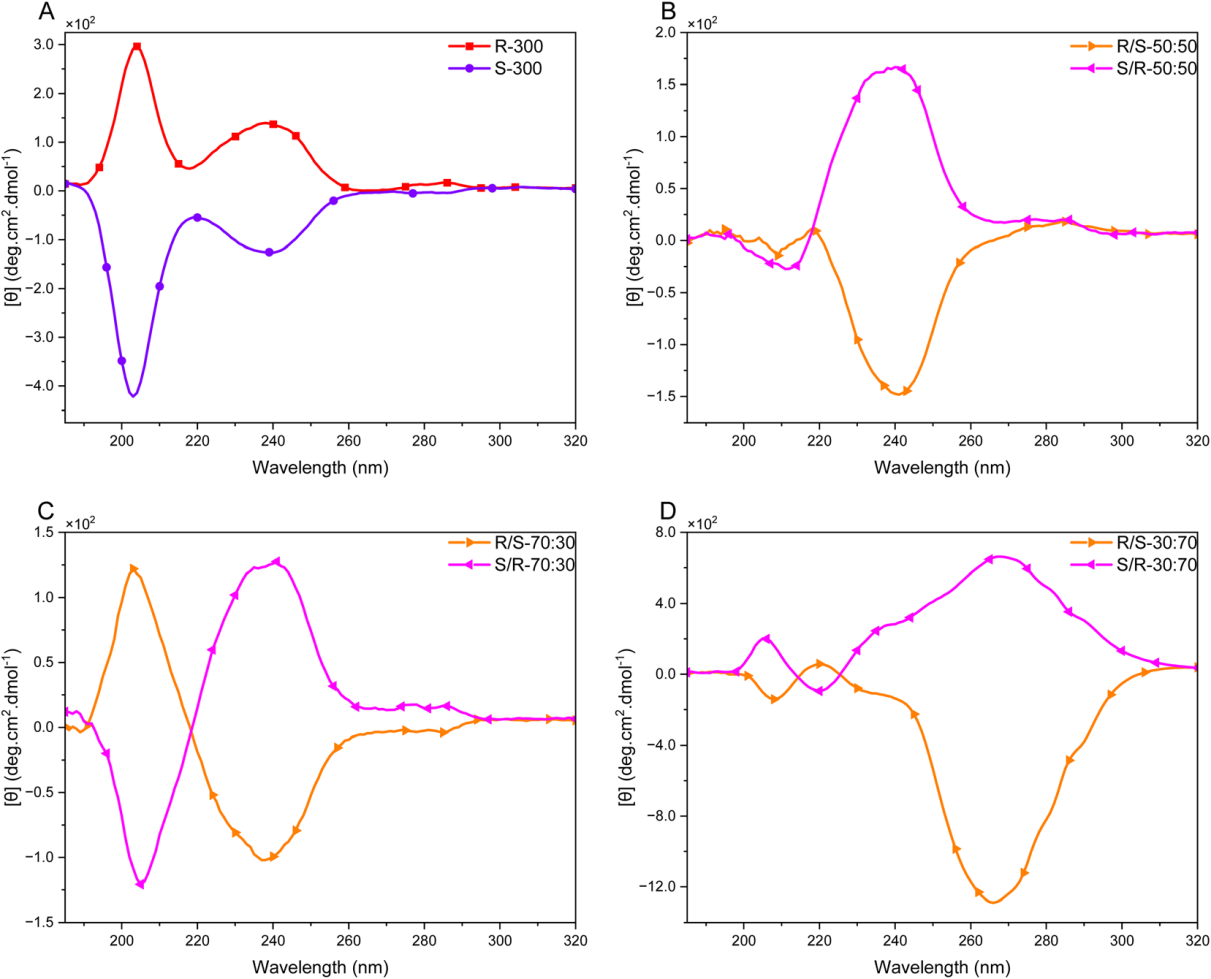
CD spectroscopy data of different PPIs, concentration: 5 mg mL^−1^ in demineralized water as HCl salt. (A) *R* (P8) and *S* (P12) homopolymer with DP of 300. (B) Stereoblock copolymer with equal parts (P14 & 17). (C) Stereoblock copolymer with 70/30 (P15 & 18). (D) Stereoblock copolymer with 30/70 (P13 & 16). All block copolymers are structured with initiator: (A) : (B). The *Y*-axis was calculated in mean residue ellipticity as described by Kelly *et al.*^35^

Isotactic polyamines might be utilized in a variety of applications, such as gene transfer, enantioselective separation and catalysis.^6,33,34^ In order to obtain further insight into the chiroptical properties of the polymers reported in this contribution, and their secondary (*i.e.* helical) structure and assembly, CD spectroscopy was used to study these isotactic polyamines and their corresponding stereoblock copolymers in aqueous medium. The CD spectra of the polyamine·HCl salts of isotactic polyamines with either *R* or *S* configuration exhibited two opposite CD signals confirming the formation of a chiral structure (Fig. 5A).

The first signal between *λ* = 200–210 nm can be attributed to the backbone of the polymer, *i.e.* the main chain chirality likely originating from an *n*_N_ → 3S transition (Rydberg excitation) in the asymmetric C–N repeat unit.^36^ In the UV/Vis spectra of the different polymers (Fig. S38†) the absorptions are masked, but in all relevant CD spectra, an almost symmetrical Cotton effect is observed. In the CD spectra of l-amino acids in water, a comparable Cotton effect is observed, with slightly blue shifted maxima and a similar molar ellipticity.^37,38^ In these publications, the Cotton effects are assigned to electronic transitions in the carbonyl group, which is evidently not present in the polyamines. For the polymers derived from *R*-alaninol the CD spectra displayed a positive signal and, for the *S*-based variants this signal was negative.

A second, distinctive, CD absorption was found between *λ* = 220–250 nm. We tentatively assign this signal to electronic transitions originating from the aromatic initiator group (*N*-benzylmethansulfonamide, which is not removed under the desulfonylation conditions). In the UV/Vis spectra, this absorption is clearly visible for all polymers studied, pointing to a substantially large extinction coefficient (Fig. S38†). The initiator moiety is, in this case, assumed to act as a probe for the local chiral environment, where no exciton coupling is observed – as is expected due to the relatively low concentration of initiator groups. Also, here the R-monomers give rise to a positive Cotton effect in this case with *θ*_240_ = +137 deg cm^2^ dmol^−1^ with a virtually mirror image signal for the S variant. Finally, a minimal CD effect was observed at *λ* > 260 nm, which we tentatively assign to the association of (parts of) the protonated polymer chain.^39^

Despite the polyelectrolyte character of the polyamine·HCl salts, we expect a similar dynamic secondary structure as found by Bloksma *et al.* for main-chain chiral poly(2-oxazoline)s.^40^ In order to confirm this analogy, we measured the CD spectra of P8 (*R*-300) at different temperatures and observed a virtually linear temperature dependence for the different CD maxima (Fig. S39A†). At the measured concentration the broadened Cotton effect between *λ* = 220–250 nm shows 2 separate maxima at *λ* = 230 and 246 nm, that both decrease in molar ellipticity with increasing temperature. On the other hand, the more intense signal attributed to the main chain chirality at *λ* = 208 increases in intensity combined with a significant redshift, which is in contrast with water soluble oxazolines that exhibit a blue shift with increasing intensity up to 60 °C before decreasing in intensity.^41^

We attribute the observed decrease assigned to the aromatic initiator group, to increased macromolecular dynamics perturbing the local chiral environment at elevated temperatures. The increased magnitude at *λ* = 205–215 nm (Fig. S39A†) accompanied by the concomitant redshift of the CD maximum, points to a more profound chiral orientation of the C–N *n*_N_ → 3S transition.^36^ This might be the result of an increasing persistence length of the charged polyamine.HCl salt with increasing temperature. Since no clear isodichroic point is observed, an obvious transition between (regions of) a folded and unfolded backbone conformation seems not to be present.

The conformation of polyelectrolytes is typically influenced by their concentration because at higher concentrations the charge screening is more pronounced. In the case of P8 (*R*-300) a clear increase in molar ellipticity is observed when stepwise going from a concentration of 10 mg mL^−1^ to 1 mg mL^−1^ (Fig. S40A†). The concentration dependence at *λ* = 195–205 nm is non-linear within the range studied and accompanied by a blueshift of the CD maxima, while in the *λ* = 230–246 range a much smaller concentration effect is observed. These data are, as expected, pointing to an increased persistence length (*i.e.* a better-defined chiral conformation) of the polyamine. The optically active local environment around the aromatic initiator groups is much less affected by the polyelectrolyte concentration.

The polymerization method reported here allows for the synthesis of stereoblock copolymers, *i.e.* polymer chains composed of a segment of one enantiomeric monomer and a segment of the opposite stereoisomer.^42^ The HCl salts of the stereoblock co-polyamines were studied with CD spectroscopy in demineralized water (Fig. 5B–D) as well as a selection of mixtures of the different macromolecules at 1 mg mL^−1^. The latter set of data can provide some initial insight into the polymer–polymer interactions and the possible formation of stereo complexes. In the case of the 50/50 stereoblocks (Fig. 5B) the equal amounts of stereoisomers in the polymer backbone cancel out the contribution in the *λ* = 200–210 nm range, while the initiator-based probe centered at *λ* = 240 nm results in a signal with similar shape and intensity as found for the homopolymers. The sign, however, of this Cotton effect seems to be determined by the second block as *θ*_240_ = +167 deg cm^2^ dmol^−1^ for P14 and *θ*_240_ = −147 deg cm^2^ dmol^−1^ for P17. The studied non-symmetric stereoblocks gave consistent CD data with the above observations. For P15 and P18 (Fig. 5C) the *θ*_240_ values are of comparable magnitude and the backbone signals are clearly present with about 40% intensity of the signals found for P8 and P12; well in line with the ee of a 70/30 ratio of enantiomers. The stereoblock copolymers with the second segment being the longest, *i.e.*P13 and P16, gave significantly different behavior (Fig. 5D). While the CD signals in the *λ* = 200–210 nm are in line with the expectations based on a 40% ee of the last enantiomer in a 30/70 ratio, a relatively large, irregularly shaped, observed Cotton effect is centered at *λ* = 265–270 nm. In these signals still a shoulder is present around *λ* = 240 nm for both studied stereoblock copolymers. Given the relatively low absorbance between *λ* = 260 and 300 nm (Fig. S39B†) and the intense Cotton effect measured in this region, a high chiral anisotropy for these samples is suggested by the data. To get more insight into the polyelectrolyte folding, CD spectra were measured at different temperatures (Fig. S39†). Also, for P13 an increase in CD intensities was observed; when monitored at *θ*_221_ and *θ*_268_ this is a virtually linear increase with increasing temperature, similar to P8. At *θ*_268_ the increase seems to level off, but with Δ*θ* = 250 deg cm^2^ dmol^−1^ the change is about one order of magnitude larger compared to the temperature dependence at the other wavelengths. Upon cooling the temperature effects appear to be reversible.

Based on the above chiroptical data, a model is suggested where a homochiral isotactic polyamine has a dynamic locally ordered structure. Probably due to intra- or intermolecular assembly a chiral environment is created around the polymer initiator. Under the studied conditions these polymers behave as polyelectrolytes, where the presence of charges influences the conformational behavior. As the polymers are isolated as their HCl salts and these are well soluble in demineralized water, in solution an equilibrium is present between protonated and unprotonated amine groups. This will allow for the above-mentioned association of chains, where extended parts line up. Elevated temperature or polymer concentration will result in a better-defined chain structure – as reflected by the increase in the *θ*_200–210_ regime, while under these conditions the local chiral environment surrounding the initiator is hardly affected by the changing parameters, which is reflected in minor changes of *θ*_240_ compared to other wavelength sections. This is also observed in the case of the stereoblock copolymers, where despite different ee in the different polymers the position and magnitude of *θ*_240_ in the CD spectra do not change. It should be noted that the sign of the Cotton effect originating from the initiator appears to be dictated by the stereochemistry of the second part of the polymers. This can be explained by a folding process or complex formation, where a chiral pocket is formed, but this is still not understood at the (macro)molecular level.

The formation of polymer (stereo)complexes is a function of many parameters. Apart from temperature, polymer and salt concentration, also the presence of other stereoisomers will influence the complexation. Therefore, under a selected set of conditions, a variety of mixed samples was studied by CD spectroscopy (Fig. S41†). It is striking that for all samples studied, hardly any CD effects are measured, except for some backbone contributions of sample mixtures with matching stereochemistry (*i.e.* Fig. S41C, E and F†). These observations suggest that indeed a subtle assembly takes place between polymers (segments) that have a matching stereochemistry. Under the conditions studied, this assembly appears to be disrupted by the addition of a non-matching chirality. The position of the initiator group in the stereoblock copolymers apparently also plays a role in this, as (i) its CD sign inverses in the polymers and (ii) when the second block is longer (*i.e.* 30/70) a pronounced chiral structure is formed that is not observed in any of the other polymers studied. This latter observation for P13 and P16 might point to a kind of helical superstructure, also since the temperature profile of P13 is non-linear – pointing to a more complex process than just more chain dynamics. It is, however, without a doubt that further experiments (*e.g.* combining spectroscopy with scattering studies and microscopy) are necessary to understand the complex solution behavior of the chiral polyelectrolytes in detail. These investigations are ongoing, but we feel that these are beyond the scope of the present contribution.

### Molecular dynamics

**Fig. 6:**
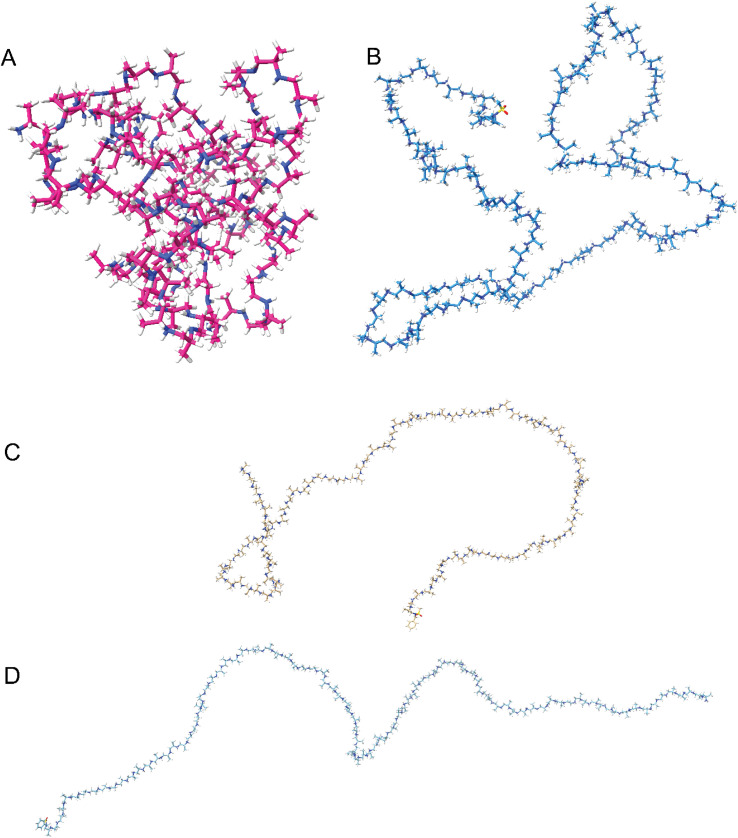
Snapshots of protonated and deprotonated PPIs by molecular dynamics. (A) Non-protonated (*i.e.* free base) *R*-100 (P6). (B) Re-protonated *R*-100 (P6). (C) Fully protonated rac-100 (P2). (D) Fully protonated *R*-100 (P6).

To further understand the secondary structure formation and what effect the protonation has, we used molecular dynamics (MD) to calculate the structure in both protonated and deprotonated forms at 300 K in an octahedral solvent box (TIP3P water model, 12 Å around the solute) by means of the GAFF2 forcefield as implemented in Amber22 software (details of the simulation procedure can be found in the ESI†). The results indicate that the non-protonated polymers tend to fold into a globule (Fig. 6A), independent of their tacticity. The globules are not observed with CD spectral data because the atactic polyamines give no signal. Globule formation continues up to 20% protonation of the total number of monomer units. For stereoblock copolymers, the main difference observed is chiral domain formation upon folding (Fig. S42A,† duplex). When the degree of protonation is larger than 20%, the intra-molecular electrostatic repulsion prevents them from adopting a globular shape and the polymers tend to stay in the elongated form. Re-protonation after folding gives back the elongated form (Fig. 6B), indicating a dynamic system. Duplex (Fig. S42B†) and triplex systems showed identical behavior giving rise to supramolecular assembly that can be undone through protonation. This behavior of protonated systems of multiple chains lining up we predicted through the CD data and is seen back with the duplex and triplex systems. No interaction is observed between the chains towards a supramolecular structure (Fig. S42B†).

In the elongated state (deprotonated) turning and twisting of the backbone is observed (Fig. 6B and D), indicating a helical structure. It is not as well-defined as observed with peptides, due to the more flexible backbone but this is also observed for polypropylene (PP).^43^ Additionally, when compared with ordered, chiral structures, the randomly structured protonated polyamines tend to exhibit structures that resemble random coils whereas the chiral systems retain their helical-like secondary structure (Fig. 6C and D). This ties in with the opted model based on the CD spectroscopy data, for the homochiral isotactic polyamine we suggested that there are local ordered structures which the calculations also propose (Fig. 6B and D).

### DNA transfection

Hyperbranched poly(ethylene imine) (hbPEI) was the second polymeric transfection agent discovered.^44^ Also, its linear counterpart has already successfully advanced to clinical trials.^45,46^ In the previous work, we investigated linear PPI for transfection of plasmid DNA into 293T cells.^18^ To date, no isotactic polyamines were investigated for DNA transfection, especially regarding potential supramolecular structures and how they influence DNA transfection efficiency. Only, some oligo- and polyamines either surfactant-like or as hollow particles have been used for DNA binding, and no transfection was attempted.^10,14,47^*S*-Configured polypropylene imine was used to investigate if chirality had an influence compared to racemic. The effect of polyplex formation seems to favor the *S*-configuration, therefore, the *R*-configuration was not investigated.^48^ Furthermore, longer chains were investigated because lengths should not influence cytotoxicity largely but have higher transfection efficiency.^45^ A nitrogen/phosphate ratio (N/P) of 5/1 was made because it is known for hbPEI to have low toxicity and good transfection efficiency.^49^ Furthermore, the N/P ratio of 20/1 was studied because it has been observed in the previous work that almost all DNA was combined with the polyamine starting at that ratio for PPI.^18^ Here the C2C12 mouse myoblast cell line from ECACC was used with plasmid DNA (pCMV-GFP from Plasmid Factory) with which a significantly better transfection was observed compared to the human kidney HEK293 cells (data not shown). A commercial lipid vector Lipofectamine™ 3000 was used as a positive control for the transfecting agent. Transfection experiments were carried out by incubating C2C12 cells (seeded at 15.000 cells per well, 96-well plate) with plasmid DNA encoding for green fluorescent protein (pCMV-GFP, 0.25 μg per well) for 6 h in Dulbeccos Modified Eagle Medium (DMEM), followed by an additional 40 h in DMEM^+^ to allow GFP expression.

**Fig. 7:**
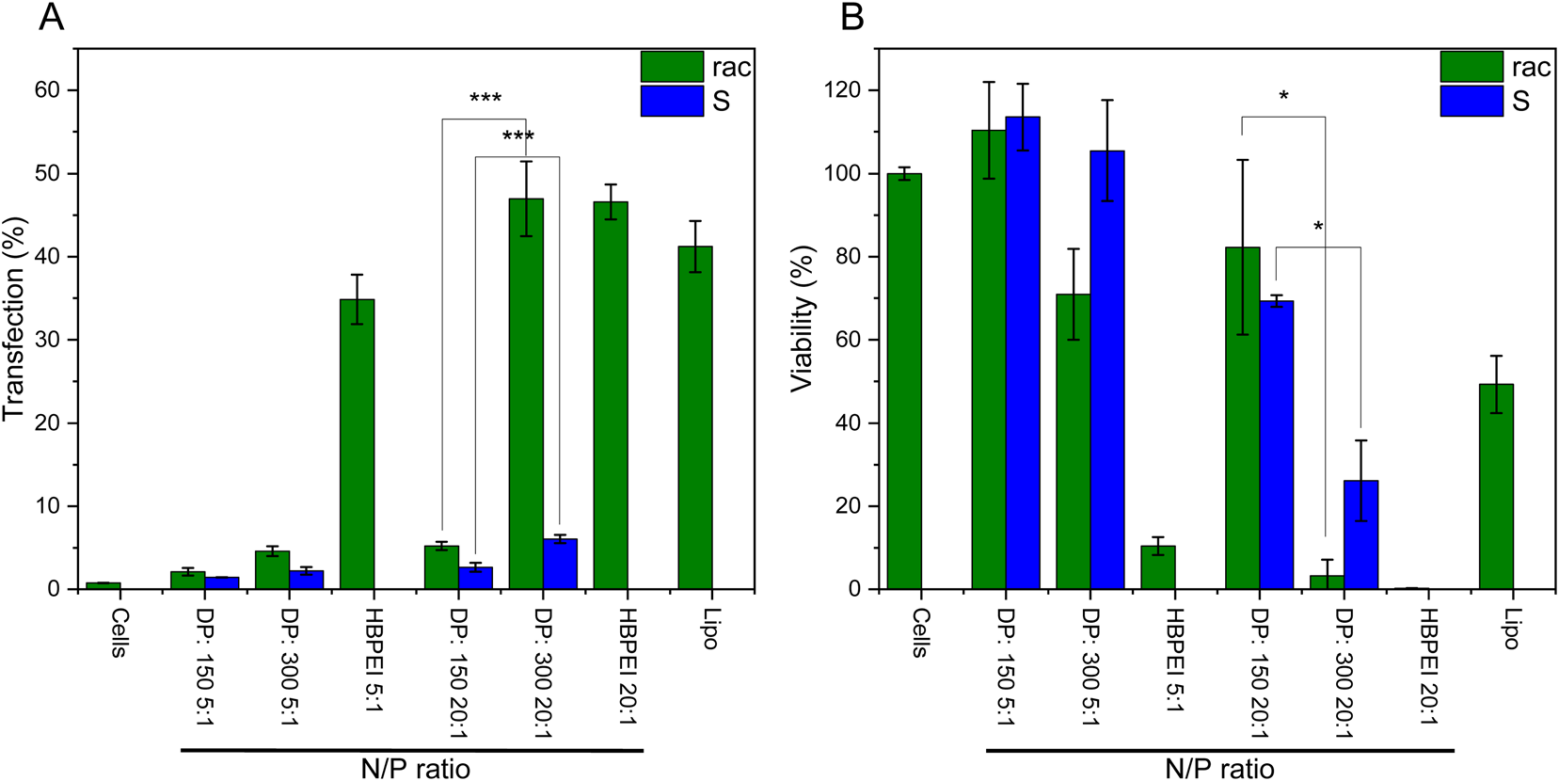
DNA transfection using PPIs: (A) GFP expression levels after transfection with the C2C12 cell line (Student's *t*-test: *P < 0.05; **P < 0.01; ***P < 0.001; error bars represent ± standard deviation). (B) Cell viability after 12 hours. N/P ratios of 5/1 and 20/1 were taken and compared to lipofectamine and commercial hbPEI (25 kDa).

It was observed that in all cases the atactic PPIs exhibited superior transfection efficiency compared to the isotactic PPIs (Fig. 7A). As reported for PEI a longer degree of polymerization increases the transfection efficiency capabilities, this can be observed for the 20/1 ratio between the 150 (P11), and 300 (P12) DP isotactic polyamines distinctively. The 300 DP has a three times higher transfection compared to the 150 DP. For the cell viability, the inverse is observed; the 300 DP isotactic PPI (P12) has a lower cytotoxicity compared to the atactic PPIs (Fig. 7B). Therefore, it is possible to further increase the N/P ratio which can balance out for the lower transfection capabilities. These results indicate a certain effect on the supramolecular assembly of PPIs in water and their efficiency in gene transfection, a future study will continue in understanding further characteristics, *e.g.* also in block copolymers and other macromolecular architectures on the gene transfection efficiency.

## Conclusion

This is the first report on isotactic poly(propylene imine) and its stereoblock copolymers prepared by living anionic polymerization. Living ring-opening anionic polymerization of cyanobenzenesulfonyl-activated aziridines was conducted and the activating groups were removed to obtain linear PPIs in excellent yields. These isotactic PPIs were crystalline as proven by microscopy (formation of spherulites) and scattering data while atactic PPI was amorphous.

CD spectroscopy in water, combined with molecular dynamics, proved the formation of higher order structures in solution that points to helical formation. The assemblies can be easily disrupted by adding a non-matching chirality, but no change in formation is observed upon heating or with different concentrations.

We also demonstrated the application of isotactic PPIs as a cell transfection agent. The transfection efficiency was not higher than that of established agents. But the cytotoxicity is lower, therefore, higher ratios can be applied to compensate for the lower transfection capabilities.

We envision that these new isotactic polyamines can be used in many applications to further broaden the polymer landscape with polyamines as well-defined building blocks accessible by anionic polymerization. For this reason, future work will present a broader library starting from bio-based amino acids to synthesize polymers and block copolymers by combination with other materials using living anionic polymerization.

## Author contributions

All authors have given approval to the final version of the manuscript. DH – carried out the synthetic work, analyses and writing of the paper; SMC – performed the biological assays and compiled and described the results; BG and PP – were responsible for the computational studies; JC – supervised the CD studies and edited the paper; FRW – developed the concept, supervised the synthetic part and edited the paper.

## Conflicts of interest

There are no conflicts to declare.

## Supplementary Material

SC-015-D4SC05129G-s001

## Data Availability

The data supporting this article have been included as part of the ESI.†
