# Supplementary material for: Helical polyamines
Source: Chem Sci. 2024 Sep 9;15(39):16377–90. doi: 10.1039/d4sc05129g (PMC11409658; doi:10.1039/d4sc05129g)
Supplement: SC-015-D4SC05129G-s001 [file SC-015-D4SC05129G-s001.pdf]

Supplementary Information (SI) for Chemical Science.
This journal is © The Royal Society of Chemistry 2024

Supporting Information

for

Helical Polyamines

*Daniël Hagedoorn[a], Sandra Michel - Souzy[b], Bartłomiej Gostyński[c], Hubert Gojzewski[a], Piotr Paneth[c], Jeroen J. L. M. Cornelissen[b], Frederik R. Wurm[a]**

[a.] Sustainable Polymer Chemistry (SPC), Department of Molecules and Materials, MESA+ Institute for Nanotechnology, Faculty of Science and Technology, University of Twente; P.O. Box 217, 7500 AE Enschede (The Netherlands)

[b.] Biomolecular Nanotechnology (BNT), Department of Molecules and Materials, MESA+ Institute for Nanotechnology, Faculty of Science and Technology, University of Twente; P.O. Box 217, 7500 AE Enschede (The Netherlands)

[c.] International Center of Research on Innovative Biobased Materials (ICRI-BioM)—International Research Agenda, Lodz University of Technology, Zeromskiego 116, 90-924 Lodz (Poland)

Contents

Contents 2

Materials 3

Methods and Characterization Techniques 3

Syntheses 6

- Synthesis of Initiator 6
- Synthesis of Activated Aziridines 6
- Monomer stability 23
- Anionic Polymerization of Activated Aziridines 24
- Desulfonylation of Polysulfonamides 30

Supramolecular Assembly 43

Molecular Dynamics 46

References 47

Materials

All solvents were purchased from Boom unless otherwise described in at least p.a. purity and used as received. DL-Alaninol (98%), *o*-cyanobenzesulfonyl chloride (97%), L-alaninol (95%) and sodium sulfate (99%) were purchased from ABCR. *N,N*-Dimethylformamide-d_7 (99.5%), (tert-Butylimino) tris(pyrrolidino) phosphane (97%), chloroform-*d* (99.8%), potassium hydroxide (85%), hydrogen peroxide (30%, stabilized), sulfuric acid (95-97%) and *hyperbranched*polyethlenimine (25 kDa, 99%) were purchased from Merck. D-Alaninol (98%) and 1-dodecanethiol (95%) were purchased from TCI. *p*-Cyanobenzensulfonyl chloride (97%) was purchased from VWR (Apollo Scientific). Potassium bis(trimethylsilyl)amide (98%) was purchased from Chemcruz. 1,8-Diazabicyclo[5.4.0]undec-7-ene (99%) was purchased from Fluka. Dry acetonitrile (99.7%) and dry *N,N*-Dimethylformamide (99.8%) were purchased from Thermo Fischer.

Methods and Characterization Techniques

Atomic Force Microscopy (AFM)

AFM was performed on a Bruker, MultiMode 8 with NanoScope V controller in the PeakForce Tapping mode at room temperature. During scanning, force−distance curves were collected following a sine-wave sample-tip trajectory with a frequency of 2 kHz and utilizing a peak-force amplitude value of 50-75 nm. Soft cantilevers with sharp tips were used, with a nominal spring constant of 0.4 N/m and a silicon-made tip with a nominal radius of 2 nm (Bruker, ScanAsyst-Air). The AFM data was further processed in the NanoScope Analysis software (ver. 3.00). The sample solutions had a concentration of 3 mg/mL in water and were drop coated onto Piranha-solution cleaned silicon wafers and left to dry in the air to form films.

Centrifuge

For the centrifuge the Hermle Z36HK is used. Centrifuging was done for 10 minutes at 8000 rpm using 50 mL plastic tubes from Vos instruments at room temperature.

Chiral High-Pressure Liquid Chromatography (cHPLC)

HPLC for chiral separation was performed on a Jasco HPLC (ADC: LC-NetII/ADC, pumps: PU-2086, autosampler: AS-2055, oven: CO-4060 and UV-detector: UV-2075). The analysis of the monomers was conducted using Amylose *tris* (3,5-dimethylphenylcarbamate)-immobilized on 5 µm silica gel column at 25 °C and 1 mL/min in hexane/EtOH 93/7. Measurements were plotted with OriginPro 2023b software from OriginLab Corporation.

Circular Dichroism (CD) Spectroscopy

CD measurements were performed using a Jasco J1500 spectropolarimeter with Peltierapparatus (Jasco PTC-517) in the range 180–340 nm. The spectra were collected with a scanning speed of 50 nm/min, CD scale of 20 mdeg/0.05 dOD, digital integration time (D.I.T.) of 1 sec and a bandwidth of 1.00 nm in demineralized water. Measurements are done in a 1-mm cell (Quartz glass high performance, QS) at room temperature, unless otherwise described. Measurements were plotted with OriginPro 2023b software from OriginLab Corporation.

Column Chromatography

Column chromatography was performed on a Buchi Pure C-850 FlashPrep. Flash mode was used with a 50 g column (self-poured silica gel 60 from Millipore). A gradient from 0-50% ethyl acetate in petroleum ether was used with a flow rate of 45 mL/min. The UV detection was on scan mode: 265-400 nm and the sensitivity on high.

Cytotoxicity Assay

Cytotoxicity assays were carried out in the same procedure as the gene transfection studies. In a typical procedure C2C12 or Hek293 cells were seeded at 10 x 10^3 in a 96-well plate along with 200 µL of Dulbecco's Modified Eagle's Medium (DMEM) supplemented with 10% FBS, 100 U/mL penicillin, 100 µg/mL of streptomycin and 2 mM of L-Glutamate ($DMEM^+$). Medium and supplements were purchased from SigmaAldrich and the cells were from ECACC, Cells were incubated until a confluency of 70-95% was obtained. The medium was aspirated from the cells and replaced with DMEM without FBS ($DMEM^-$) and polyplexes, then the cells were incubated at 37 °C and in 5% CO_2 and 95% humidity. Afterwards the transfection medium was aspirated and replaced by $DMEM^+$ and the cells continued incubating to allow GFP expression. Afterwards cell viability was assessed by alamarBlue™ cell viability assay. The cells were incubated with 44 µM of resazurin for 1 h followed by fluorescence measurement on a plate reader (excitation 570 nm and emission 585 nm). Untreated cells (i.e. cells that were not transfected) served as positive control and were assigned 100% viable. Experiments were done in triplicate.

Differential Scanning Calorimetry (DSC)

The TA DSC25 was used to determine thermal transitions. The samples (2 to 5 mg) were loaded into DSC pans, then quenched to -80 °C and subsequently heated to 240 °C at a rate of 10 °C/min. The melting points and enthalpy were determined under nitrogen atmosphere. Tzero sample press kit was used in combination with Tzero die set to clamp the Tzero lids with the Tzero pans, all from TA instruments. Measurements were plotted with OriginPro 2023b software from OriginLab Corporation.

Fourier-Transformed Infrared Spectroscopy (FT-IR)

Attenuated total reflection (ATR) spectroscopy was carried out on an Alpha P ATR spectrometer, which is equipped with a diamond crystal. The solid samples were pressed on the crystal. Measurements were plotted with OriginPro 2023b software from OriginLab Corporation.

GFP Gene Transfection Studies

Transfection studies and cytotoxicity assays were conducted in parallel. In a typical procedure cells were seeded at 50 x 10^3 cells per well in a 24-well plate along with 1 mL of cell culture medium supplemented with 10% FBS, 100 U/mL penicillin, 100 µg/mL of streptomycin and 2 mM of L-Glutamate ($DMEM^+$). Cells were incubated until a confluency of 70-95% was obtained. Prior to the transfection, medium was aspirated from the cells and replaced with cell culture medium without FBS ($DMEM^-$) mixed with different ratio of polyplexes. The cells were incubated overnight at 37 °C and in 5% CO_2 and 95% humidity. Afterwards the transfection medium was aspirated and replaced by $DMEM^+$ and the cells continued incubating for 48 h to allow GFP expression. The cells were then collected after trypsinization. Transfection efficiency was analyzed by flow cytometry using a MACSQuant® Analyzer 10 Flow Cytometer with an excitation of 488 nm and an emission of 525 nm.

Mass Spectrometer (MS)

Advion Expression L compact mass spectrometer was used for the MS measurement. MeOH was used as eluent with a flow rate of 0.3 mL/min in positive mode. The data was recorded with Advion CheMS Express version 6.9.41.2 and analysed with Advion Data Express version 6.9.41.2

Molecular Dynamics (MD)

Polymer Preparation

The polymers under investigation were considered as a chain of connected monomers referred to as "residua". In order to perform calculations, we generated appropriate MD residuum type (.prepi) files from the respective monomers using the following approach:

1. Initially, structures for all types of monomers were created and capped with methyl groups, i.e. we replaced one of the hydrogen atoms with methyl groups at locations where bonds between monomers would exist. These capping groups provided an approximation of the rest of the chain, aiding in the generation of more realistic partial charges.
2. The structures of all methyl-capped monomers were optimized with the semiempirical xTB method[1] using the ORCA 5.0.3 software.[2] Subsequently, for each optimized structure, a bundle of conformers was generated using the Crest software to obtain a diverse set of conformations for each compound.
3. Among the generated conformers, those with a root-mean-square deviation (RMSD) of less than 1 kcal/mol compared to the lowest-energy structure were discarded. The retained conformers were then re-optimized using the B3LYP-D3/def2-TZVP level of theory. All of these minimized structures were confirmed as local minima through vibrational analysis. Structures with energy difference less than 1 kcal/mol relative to the conformers with the lowest energy were discarded as being identical to them.
4. In the next step, molecular partial charges were generated using the Restrained Electrostatic Potential (RESP) method.[3] This process was carried out using the online RED Server.[4] The server was also used to generate .mol2 structure files to which the averaged partial charges were assigned. These files served as input for the .prepi file creation procedure which was performed using the Antechamber and Prepgen subprograms of the AmberTools22 package.[5]

Calculations

The actual simulations were carried out using the pmemd.cuda module of the Amber22 suite of codes[5] with GAFF2 force field.[6,7]

5. The solvent used was the TIP3P water model [8] and the systems in question were enclosed within truncated octahedral boxes, with a 12 Å buffer around the solute. In the case of the protonated amines, the charge of the system was neutralized using Cl- ions.
6. Calculations for periodic boundary conditions at 300 K consisted of the following steps:
    a) 2000 steps of energy minimization with restraints of 1500.0 kcal/mol imposed on the solute;

b) 2500 energy minimization steps without any restraints;
c) Heating: temperature increasing from 0 K to 300 K in 25000 steps of a length of 1 fs, with 10.0 kcal/mol restraints imposed on the solute;
d) Density equilibration: 25000 steps each of 2 fs. 8 Å cutoff radius for electrostatic interations;
e) Equilibration phase: 25000 steps at 2 fs. 8 Å cutoff radius for electrostatic interations;
f) Production phase: 5000000 steps at 2 fs. 8 Å cutoff radius for electrostatic interations.

Each of the resulted trajectories was subsequently clustered using k-means algorithm to obtain 10 clusters and 10 representative structures for a given trajectory.

Nuclear Magnetic Resonance (NMR) Spectroscopy
^{1}H-,^{13}C-NMR spectra were measured on a 400 MHz Bruker AVANCE III AMX system or 600 MHz Bruker AVANCE NEO system. The temperature was kept at 298 K during the measurements unless otherwise described. As deuterated solvents $CDCl_3$, D_2O or *N,N*-DMF-d_7 were used. MestReNova 14 from Mestrelab Research S.L. was used for analysis or with OriginPro 2023b software from OriginLab Corporation. The spectra were calibrated against the solvent signal ($CDCl_3$: H=7.26 ppm, D_2O: H=4.79 ppm, DMF-d_7: H=8.03, 2.92, 2.75 ppm).

Optical Microscope
Images were taken on an Olympus BX60 with 50x magnification. The samples were prepared as described under AFM. The pictures were acquired with Olympus CellSens Entry version 3.2.

Polarimeter
The specific rotation of the chiral monomers were measured with a Vernier (Chem-Pol) polarimeter with a 589 nm LED source and the data was collected with a LabQuest mini (Model 2) and analysed with Logger Pro version 3.16.2 from Vernier Science Education. The monomers were solubilized in acetonitrile for the measurements.

Polyplex Preparation
The polyamines were dissolved in sterile PBS buffer solution (pH = 7.4). In a typical procedure various amounts of stock solution, PBS buffer and plasmid DNA were combined in Eppendorf tubes to give N/P ratios of 5 and 20. The N/P ratio is the molar ratio between the nitrogen (N) atoms of the polyamine and the phosphate (P) atoms of the plasmid DNA. The polyplexes were incubated at RT for 15 min before use or measurements. The plasmid pCMV-GFP expressing the GFP was purchased from the Plasmid Factory.

Size Exclusion Chromatography (SEC)
Polysulfonamides: SEC measurements were performed in DMF (containing 1 g/L of LiBr) at 60 °C and a flow rate of 1 mL/min with a PSS SECcurity as an integrated instrument, including three PSS GRAM column (100/1000/1000 g/mol) and a refractive index (RI) detector. Calibration was carried out using polystyrene standards supplied by Polymer Standards Service. The SEC data were plotted with OriginPro 2023b software from OriginLab Corporation.
Polyamines: SEC measurements were performed in MilliQ (containing 0.1 M NaCl and 0.1% TFA) at 25 °C and a flow rate of 1 mL/min with a PSS SECcurity as an integrated instrument, including four PSS Novema Max columns (guard, 30 Å and 2x 1000 Å, all 5 µm) and a refractive index (RI) detector. Calibration was carried out using pullulan standards supplied by Polymer Standards Service. The SEC data were plotted with OriginPro 2023b software from OriginLab Corporation.

Thermogravimetric Analysis (TGA)
TGA was conducted on a TA Instruments TGA 550 system. The sample was heated from room temperature to 600 °C at a rate of 10 °C/min under a nitrogen flux of 40 mL/min. Aluminium sample pans, 80 µL with Stainless steel bails from TA instruments were used. Measurements were plotted with OriginPro 2023b software from OriginLab Corporation.

UV-Vis
Ultraviolet visible spectroscopy (UV–visible) was taken by Jasco V-630 UV–visible spectrometer (Jasco, Japan). Measurements were done in a 1 mm cell (Quartz glass high performance, QS) at room temperature. Measurements were plotted with OriginPro 2023b software from OriginLab Corporation.

X-Ray Diffraction (XRD)
Powder XRD measurements were performed on a Bruker D2 PHASER. The following measurement parameters were applied: a 2θ range of 10-70°, a step size of 0.05° 2θ, an integration time of 1.5 seconds, Cu Kα source at 10 mA and 30 kV. Measurements were plotted with OriginPro 2023b software from OriginLab Corporation.

Syntheses

Synthesis of Initiator

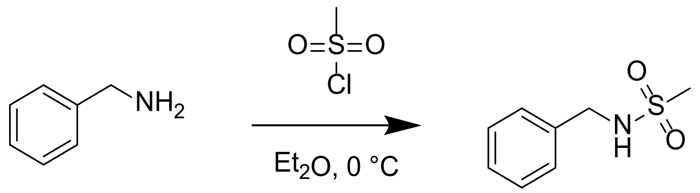

Scheme S1. Synthesis of *N*-benzylmethansulfonamide.

The compound was synthesized according to literature procedure.[8]

Synthesis of Activated Aziridines

An adapted literature procedure was taken for the synthesize.[9] A round bottom flask, equipped with magnetic stirrer, was charged with potassium hydroxide (93 mmol, 7 eq) in water (5 mL) and cooled in an ice-bath to 0 °C. Then the 2-aminopropan-1-ol (13 mmol, 1 eq) in DCM (5 mL) was added and the reaction mixture was purged with nitrogen. Finally, the ortho/para-cyano benzenesulfonyl chloride (40 mmol, 3.0 eq) in DCM (10 mL) was added dropwise (DCM should not boil). Initially, the reaction mixture appeared as a clear solution, which turned into a suspension over time. The reaction mixture was stirred for 1 hour at room temperature. Then, the mixture was partitioned between DCM and water and the layers were separated. The aqueous layer was extracted with DCM twice. The organic layers were combined, washed with water, dried over Na_2SO_4, filtered and concentrated at reduced pressure to afford a yellow oil that solidified to an off-white solid upon standing. The crude product was purified by column chromatography (wet loading, 24 g silica, gradient: 0-40% EtOAc in PE). The fractions containing the product were concentrated under reduced pressure to afford a cloudy oil, which solidified on standing to an off-white solid, yields typically above 60%.

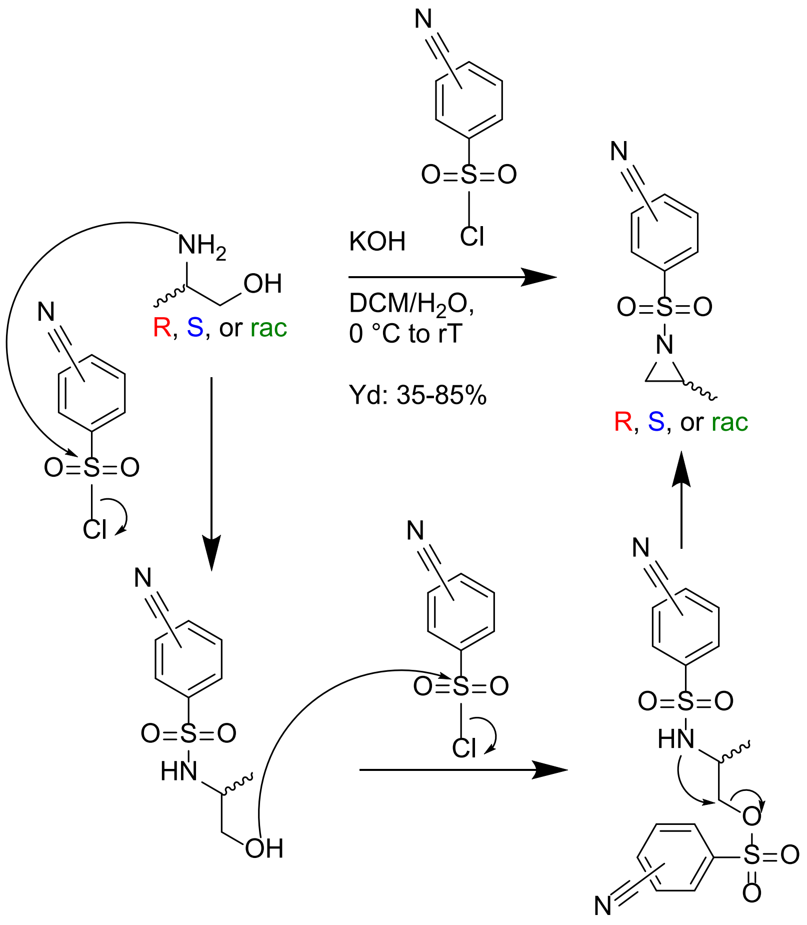

Scheme S2. Proposed reaction mechanism of the monomer synthesis.

4-((2-methylaziridin-1-yl)sulfonyl)benzonitrile (Rac-1) (1.62 g, 55%) The desired compound was obtained as an off-white solid. **^{1}H NMR** (400 MHz, $CDCl_3$) δ 8.08 (d, J = 8.5 Hz, 2H), 7.85 (d, J = 8.5 Hz, 2H), 3.02 – 2.87 (m, 1H), 2.71 (d, J = 7.0 Hz, 1H), 2.11 (d, J = 4.7 Hz, 1H), 1.29 (d, J = 5.6 Hz, 3H). **^{13}C NMR** (100 MHz, $CDCl_3$) δ 142.96, 133.00, 128.53, 117.35, 117.32, 35.58, 16.91. **FT-IR** (cm^{-1}, neat) 2232 (C≡N, w) 1397 (S=O, s). **MS** (*m/z*) 222.9 (APCI, M^+, 7,7%). No separation was observed with chiral HPLC most likely due to the inclusion complex which is effected by the location of the benzene substituent, peak observed at 38.8 min.[10]

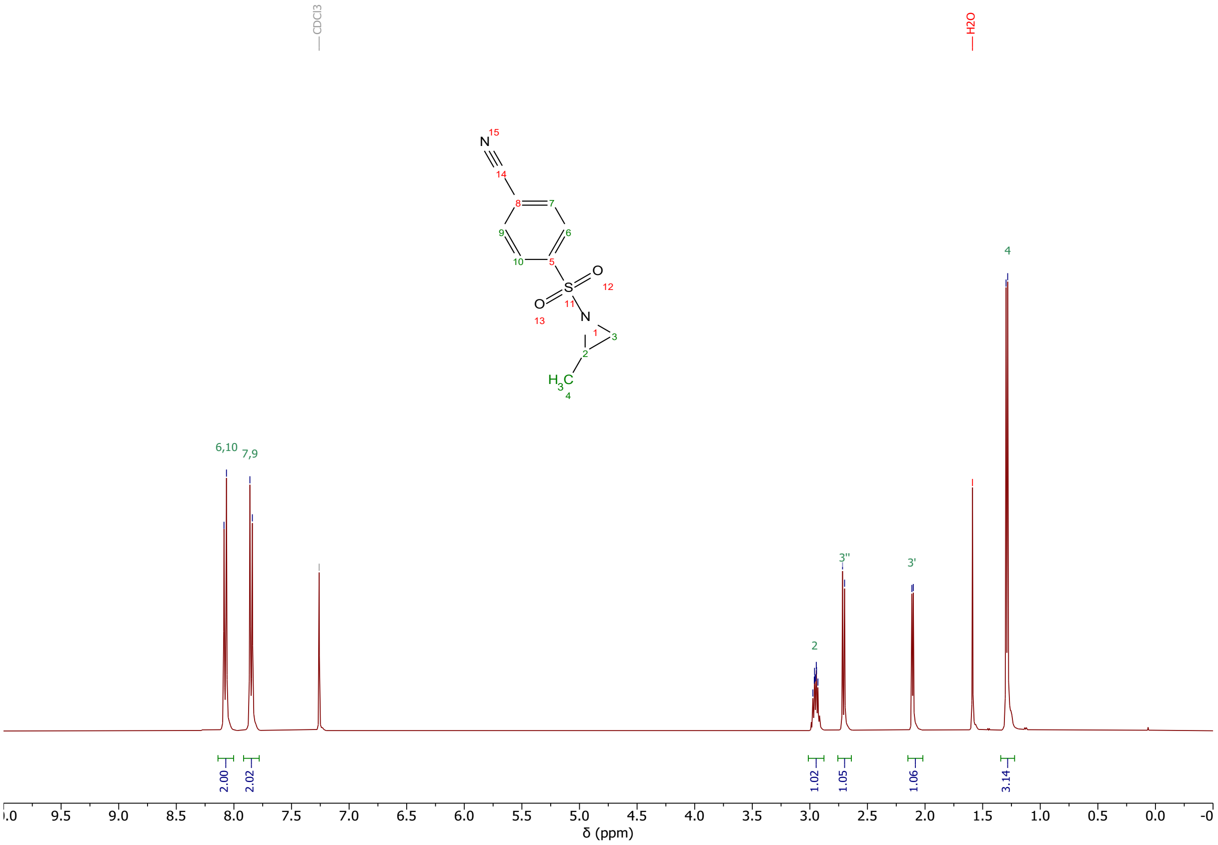

Figure S1. ^{1}H NMR of rac-1 monomer at 20 °C (400 MHz, $CDCl_3$).

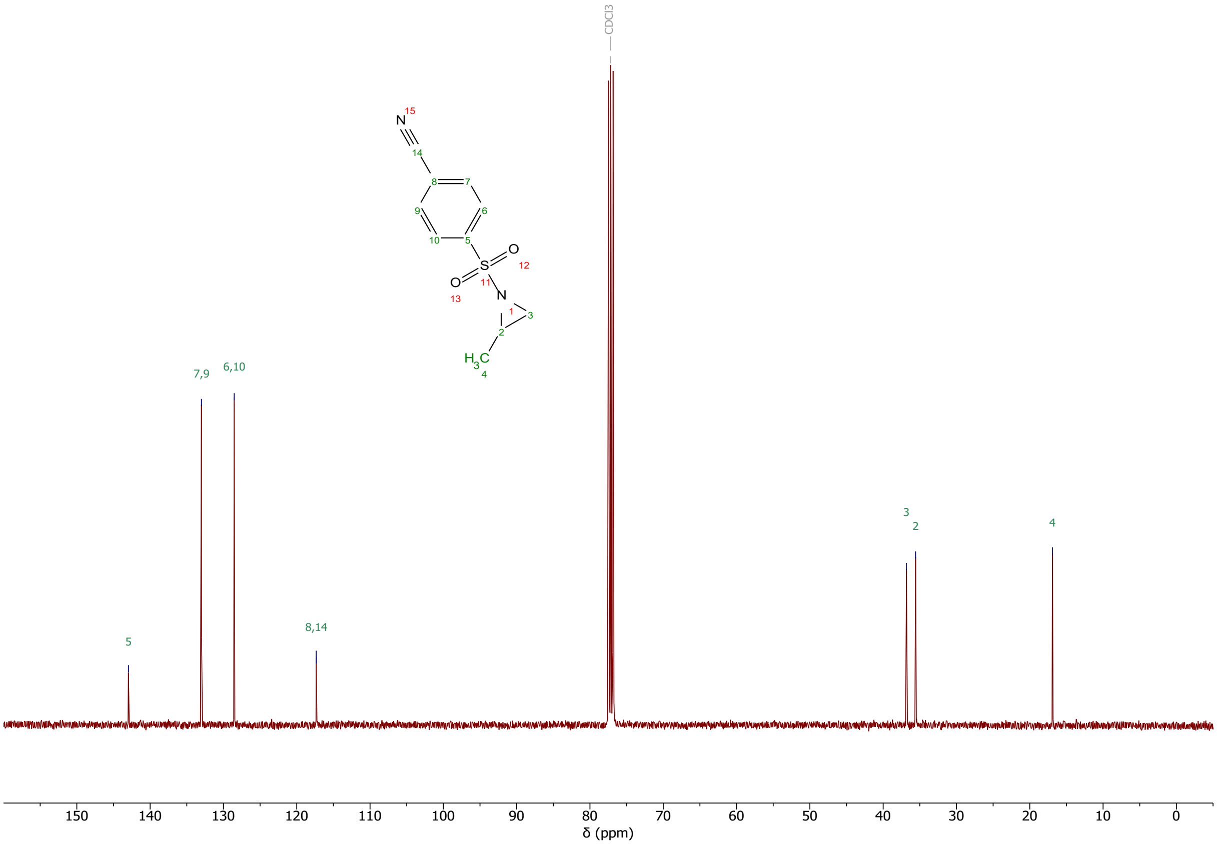

Figure S2. ^{13}C NMR of rac-1 monomer at 20 °C (100 MHz, $CDCl_3$).

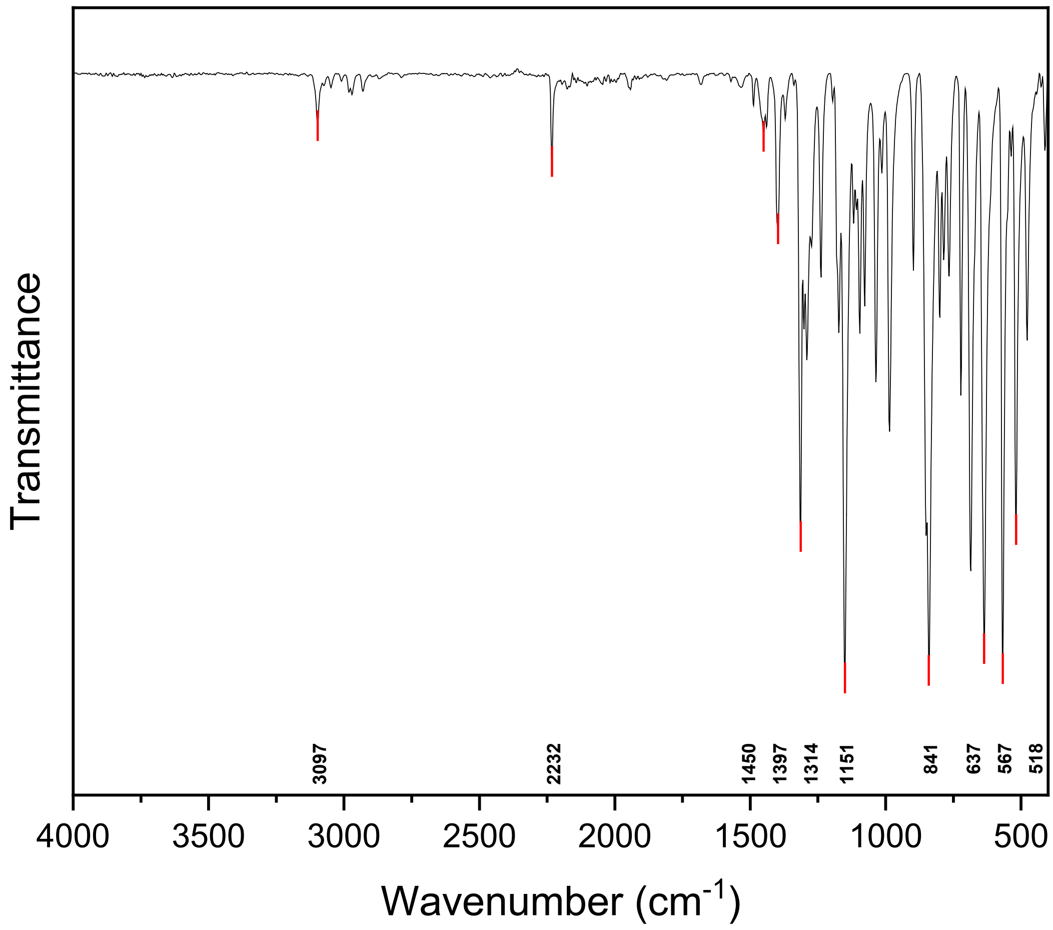

Figure S3. FTIR of rac-1 monomer at 20 °C (solid).

(R)-4-((2-methylaziridin-1-yl)sulfonyl)benzonitrile (R-1) (2.16 g, 73%) The desired compound was obtained as an off-white solid. **^{1}H NMR** (400 MHz, $CDCl_3$) δ 8.07 (d, J = 8.3 Hz, 2H), 7.85 (d, J = 8.3 Hz, 2H), 2.99 – 2.89 (m, 1H), 2.71 (d, J = 7.0 Hz, 1H), 2.11 (d, J = 4.7 Hz, 1H), 1.29 (d, J = 5.6 Hz, 3H). **^{13}C NMR** (100 MHz, $CDCl_3$) δ 142.95, 133.00, 128.53, 117.34, 117.32, 36.84, 35.57, 16.91. **FT-IR** (cm^{-1}, neat) 2234 (C≡N, w) 1320 (S=O, s). **MS** (*m/z*) 222.6 (APCI, M^+, 2.6%), 222.7 (APCI, M^+, 8.0%), 222.8 (APCI, M^+, 2.8%). $[\alpha]_D^{21}$= -17.4 (*c*=86.0, MeCN). No separation was observed with chiral HPLC most likely due to the inclusion complex which is effected by the location of the benzene substituent, peak observed at 38.7 min.[10]

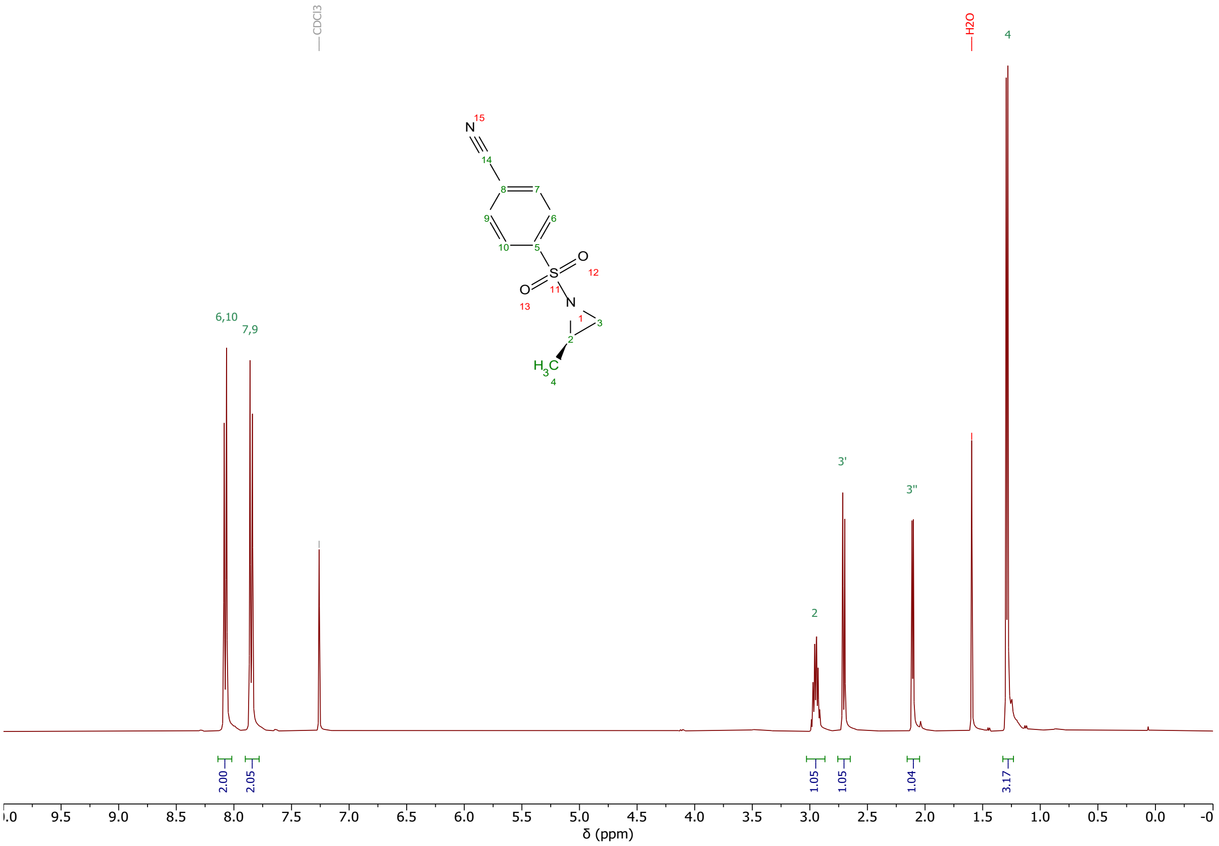

Figure S4. ^{1}H NMR of R-1 monomer at 20 °C (400 MHz, $CDCl_3$).

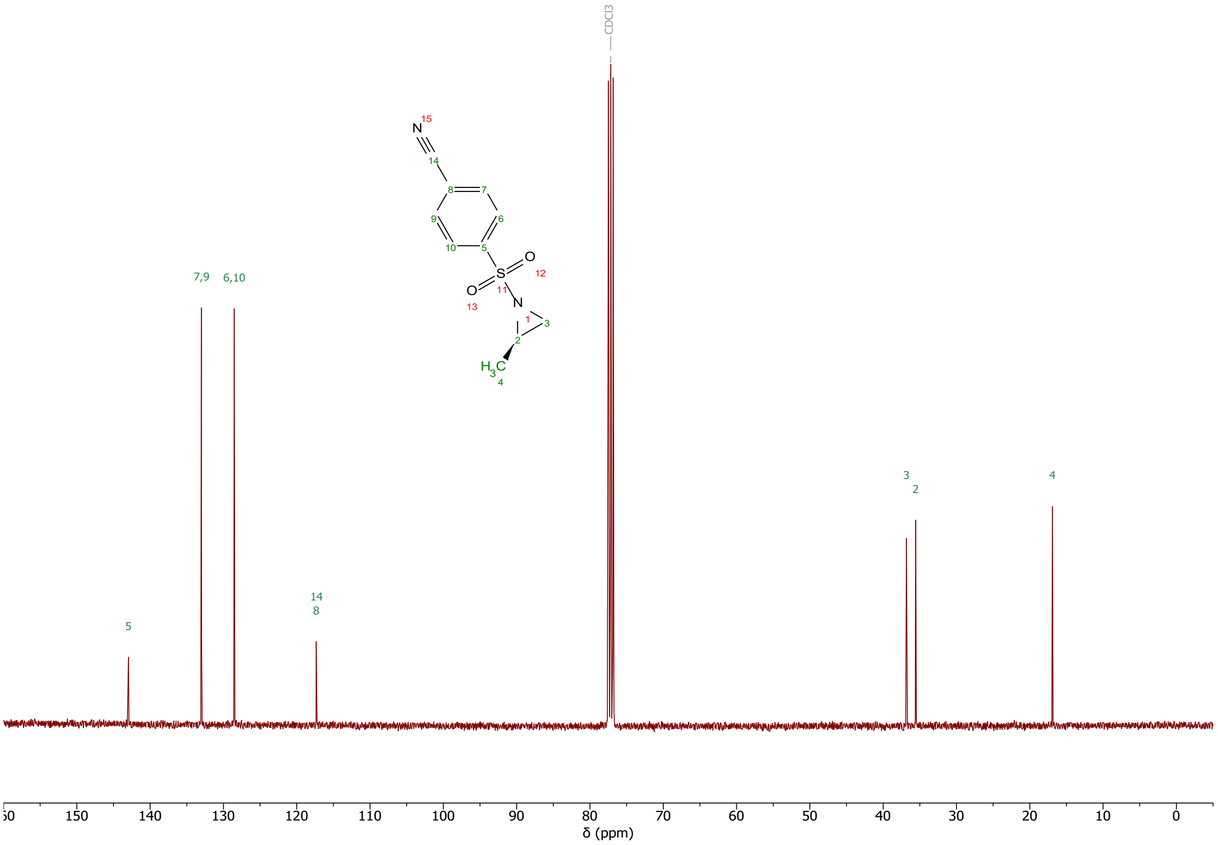

Figure S5. ^{13}C NMR of R-1 monomer at 20 °C (100 MHz, $CDCl_3$).

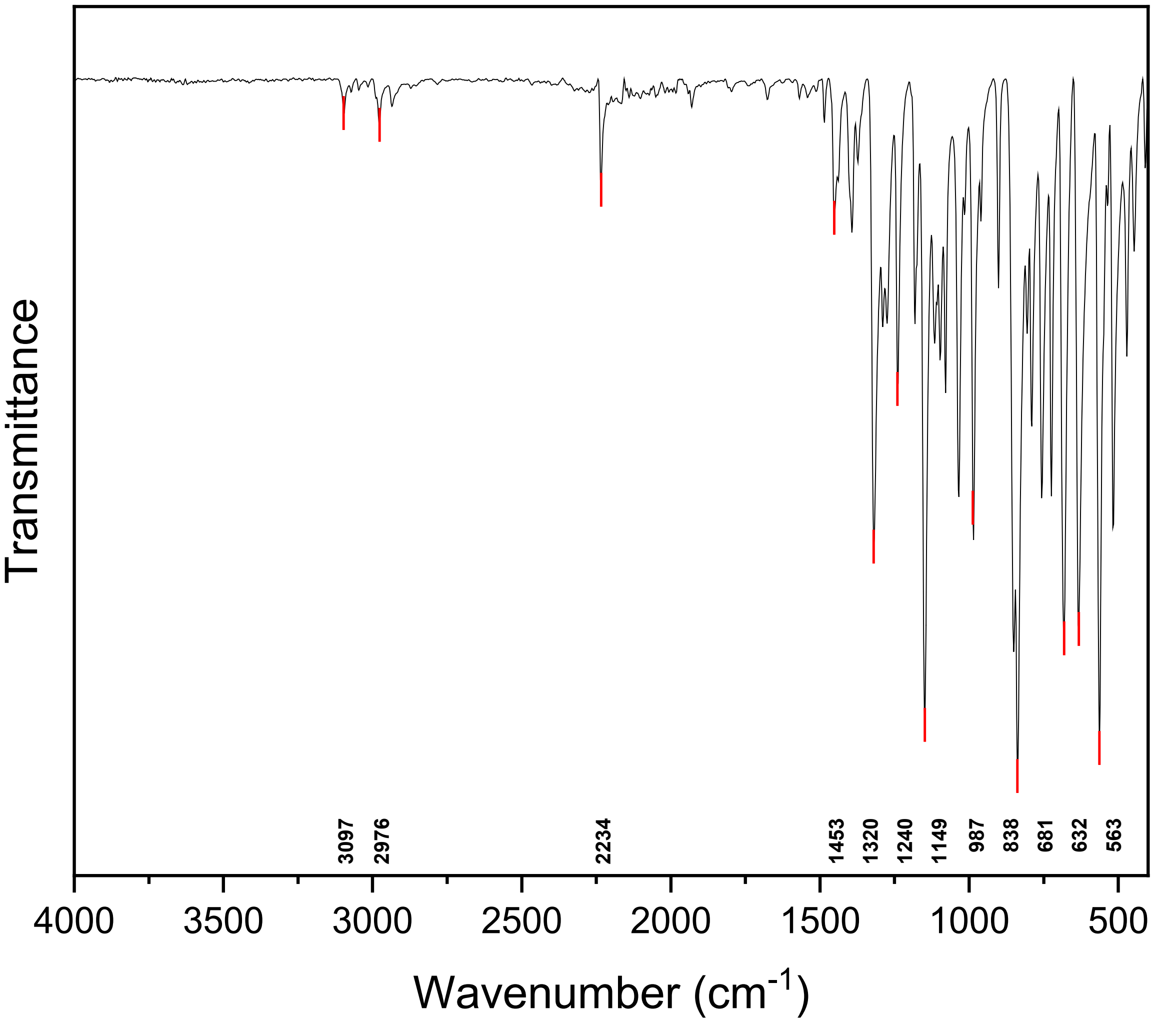

Figure S6. FTIR of R-1 monomer at 20 °C (solid).

(S)-4-((2-methylaziridin-1-yl)sulfonyl)benzonitrile (S-1) (2.53 g, 85%) The desired compound was obtained as an off-white solid. **^{1}H NMR** (400 MHz, $CDCl_3$) δ 8.16 (d, J = 7.8, 1.5 Hz, 1H), 7.94 – 7.87 (d, 1H), 7.76 (m, J = 16.6, 7.6, 1.4 Hz, 2H), 3.14 (m, J = 7.2, 5.4 Hz, 1H), 2.93 (d, J = 7.0 Hz, 1H), 2.23 (d, J = 4.8 Hz, 1H), 1.34 (d, J = 5.6 Hz, 3H). **^{13}C NMR** (100 MHz, $CDCl_3$) δ 141.51, 135.50, 133.38, 133.08, 129.82, 116.02, 111.57, 37.75, 36.54, 17.00. **FT-IR** (cm^{-1}, neat) 2234 (C≡N, w). **MS** (*m/z*) 222.9 (APCI, M^+, 4.3%). $[\alpha]_D^{21}$= +17.8 (*c*=54.0, MeCN). No separation was observed with chiral HPLC most likely due to the inclusion complex which is effected by the location of the benzene substituent, peak observed at 38.8 min.[10]

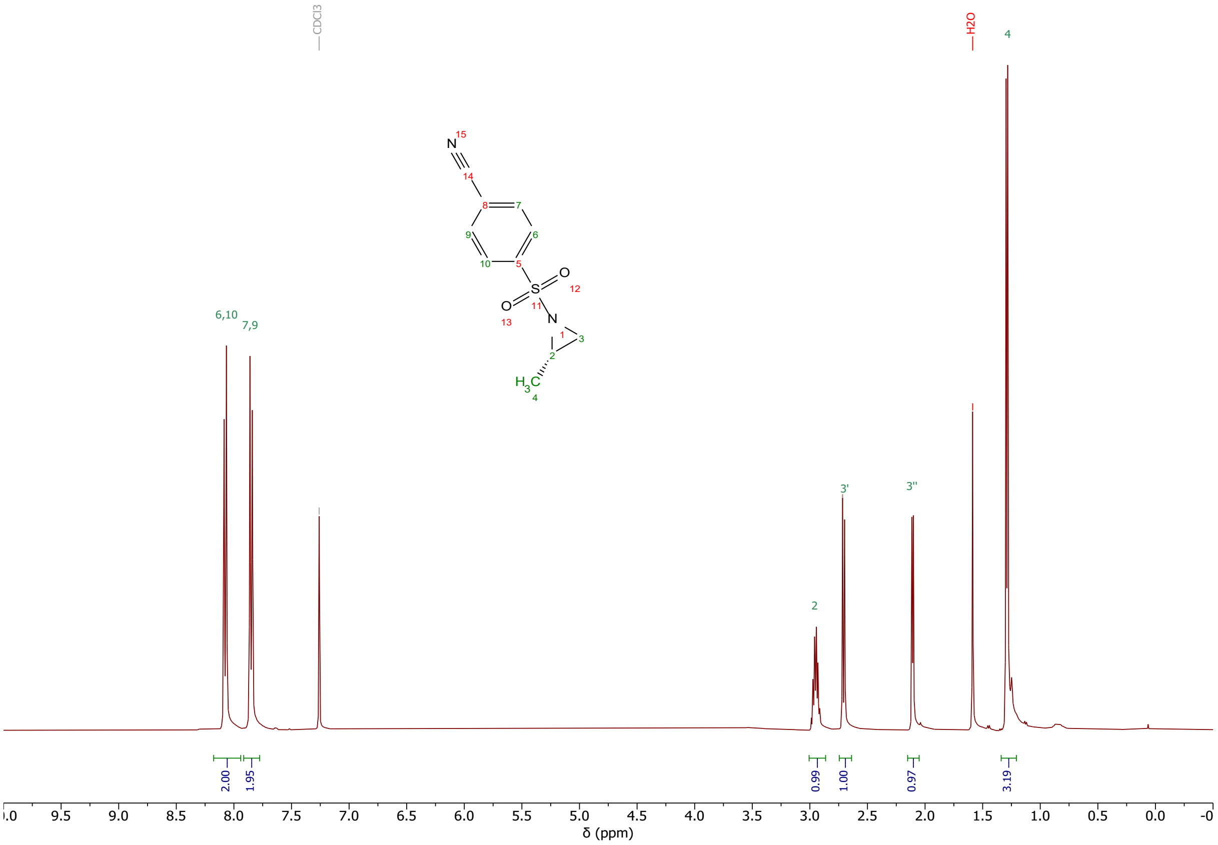

Figure S7. ^{1}H NMR of R-1 monomer at 20 °C (400 MHz, $CDCl_3$).

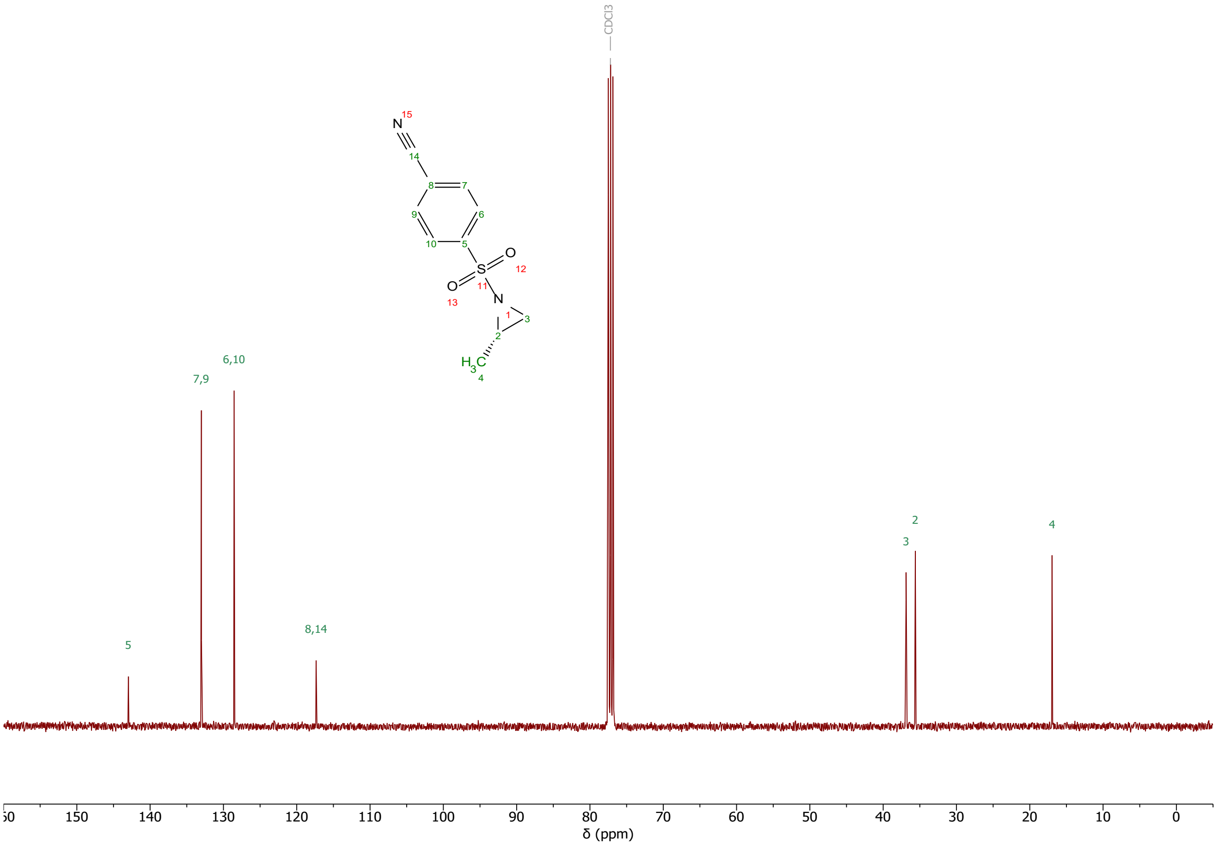

Figure S8. ^{13}C NMR of R-1 monomer at 20 °C (100 MHz, $CDCl_3$).

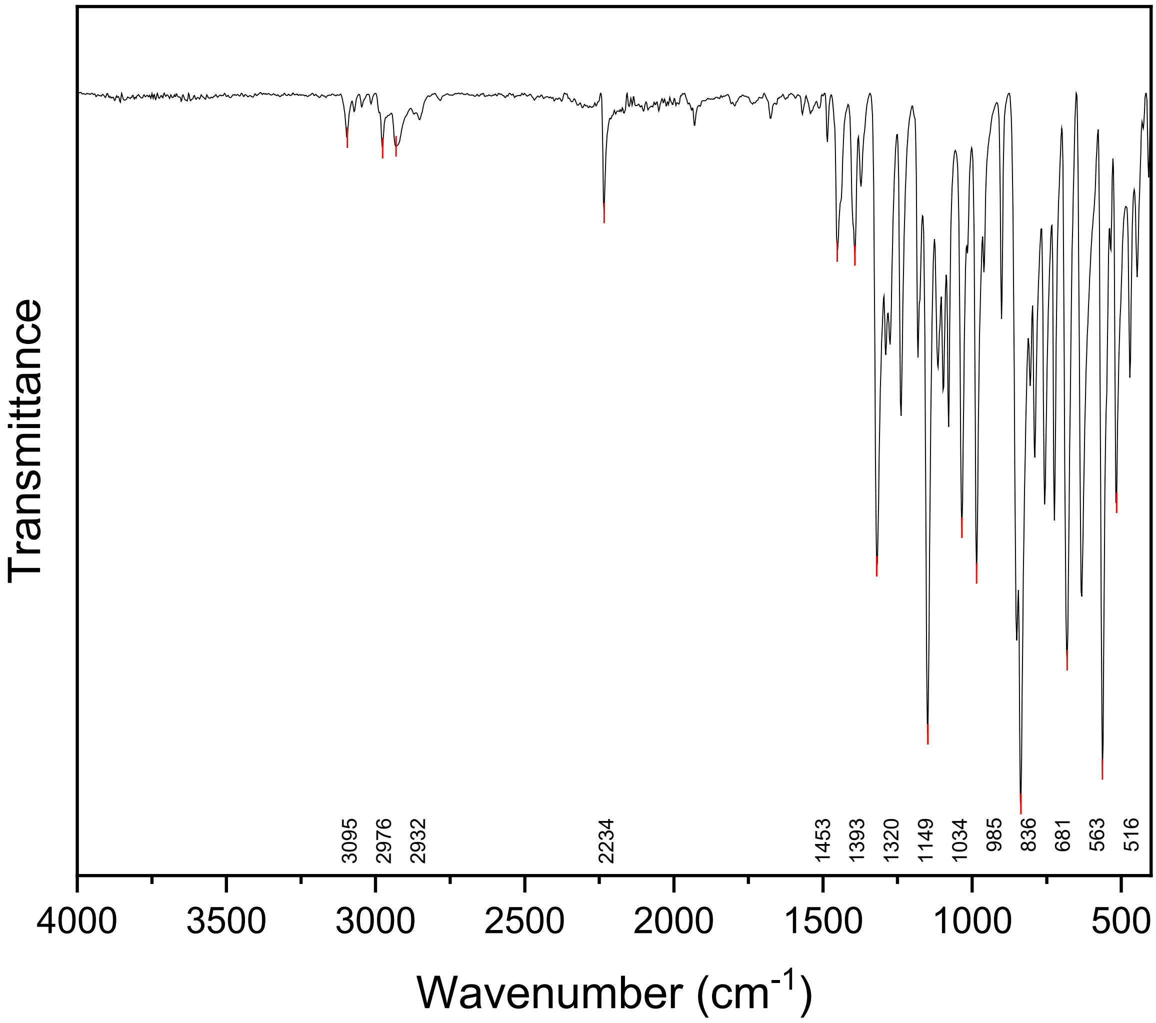

Figure S9. FTIR of R-1 monomer at 20 °C (solid).

2-((2-methylaziridin-1-yl)sulfonyl)benzonitrile (Rac-2) (1.24 g, 42%) The desired compound was obtained as an off-white solid. **^{1}H NMR** (400 MHz, $CDCl_3$) δ 8.10 (d, J = 7.7 Hz, 1H), 7.84 (d, J = 7.3 Hz, 1H), 7.70 (t, J = 7.7 Hz, 2H), 3.07 (m, J = 5.5 Hz, 1H), 2.87 (d, J = 7.1 Hz, 1H), 2.17 (d, 1H), 1.28 (d, J = 5.5 Hz, 3H). **^{13}C NMR** (100 MHz, $CDCl_3$) δ 141.19, 135.19, 133.07, 132.76, 129.50, 115.70, 111.25, 37.43, 36.22, 16.68. **FT-IR** (cm^{-1}, neat) 2232 (C≡N, w). **MS** (*m/z*) 222.8 (APCI, M^+, 1.6%), 222.9 (APCI, M^+, 1.1%).

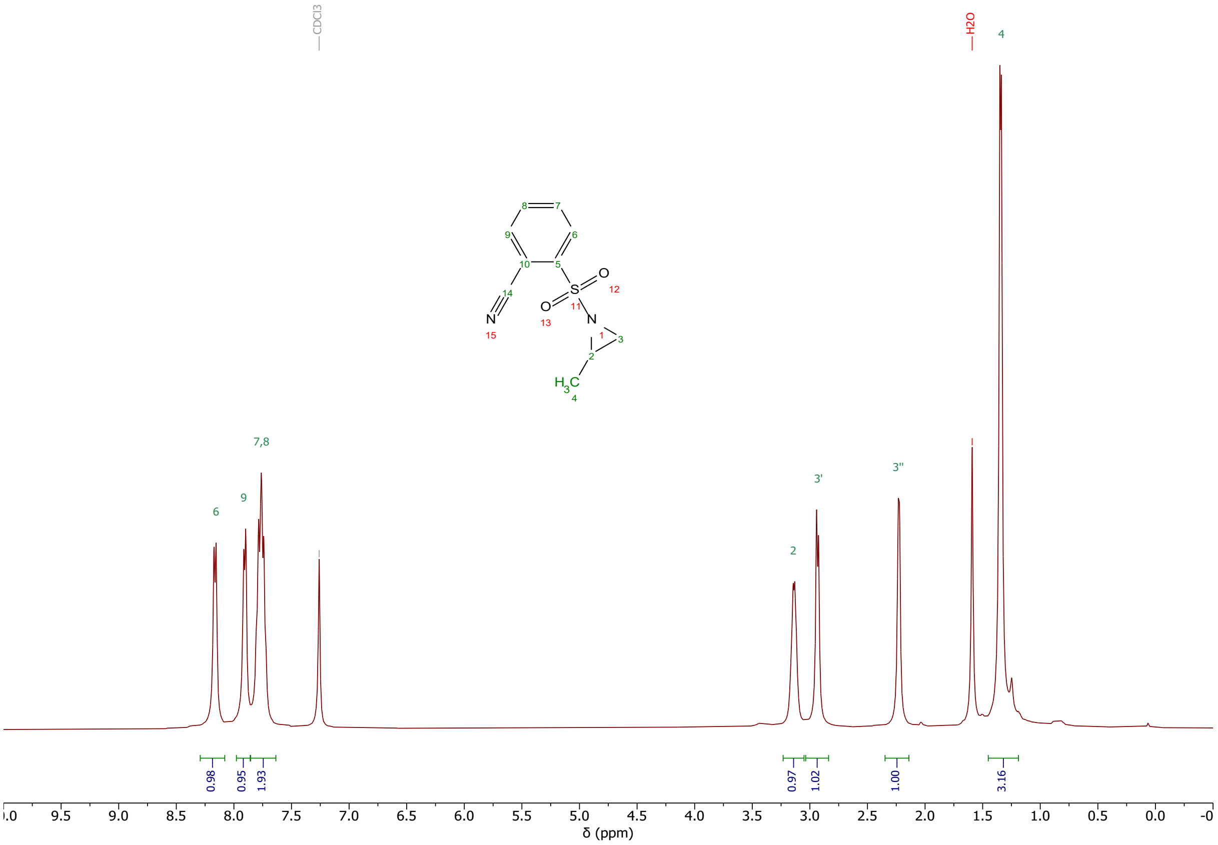

Figure S10. ^{1}H NMR of rac-2 monomer at 20 °C (400 MHz, $CDCl_3$).

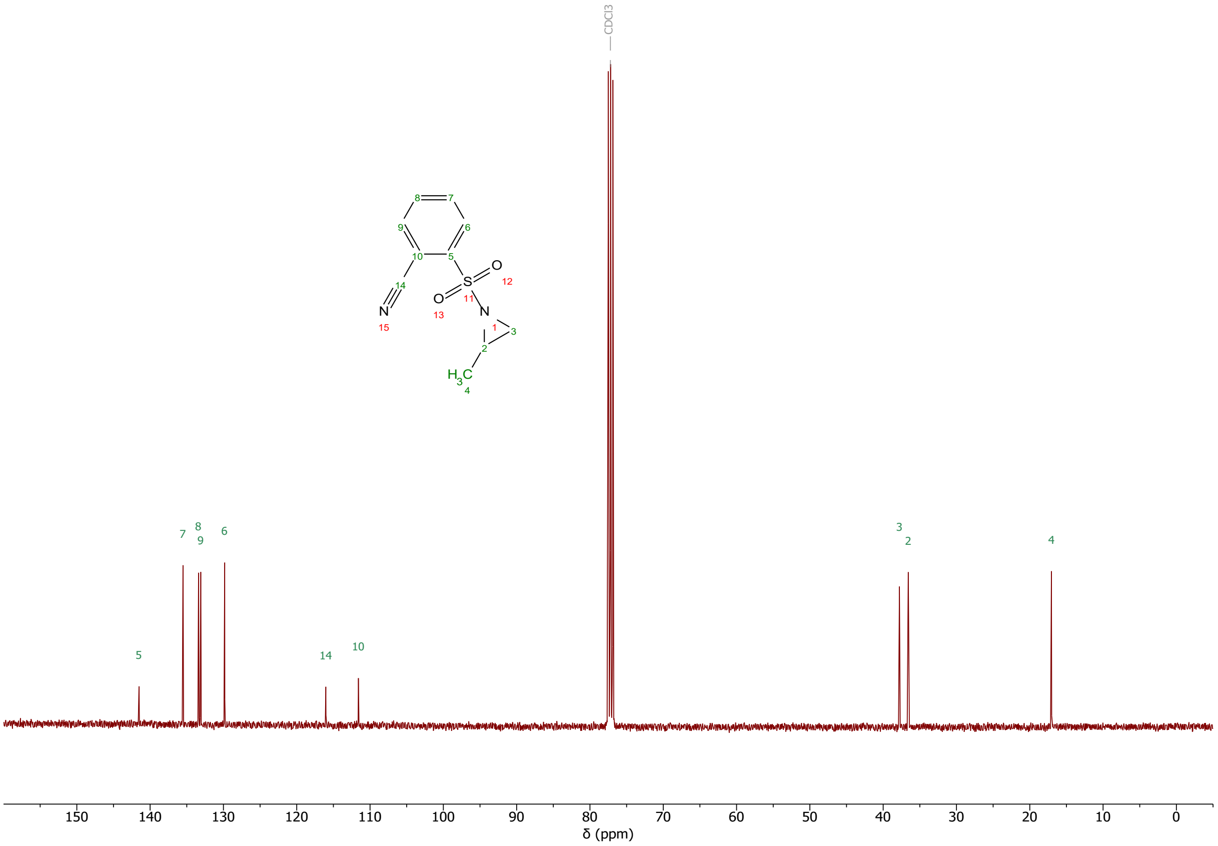

Figure S11. ^{13}C NMR of rac-2 monomer at 20 °C (100 MHz, $CDCl_3$).

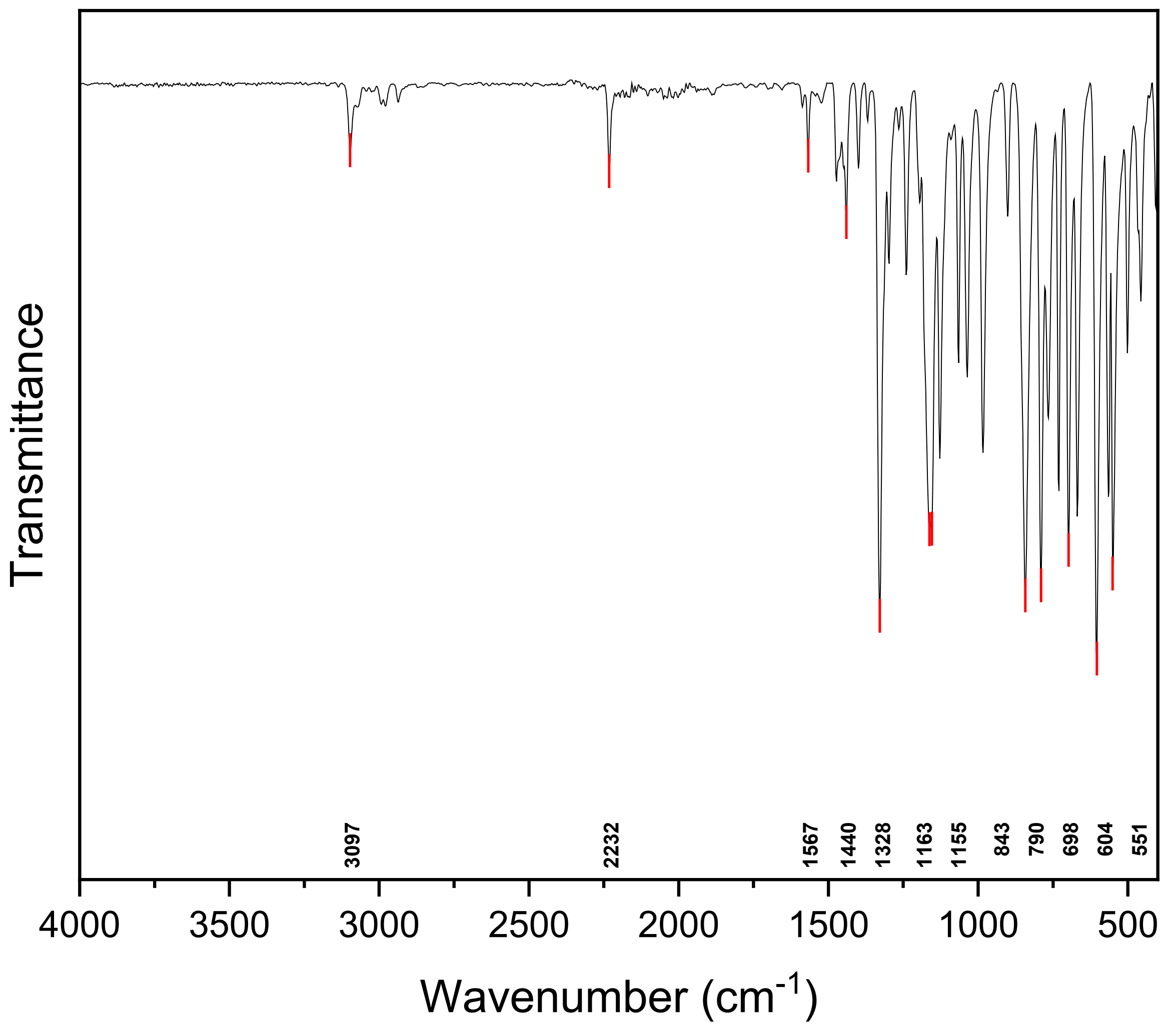

Figure S12. FTIR of rac-2 monomer at 20 °C (solid).

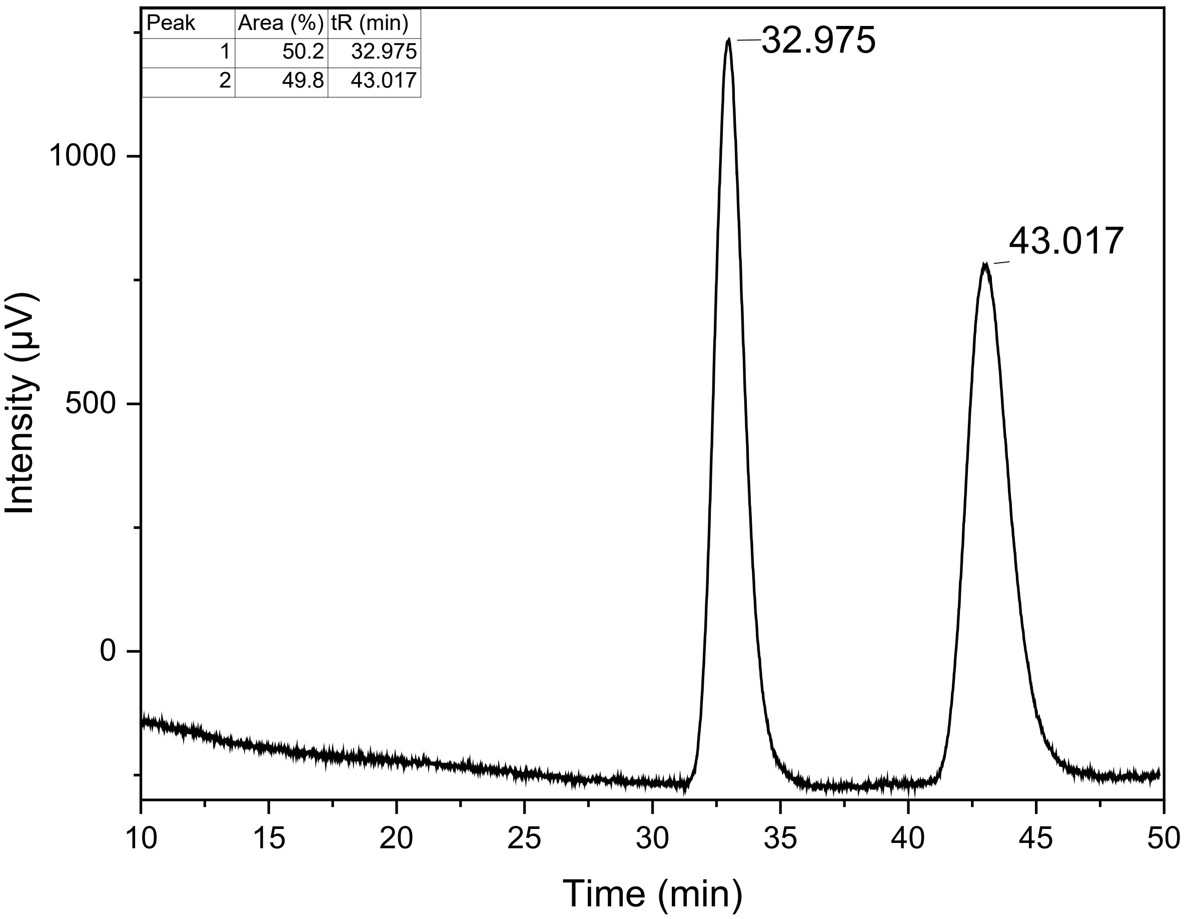

Figure S13. Chiral HPLC trace of rac-2, showing baseline separation of the two enantiomers at 33.0 and 43.0 min.

(R)-2-((2-methylaziridin-1-yl)sulfonyl)benzonitrile (R-2) (416 mg, 35%) The desired compound was obtained as am off-white solid. **^{1}H NMR** (400 MHz, $CDCl_3$) δ 8.16 (d, J = 7.8 Hz, 1H), 7.90 (d, 1H), 7.81 – 7.72 (t, 2H), 3.18 – 3.09 (m, 1H), 2.93 (d, J = 7.0 Hz, 1H), 2.22 (d, 1H), 1.34 (d, J = 5.6, 0.9 Hz, 3H). **^{13}C NMR** (100 MHz, $CDCl_3$) δ 141.49, 135.50, 133.39, 133.08, 129.81, 116.02, 111.56, 37.74, 36.53, 17.00. **FT-IR** (cm^{-1}, neat) 2231 (C≡N, w). **MS** (*m/z*) 222.8 (M+H, 1.2%), 222.9 (APCI, M^+, 2.0%). %). $[\alpha]_D^{21}$= -34.4 (*c*=20.0, MeCN).

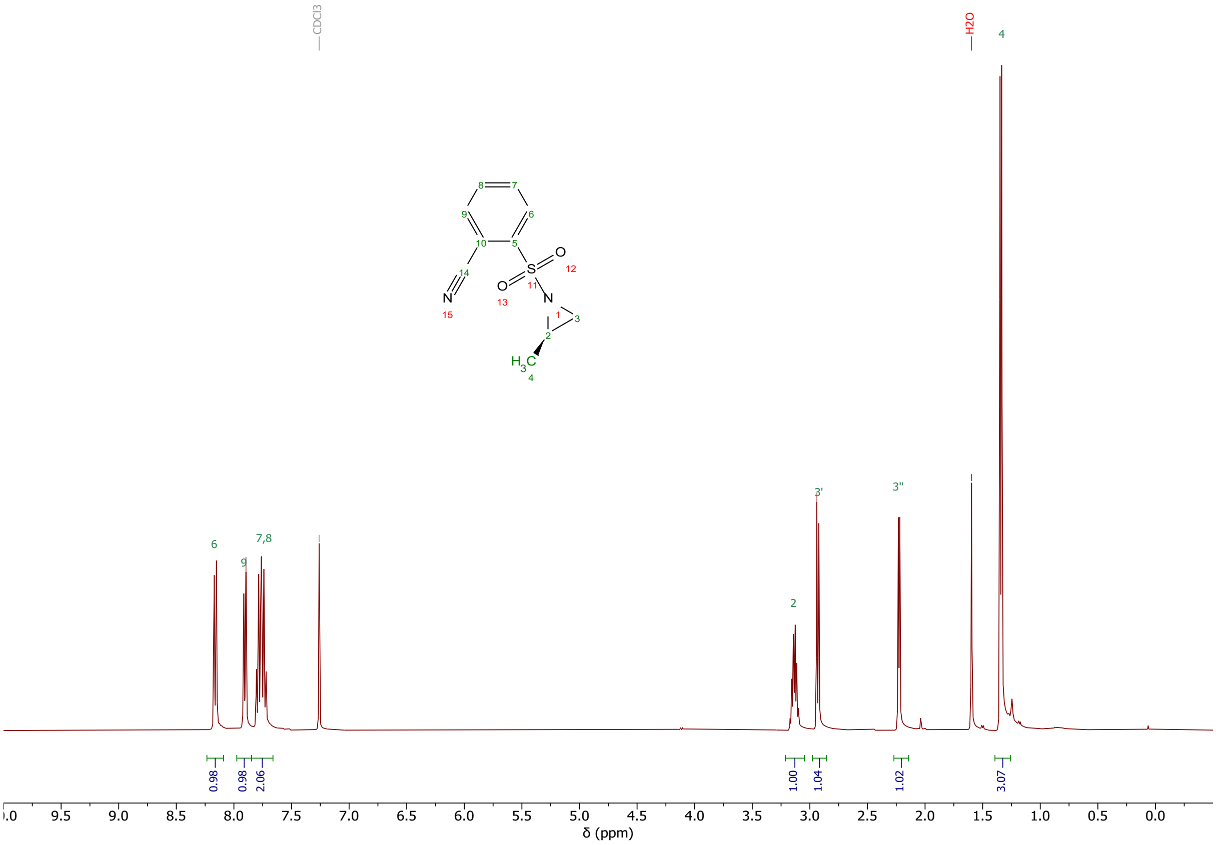

Figure S14. ^{1}H NMR of R-1 monomer at 20 °C (400 MHz, $CDCl_3$).

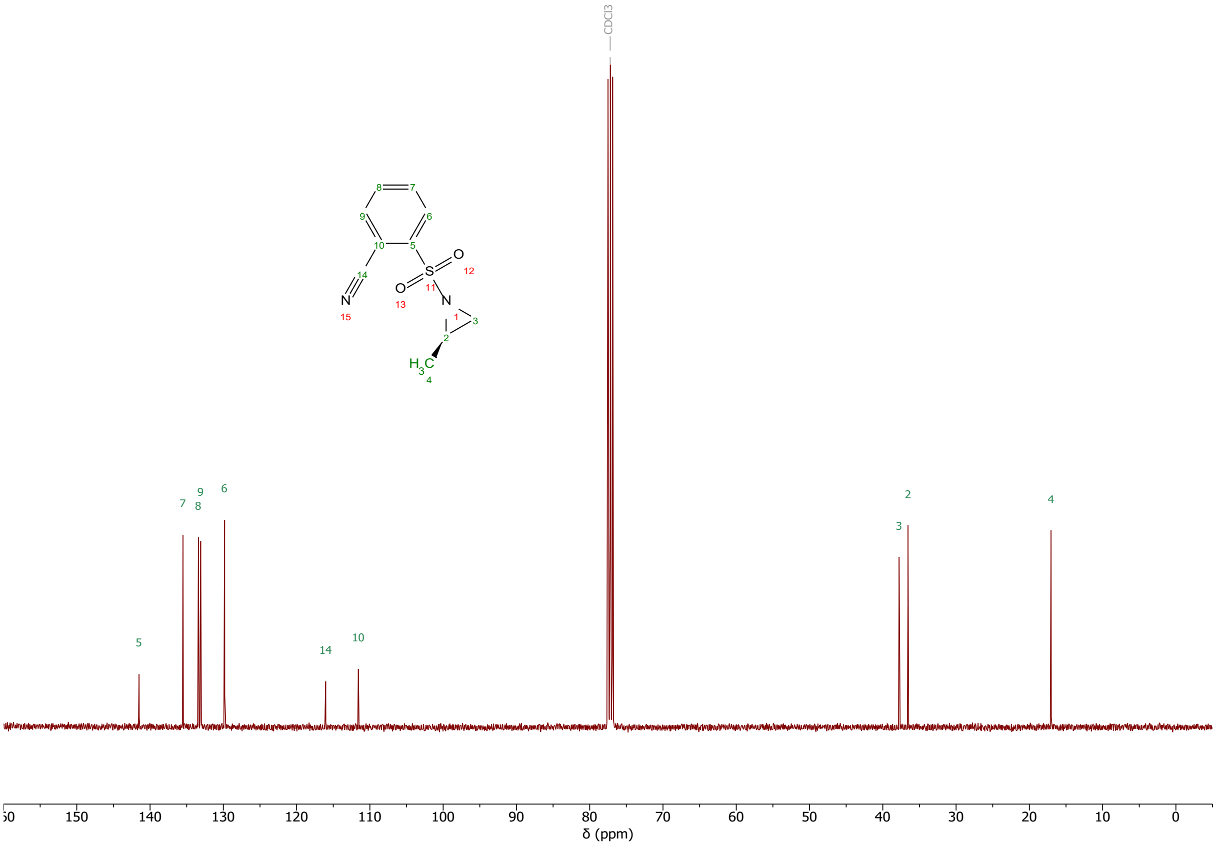

Figure S15. ^{13}C NMR of R-1 monomer at 20 °C (100 MHz, $CDCl_3$).

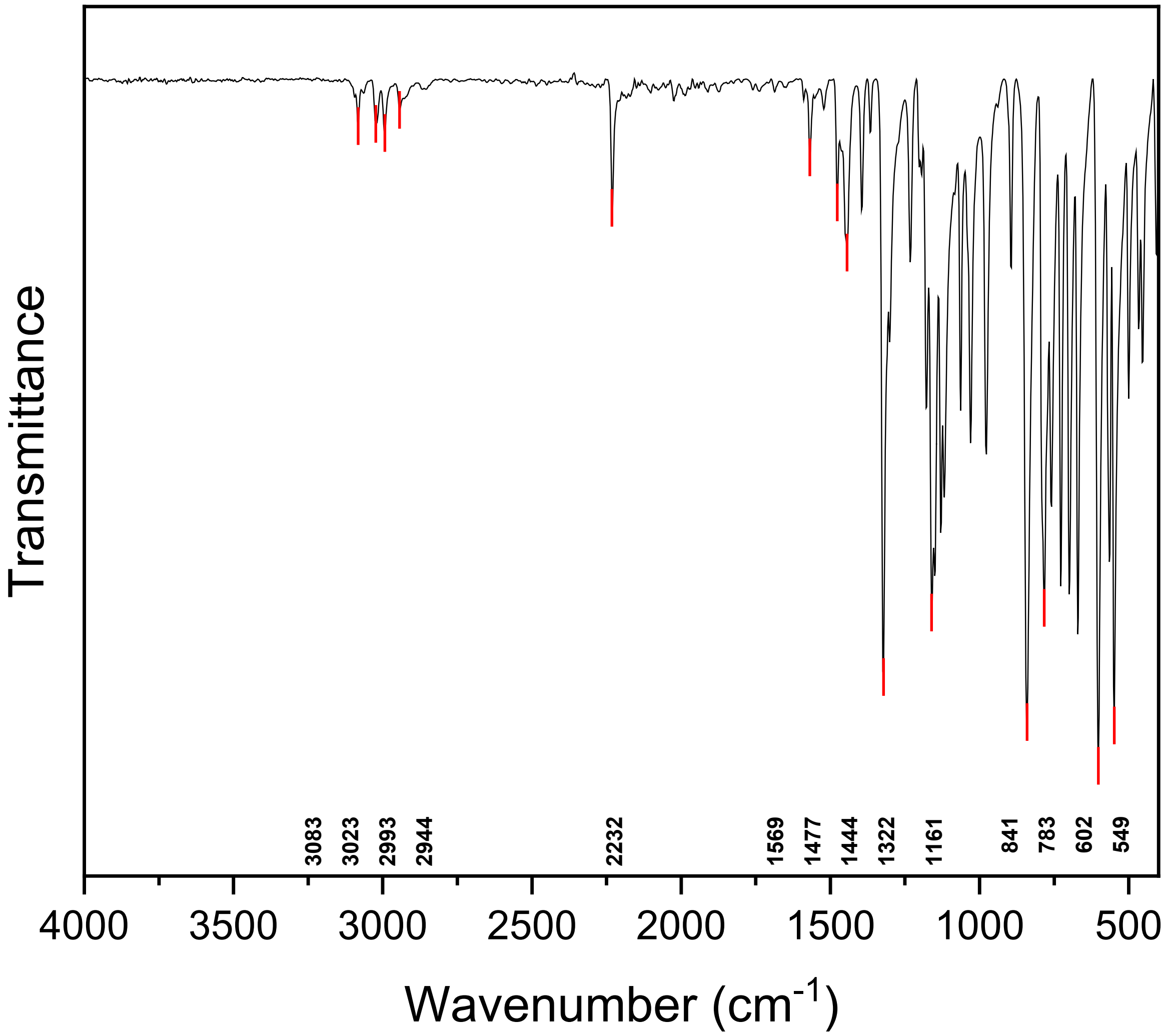

Figure S16. FTIR of R-1 monomer at 20 °C (solid).

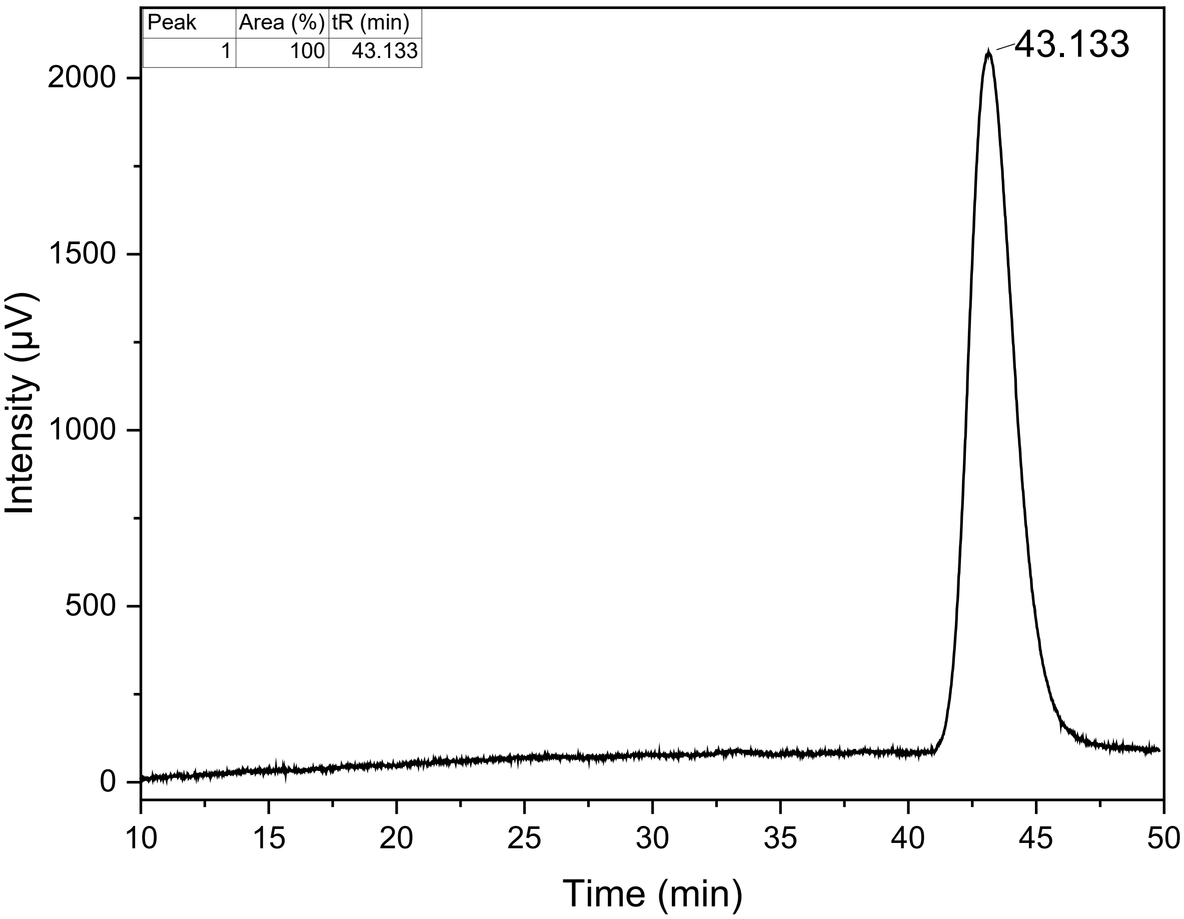

Figure S17. Chiral HPLC trace of R-2, showing the R enantiomers at 43.0 min.

(S)-2-((2-methylaziridin-1-yl)sulfonyl)benzonitrile (S-2) (718 mg, 61%) The desired compound was obtained as an off-white solid. **^{1}H NMR** (400 MHz, $CDCl_3$) δ 8.16 (d, J = 7.8, 1.5 Hz, 1H), 7.94 – 7.87 (d, 1H), 7.81 – 7.72 (m, 2H), 3.14 (m, J = 7.1, 5.3 Hz, 1H), 2.93 (d, J = 7.0 Hz, 1H), 2.23 (d, J = 4.8 Hz, 1H), 1.34 (d, J = 5.6 Hz, 3H). **^{13}C NMR** (100 MHz, $CDCl_3$) δ 141.51, 135.50, 133.38, 133.08, 129.82, 116.02, 111.57, 37.75, 36.54, 17.00. **FT-IR** (cm^{-1}, neat) 2231 (C≡N, w). **MS** (*m/z*) 222.8 (APCI, M^+, 4.1%). $[\alpha]_D^{21}$= +34.0 (*c*=14.0, MeCN).

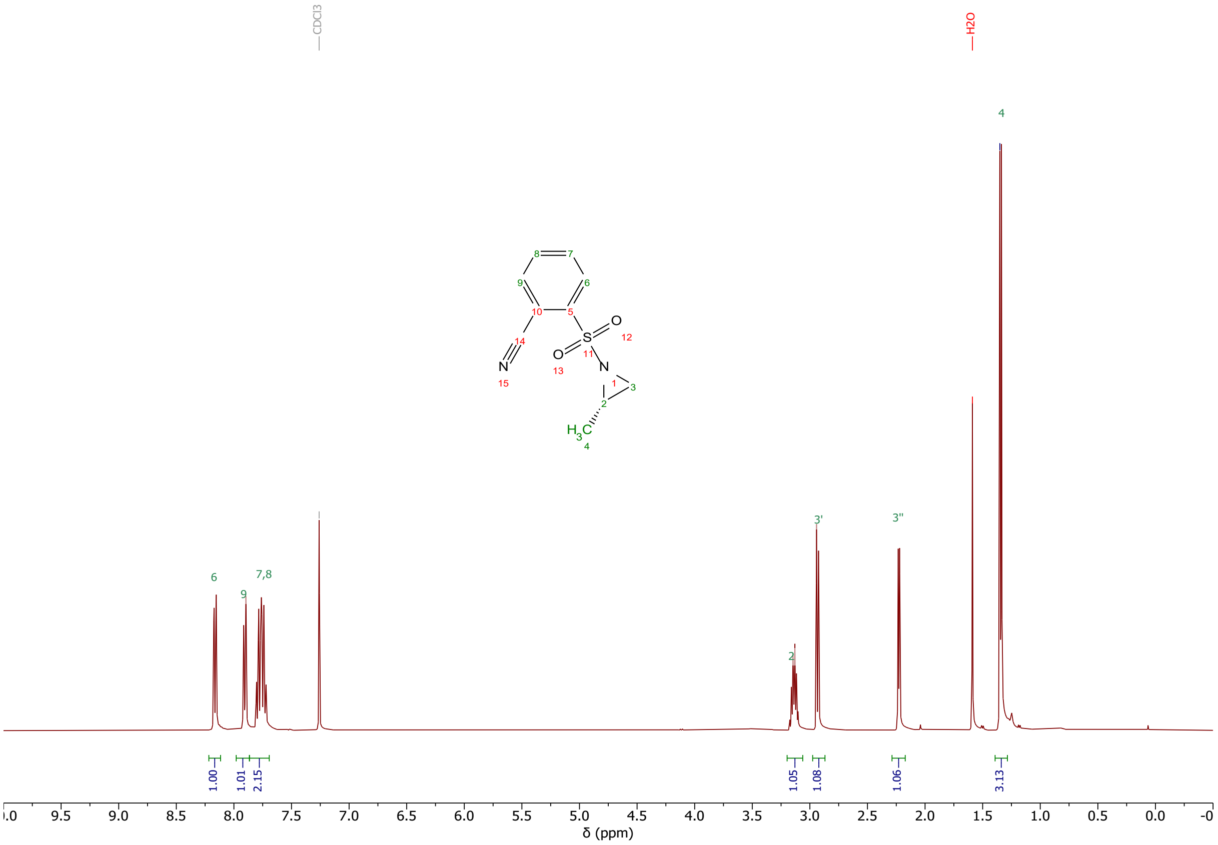

Figure S18. ^{1}H NMR of R-1 monomer at 20 °C (400 MHz, $CDCl_3$).

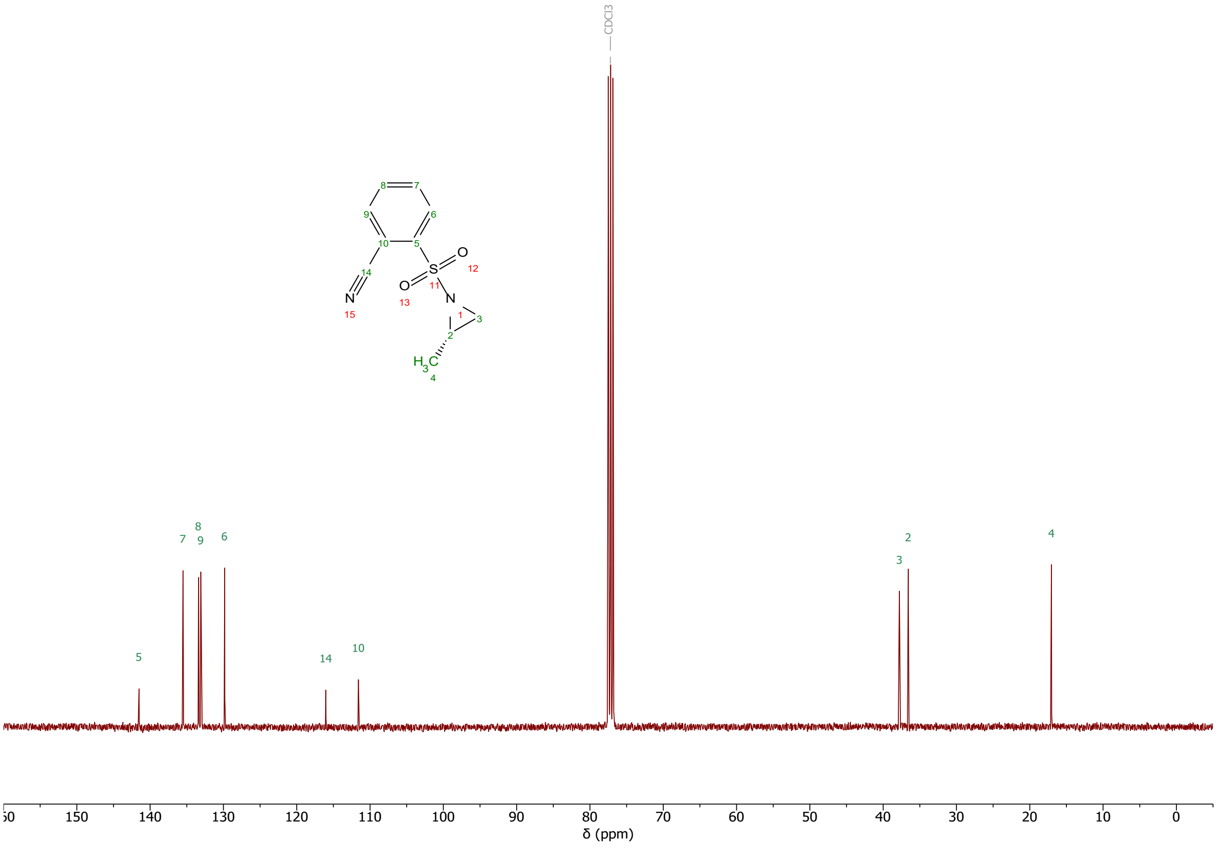

Figure S19. ^{13}C NMR of R-1 monomer at 20 °C (100 MHz, $CDCl_3$).

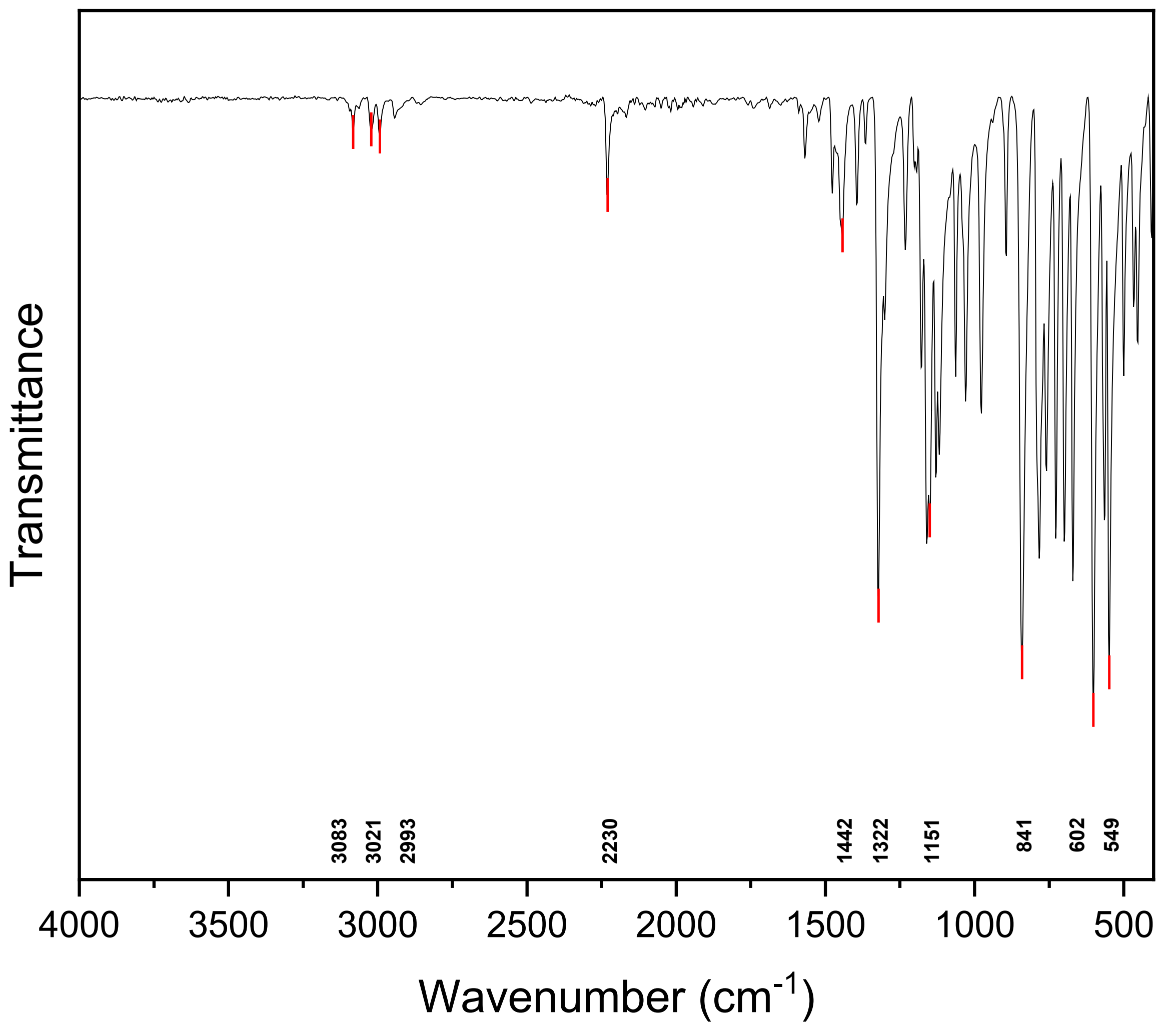

Figure S20. FTIR of R-1 monomer at 20 °C (solid).

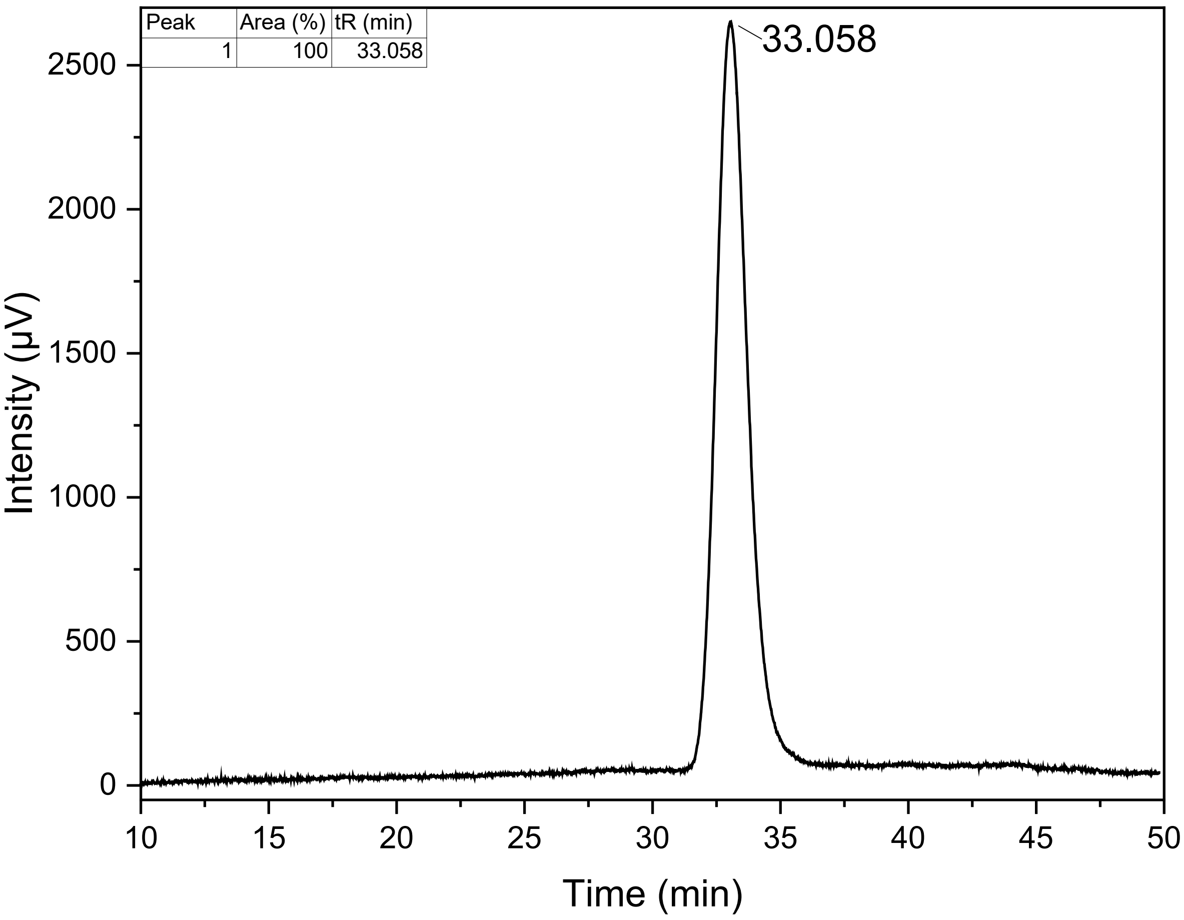

Figure S21. Chiral HPLC trace of S-2, showing the S enantiomers at 33.0 min.

Monomer stability

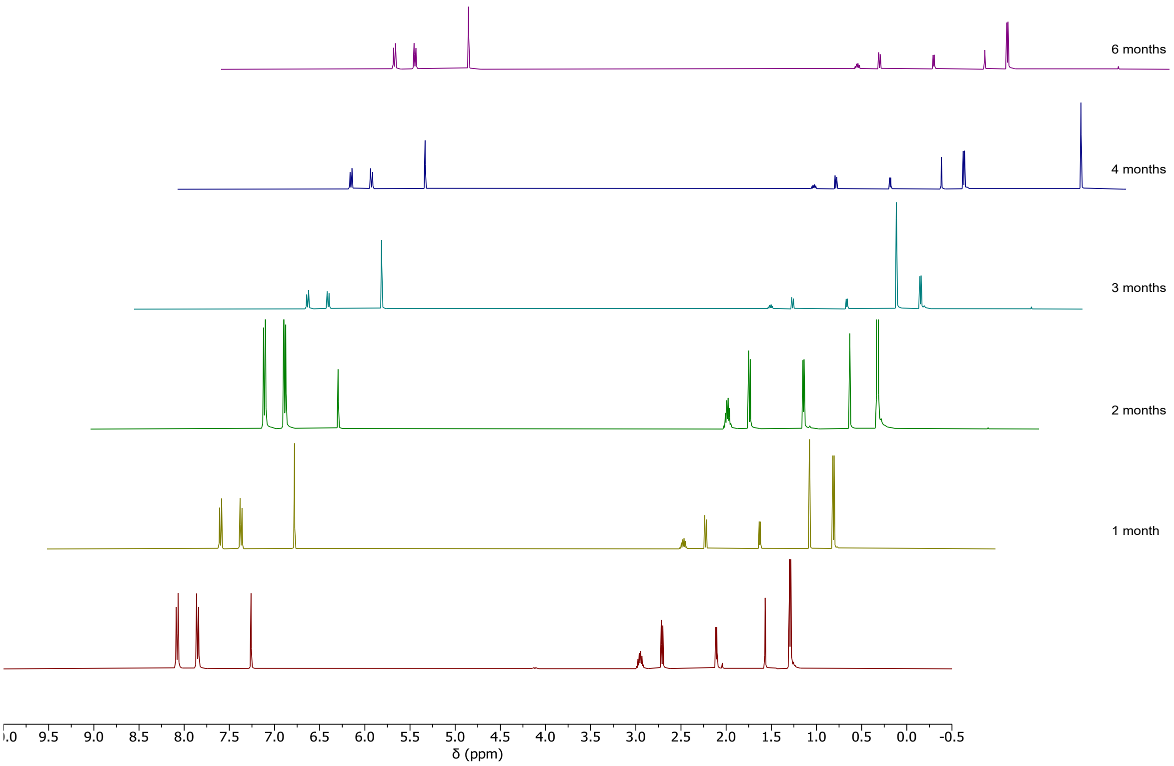

Figure S22. Monomer stability monitored on ^{1}H NMR over six months for R-1 (400 MHz, $CDCl_3$).

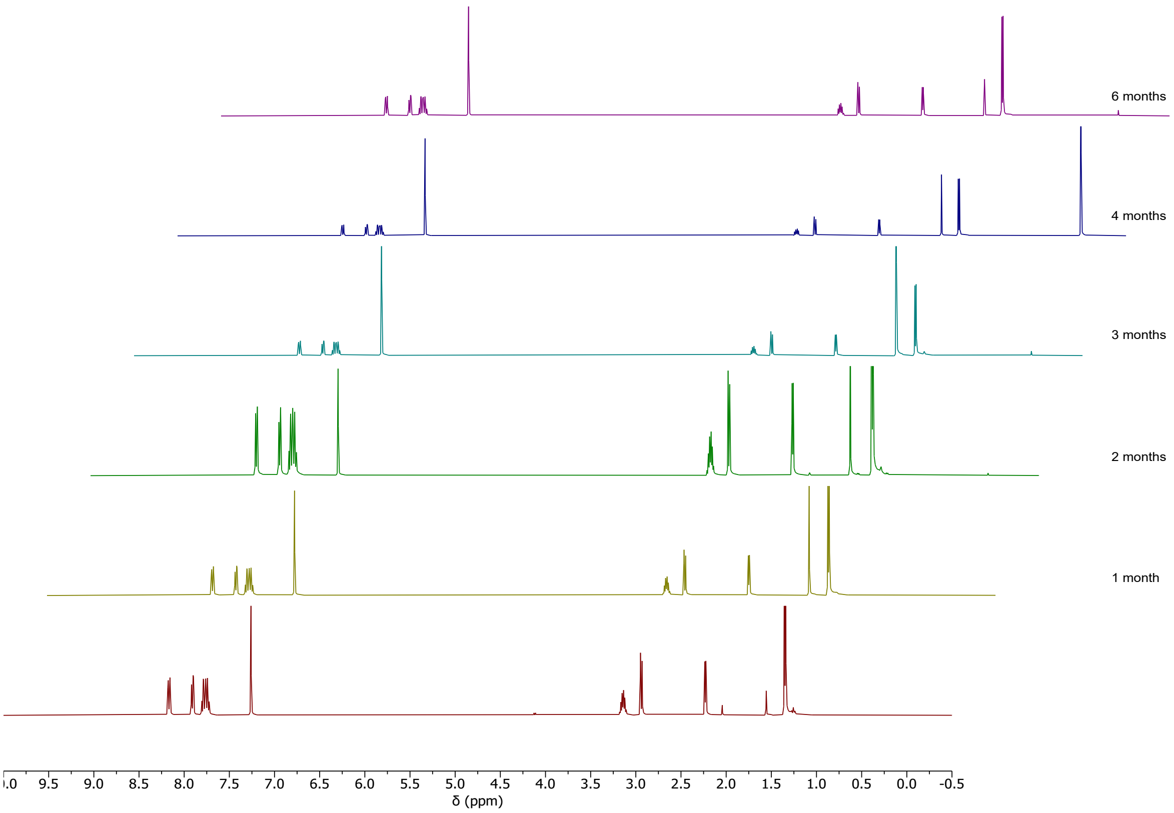

Figure S23. Monomer stability monitored on ^{1}H NMR over six months for S-2 (400 MHz, $CDCl_3$).

Anionic Polymerization of Activated Aziridines

Short chains (60-150 DP):

A vial with a septum screw cap, equipped with magnetic stirrer, was heat-gun dried under vacuum and then purged with nitrogen. The vial was charged with monomer (1.8 mmol (400 mg), 60 to 150 eq) in DMF (3.5 mL), while being purged with nitrogen and then heated to 60 °C (clear solution). In a separate vial, an initiator solution was made in stock. Potassium bis(trimethylsilyl)amide (2.2 eq) was dissolved in DMF (1 mL) to which N-benzylmethansulfonamide (2.0 eq) was added. The appropriate amount of initiator solution (0.5 mL) was then added to the monomer solution and the reaction mixture was stirred at 60 °C overnight. Within a few minutes after initiator addition the reaction mixture went from a clear solution to a white suspension when using a chiral monomer. To terminate the polymerization 0.5 M methanolic HCl (2.0 mL) was added and the reaction mixture was stirred for 5 min at room temperature. The reaction mixture was then suspended in methanol and collected by centrifugation as a white powder and dried at 120 °C *in vacuo*, yields: typically above 90%.

Longer chain (300 DP):

A Schlenk flask with a septum, equipped with magnetic stirrer, was heat-gun dried under vacuum and then purged with nitrogen. The vial was charged with monomer (1.8 mmol (400 mg), 300 eq) and freeze dried over benzene for 4 hours. Then the monomer was dissolved in DMF (3.5 mL) and heated to 60 °C. In a separate Schlenk flask, an initiator solution was made in stock. Potassium bis(trimethylsilyl)amide (2.2 eq) and N-benzylmethansulfonamide (2.0 eq) were dissolved in benzene and freeze dried over benzene for 4 hours. Then the initiator was dissolved in DMF (1.0 mL). The appropriate amount of initiator solution (0.5 mL) was then added to the monomer solution and the reaction mixture was stirred at 60 °C overnight. Within a few minutes after initiator addition the reaction mixture went from a clear solution to a white suspension when using a chiral monomer. To terminate the polymerization 0.5 M methanolic HCl (2.0 mL) was added and the reaction mixture was stirred for 5 min at room temperature. The reaction mixture was then suspended in methanol and collected by centrifugation as a white powder and dried at 120 °C *in vacuo*, yields: typically above 90%.

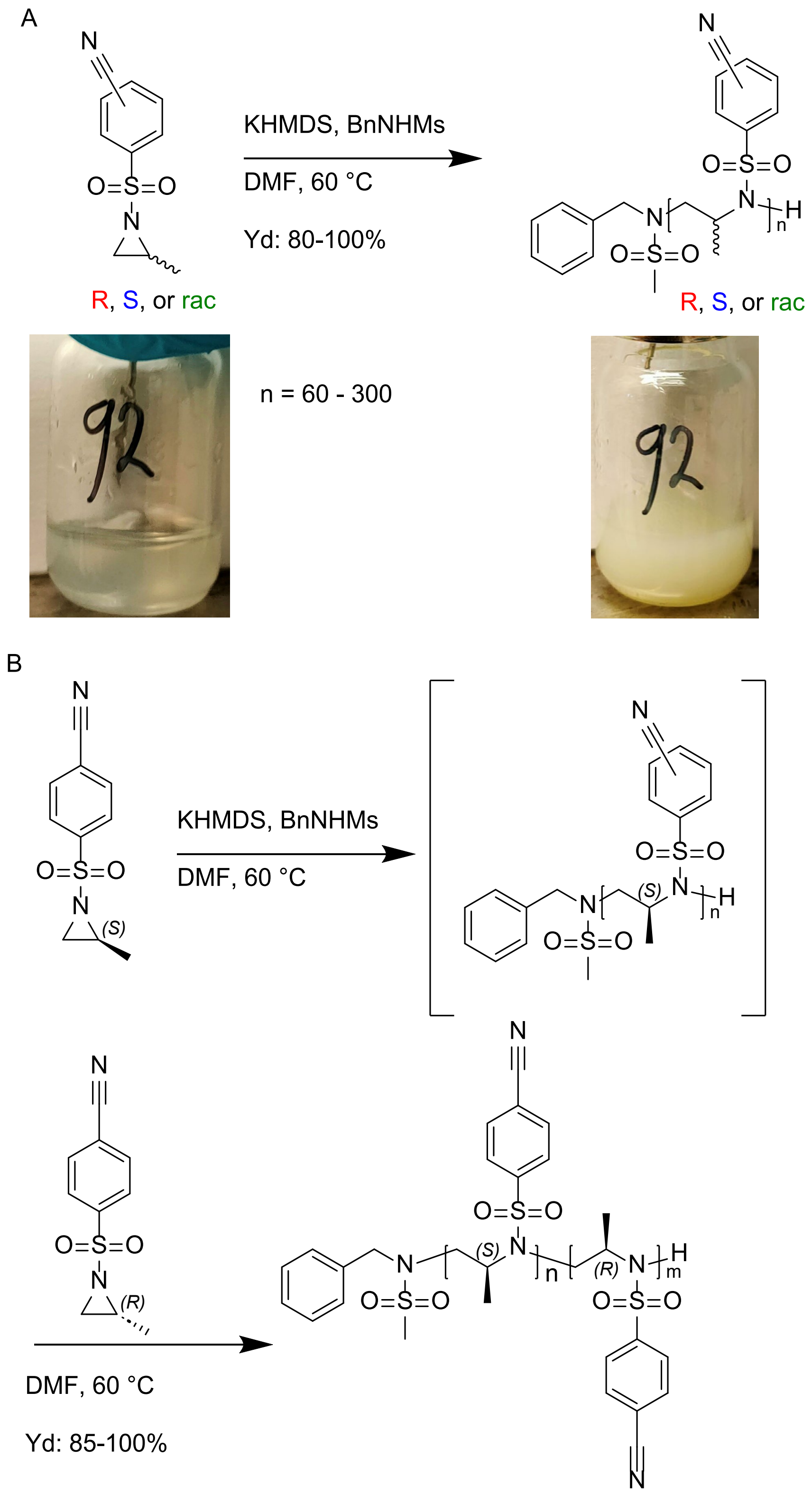

Scheme S3. A: Overview of homopolymerization with illustrations of suspension polymerization. B: Overview of stereo-block copolymerization, an S/R is shown as example.

Table S1. Overview polysulfonamides DP 60 to 300 (400 mg of monomer, unless otherwise indicated).

**Polysulfonamide**	**Yield (mg)**	**Yield (%)**
Rac-*p*-60	417	98
Rac-*o*-60	405	100
Rac-*p*-100	411	99
Rac-*o*-100	425	100

Rac-*p*-150	427	100
Rac-*o*-150	433	100
Rac-*p*-300	423	100
R-*p*-60	422	99
R-*o*-60	402	99
R-*p*-100	373	92
R-*o*-100	375	93
R-*p*-150	399	99
R-*o*-150	409	97
R-*p*-300	399	99
S-*p*-60	422	99
S-*o*-60	416	98
S-*p*-100 (550 mg scale)	567	97
S-*o*-100	379	94
S-*p*-150	383	95
S-*o*-150	366	91
S-*p*-300	411	97
R-*o*-50/S-*o*-50 (block polymer)	341	85
R-*p*-70/S-*p*-30 (block polymer, 150 mg scale)	152	99
R-*p*-30/S-*p*-70 (block polymer, 150 mg scale)	138	96
S-*p*-50/R-*p*-50 (block polymer, 150 mg scale)	127	90
S-*p*-70/R-*p*-30 (block polymer, 150 mg scale)	152	99
S-*p*-30/R-*p*-70 (block polymer, 150 mg scale)	139	96

Appearance: white powder

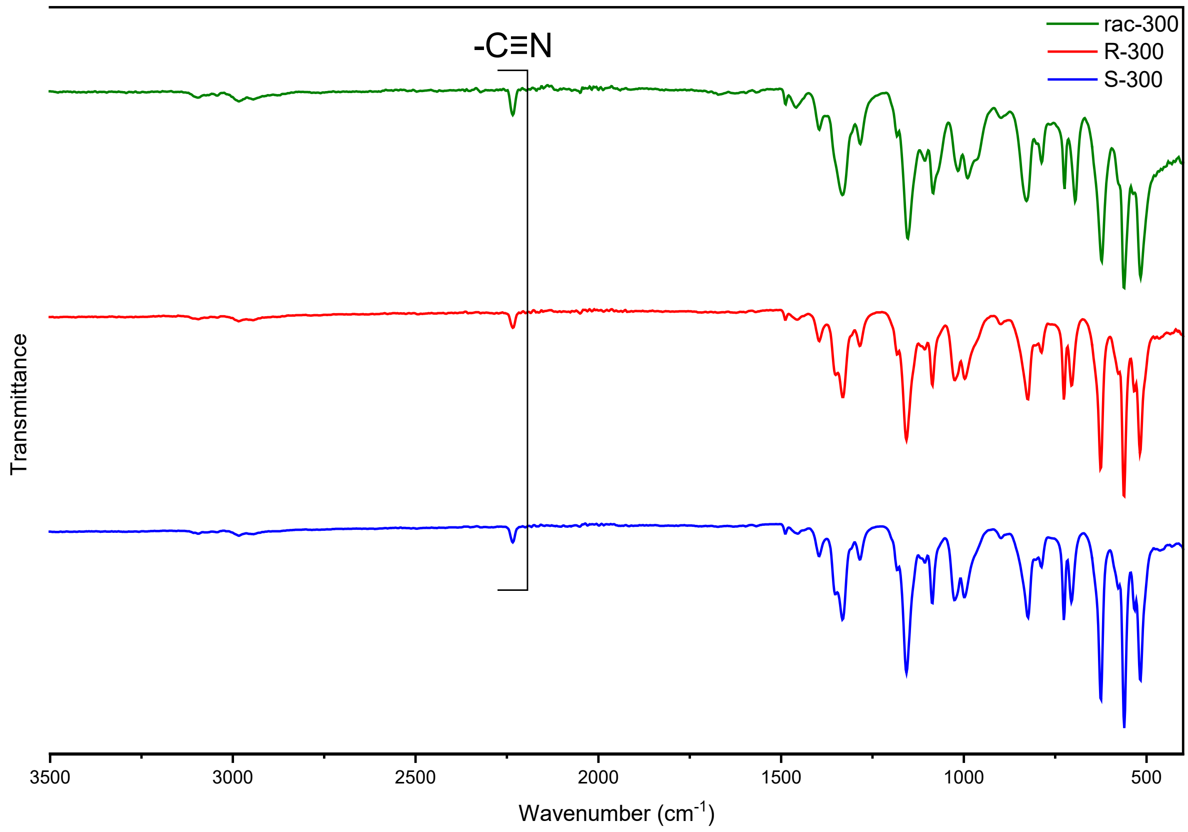

Figure S24. FTIR of polysulfonamides in racemic, R and S configuration with a DP of 300.

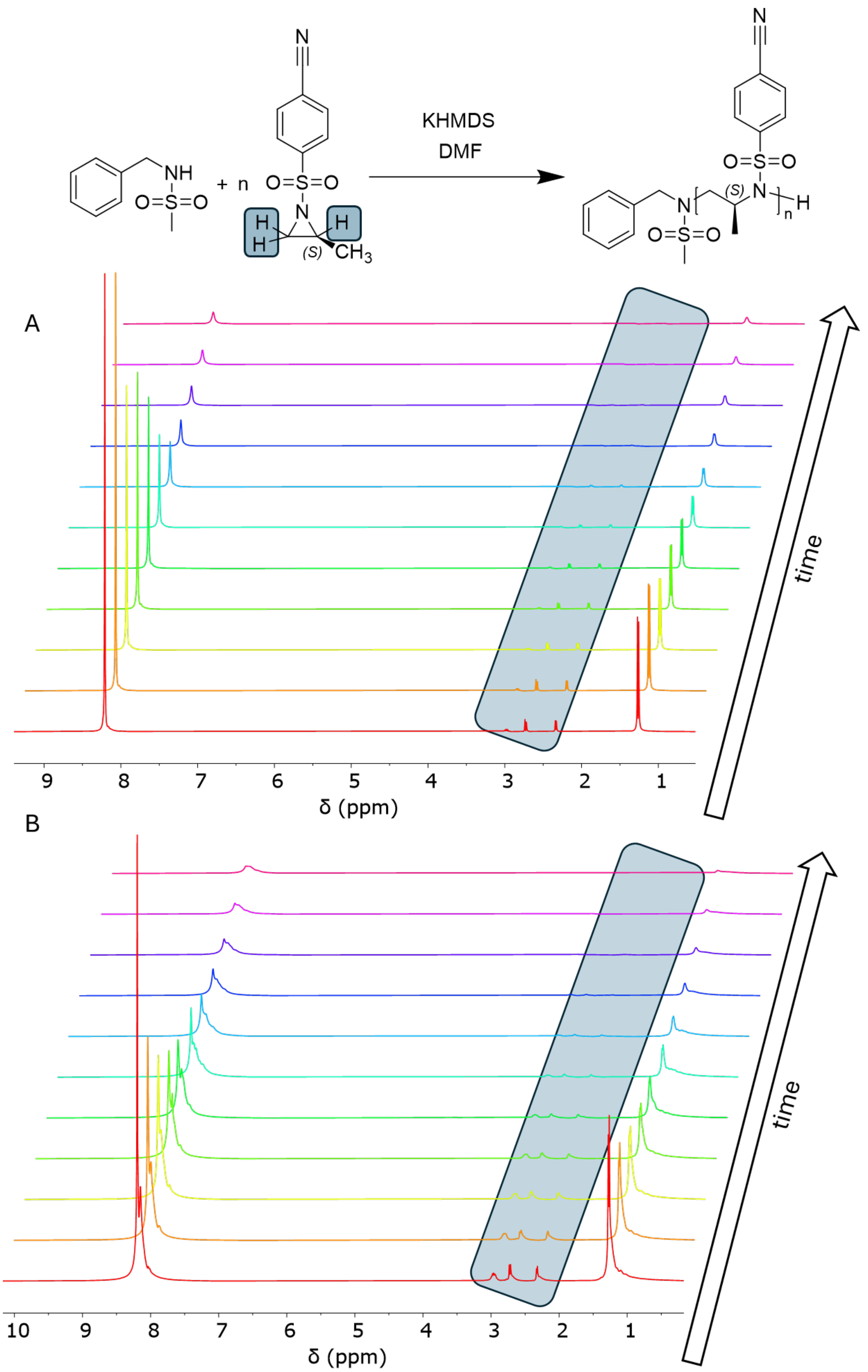

Figure S25. NMR-kinetics showing the consumption of the monomer overtime at A: 40 °C and B: 60 °C. Peaks in the grey box were taken for calculating the kinetics.

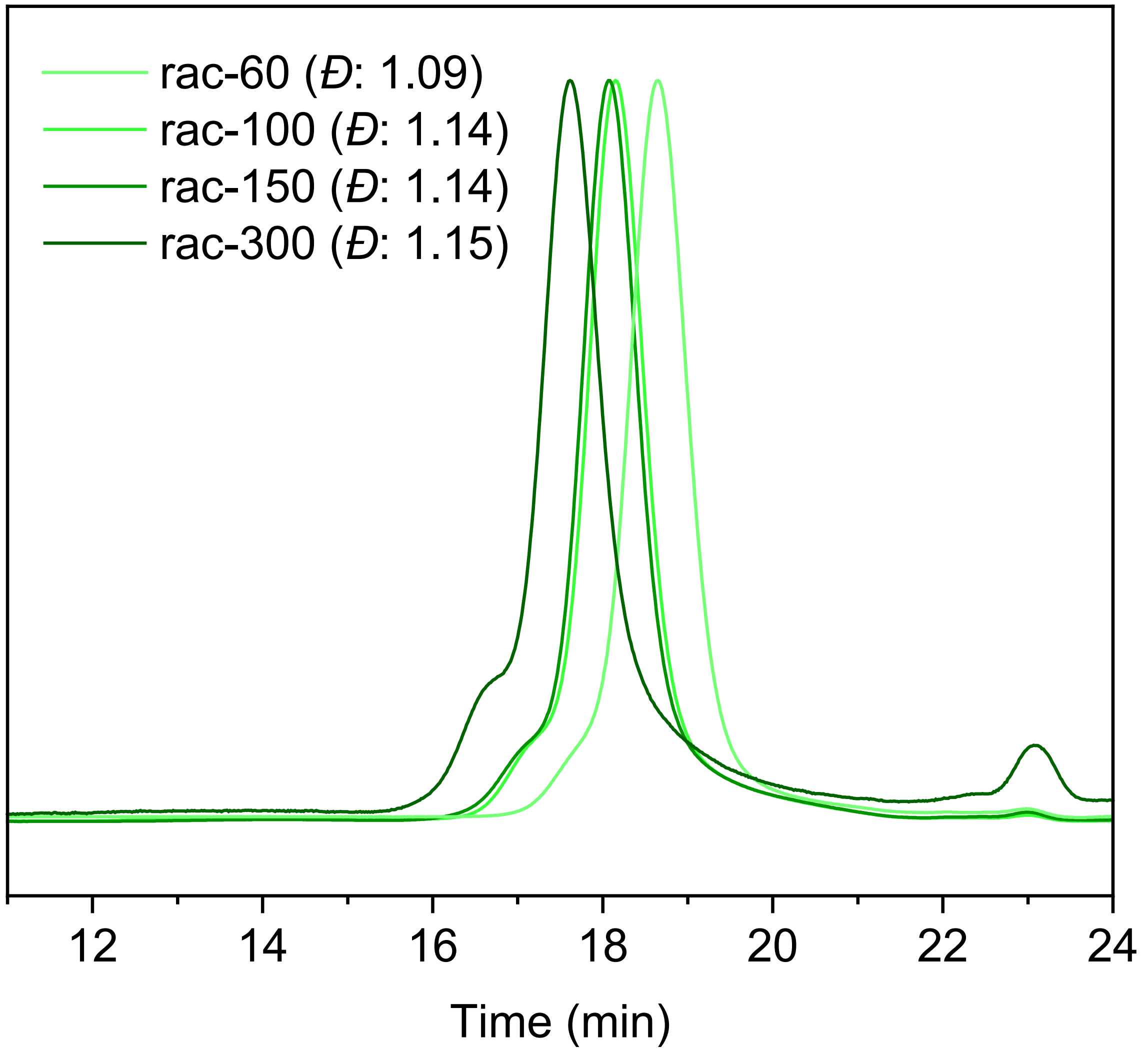

Figure S26. SEC elugrams of atactic poly-*p*-sulfonamides with DP ranging from 60 to 300 (DMF with 0.1 M LiCl, RI detection). Peak at 23 min is solvent residue.

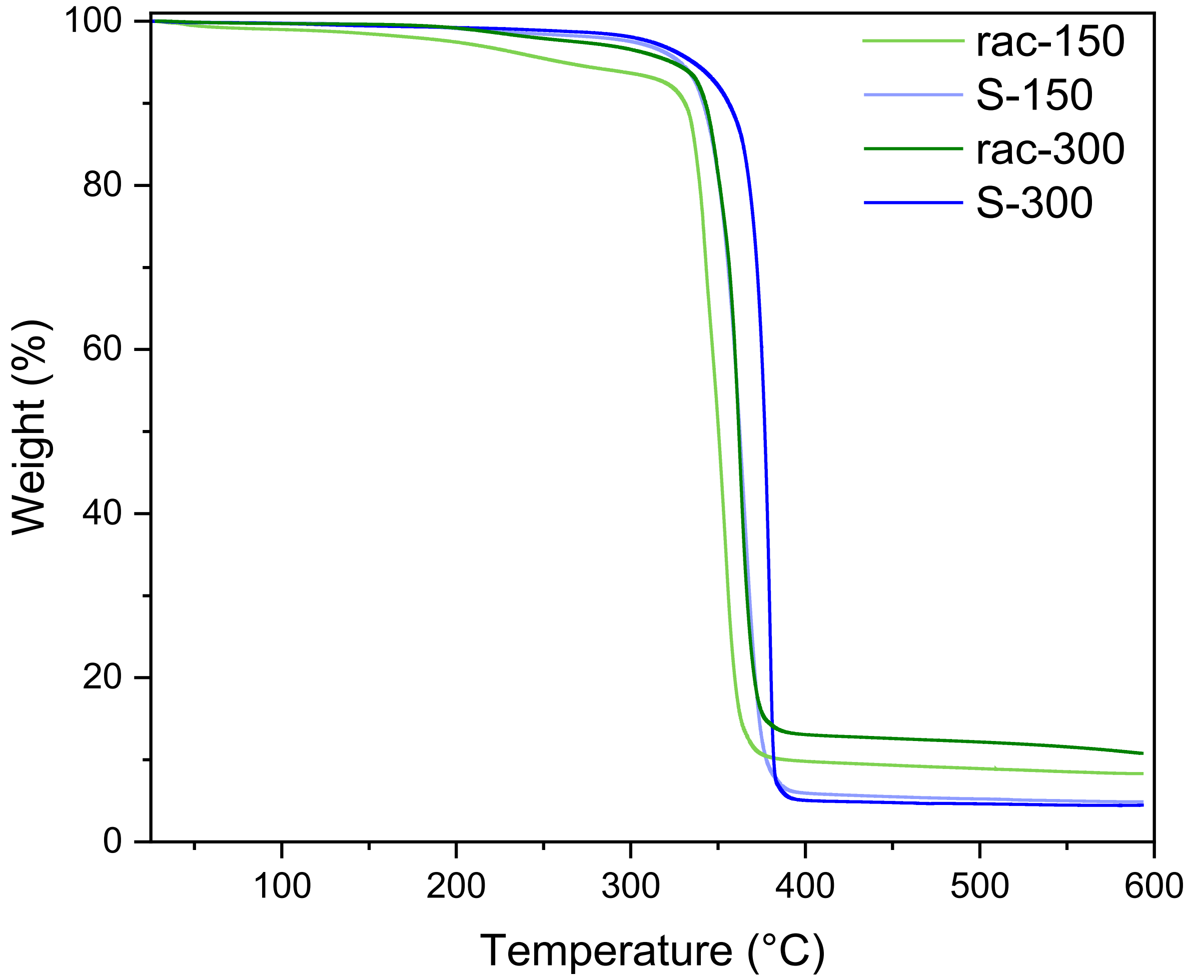

Figure S27. TGA comparison of S,rac-150 and 300 DP polysulfonamides.

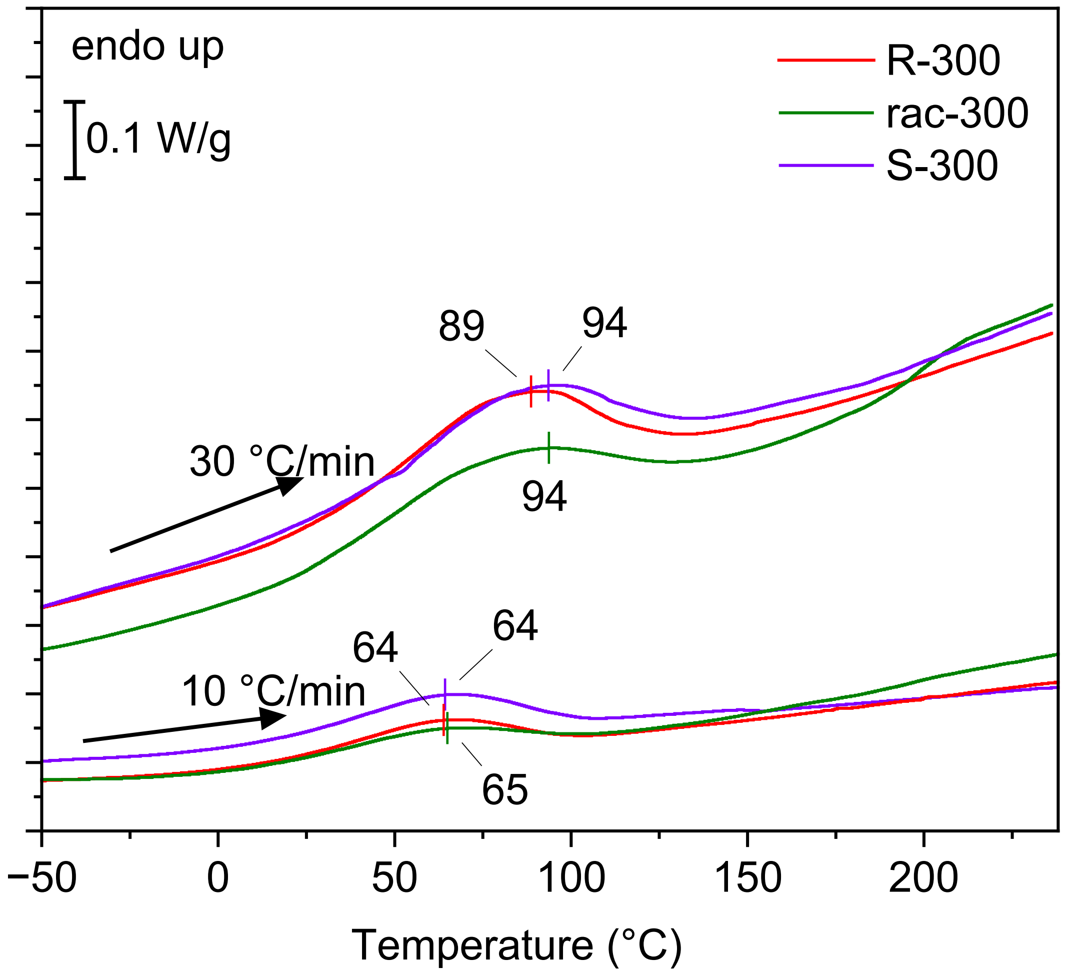

Figure S28. DSC of isotactic R and S polysulfonamide and atactic polysulfonamide with DP 300 (shown is the 1st heating curve with 30 °C/min and 10 °C/min).

Desulfonylation of Polysulfonamides

A vial with a septum screw cap, equipped with magnetic stirrer, was purged with nitrogen. The vial was charged with the polysulfonamide (1 eq corresponding to the sulfonamide unit) in MeCN (1.0 mL), while being purged with nitrogen. Then dodecanthiol (3 eq) and (tert-Butylimino) tris(pyrrolidino)phosphane (1.5 eq) were added and the reaction mixture (white suspension) was left to stir at room temperature overnight. The clear yellow solution was acidified with 2 M HCl (5.0 mL) and stirred for 5 min at room temperature upon which a white suspension was formed. The suspension was concentrated under reduced pressure. The residue was suspended in acetone/methanol (1/1) and the solids were filtered and washed with acetone/methanol (1/1) twice, dried under reduced pressure and lyophilized to afford a white fluffy powder or yellow solids, yields: typically above 95%.

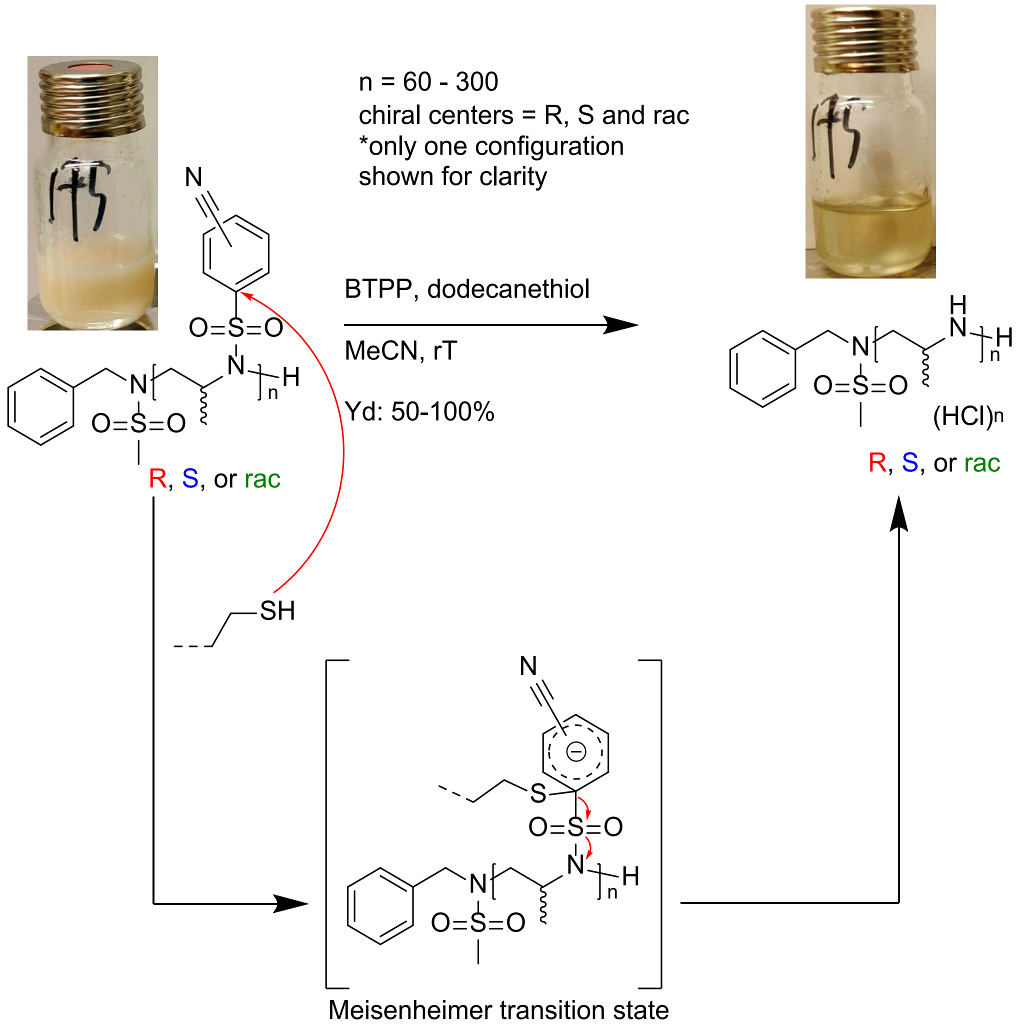

Scheme S4. Overview desulfonylation with illustrations of suspension to clear solution and proposed reaction mechanism through a Meisenheimer complex.

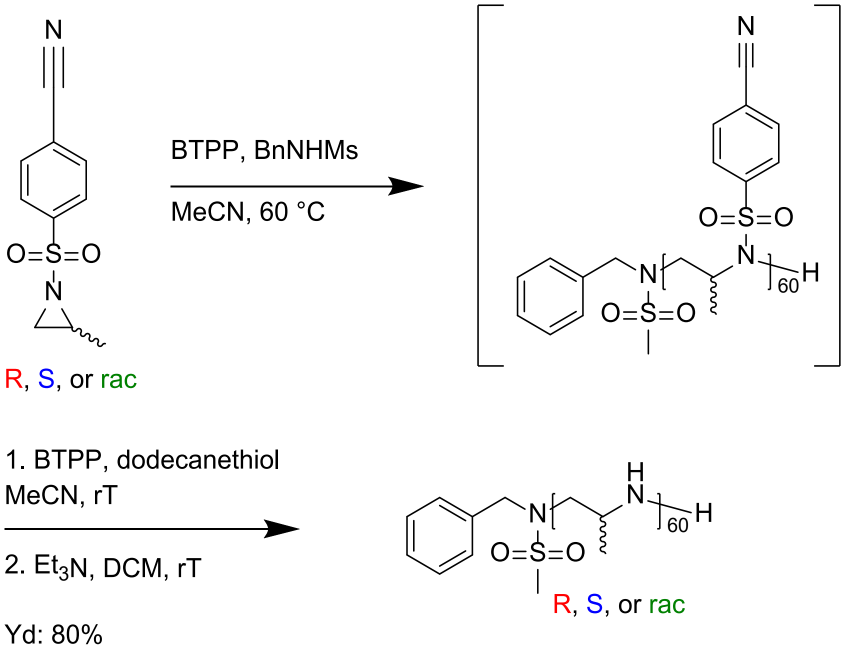

Scheme S5. One-pot polymerization and desulfonylation with S-60.

Table S3. Overview of polyamine HCl salts DP 60 to 300 (100 mg of polysulfonamide).

**Polyamine.HCl**	**Yield (mg)**	**Yield (%)**	**Comments**
Rac-60[a]	23	54	
Rac-100[a]	39	92	
Rac-150[a]	41	97	
Rac-300[a]	42	99	
R-60[b]	31	72	
R-100[b]	43	100	
R-150[b]	42	99	
R-300[b]	44	100	
S-60[a]	43	80	Directly washed free of HCl
S-100[b]	39	97	
S-150[b]	36	100	
S-300[b]	40	94	
R-50/S-50[b] (block polymer)	31	100	
R-70/S-30[b] (block polymer)	37	86	
R-30/S-70[b] (block polymer)	39	91	
S-50/R-50[b] (block polymer)	39	91	
S-70/R-30[b] (block polymer)	36	86	
S-30/R-70[b] (block polymer)	39	91	

Appearance: yellow solid[a] or white fluffy powder[b]

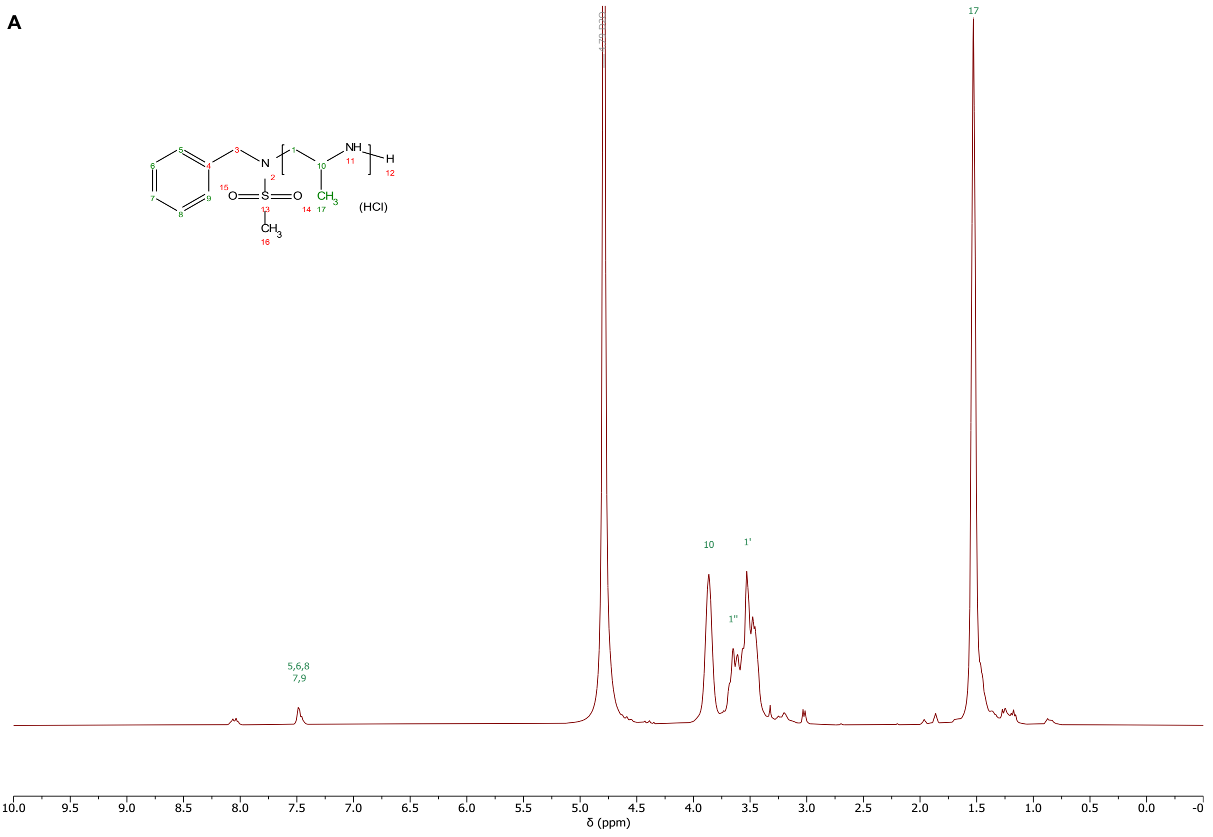

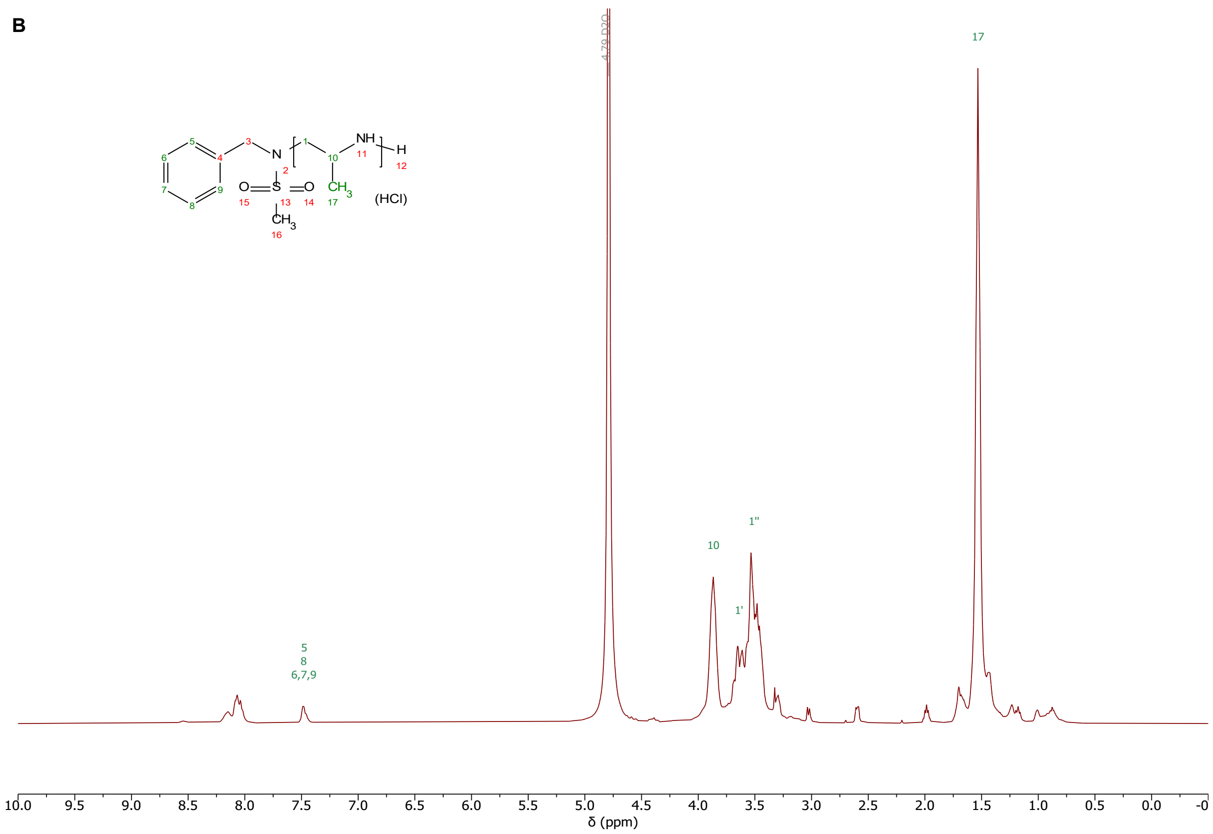

Figure S29. ^{1}H NMR after desulfonylation and purification with A: BTPP as base and B: DBU as base using a racemic DP 60 polysulfonamide (400 MHz, D_2O).

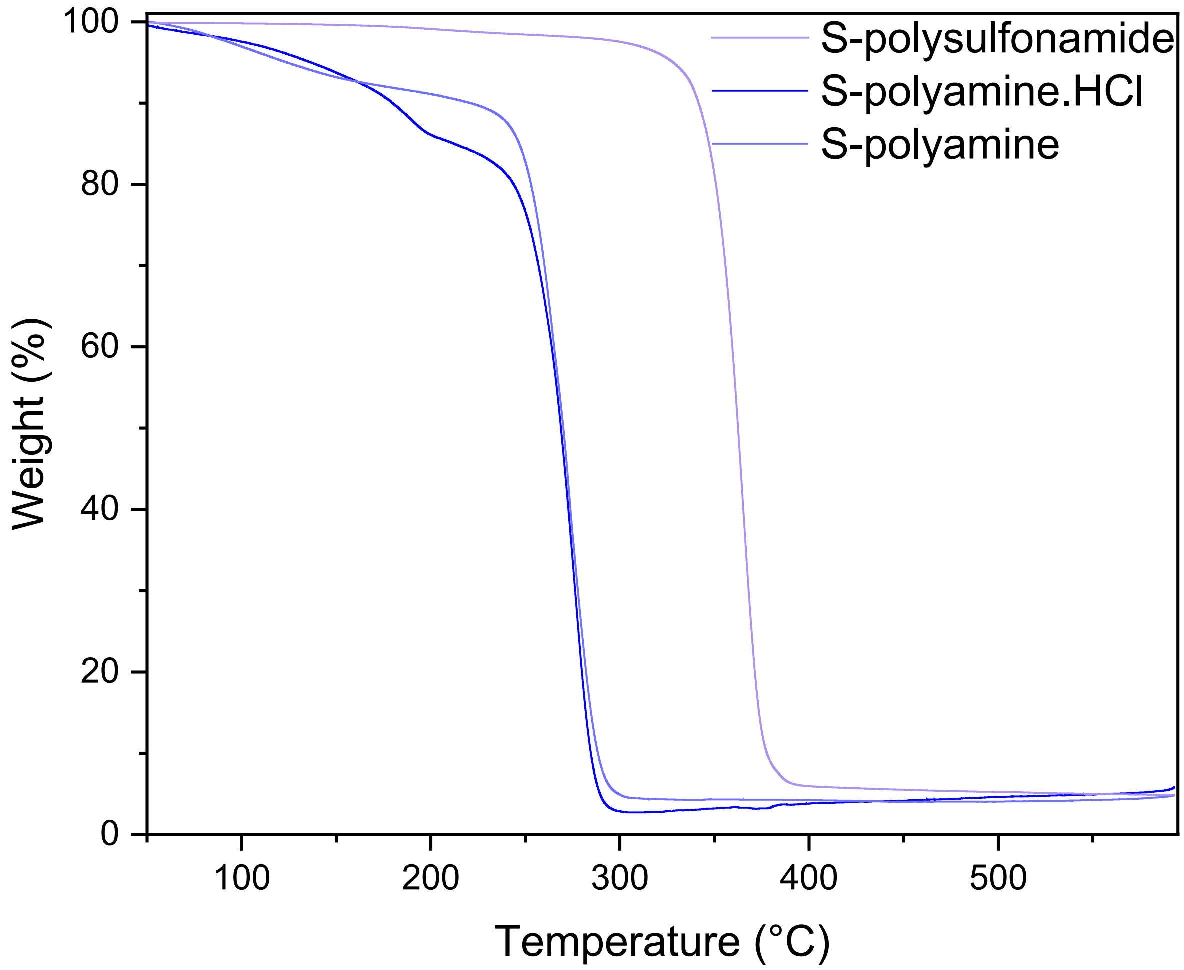

Figure S30. Thermal stability of S-150 polysulfonamide vs polyamine (salt and free base) measured by TGA (10 °C/min, under N_2 atmosphere).

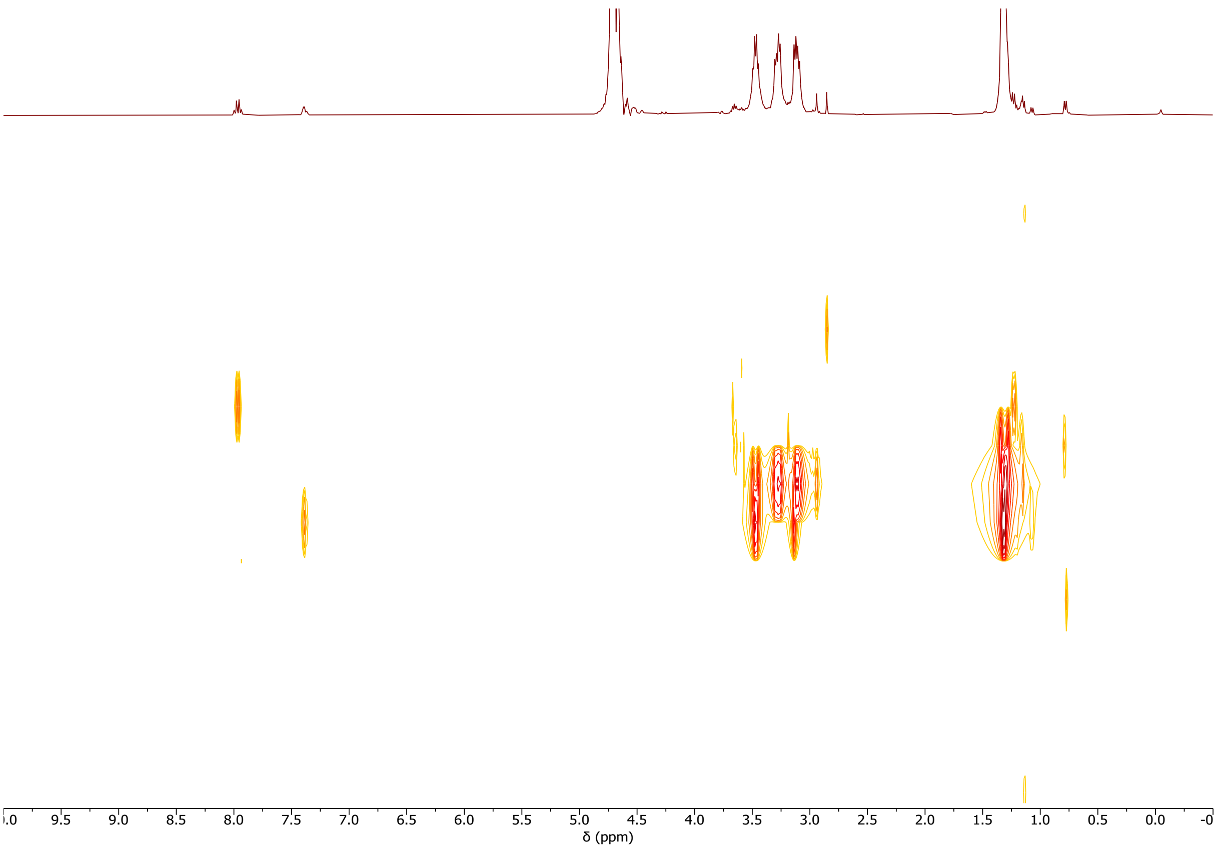

Figure S31. DOSY NMR of block copolymer S-30/R-70 (400 MHz, D_2O).

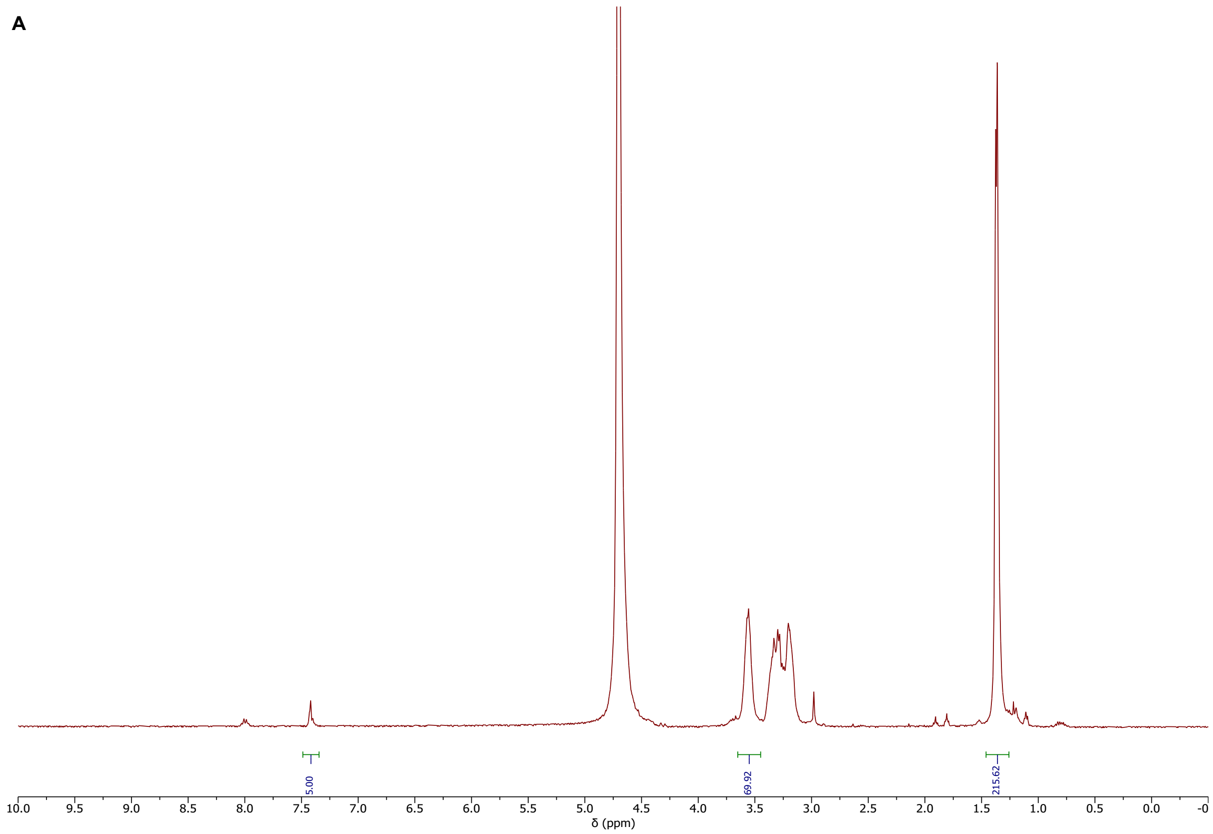

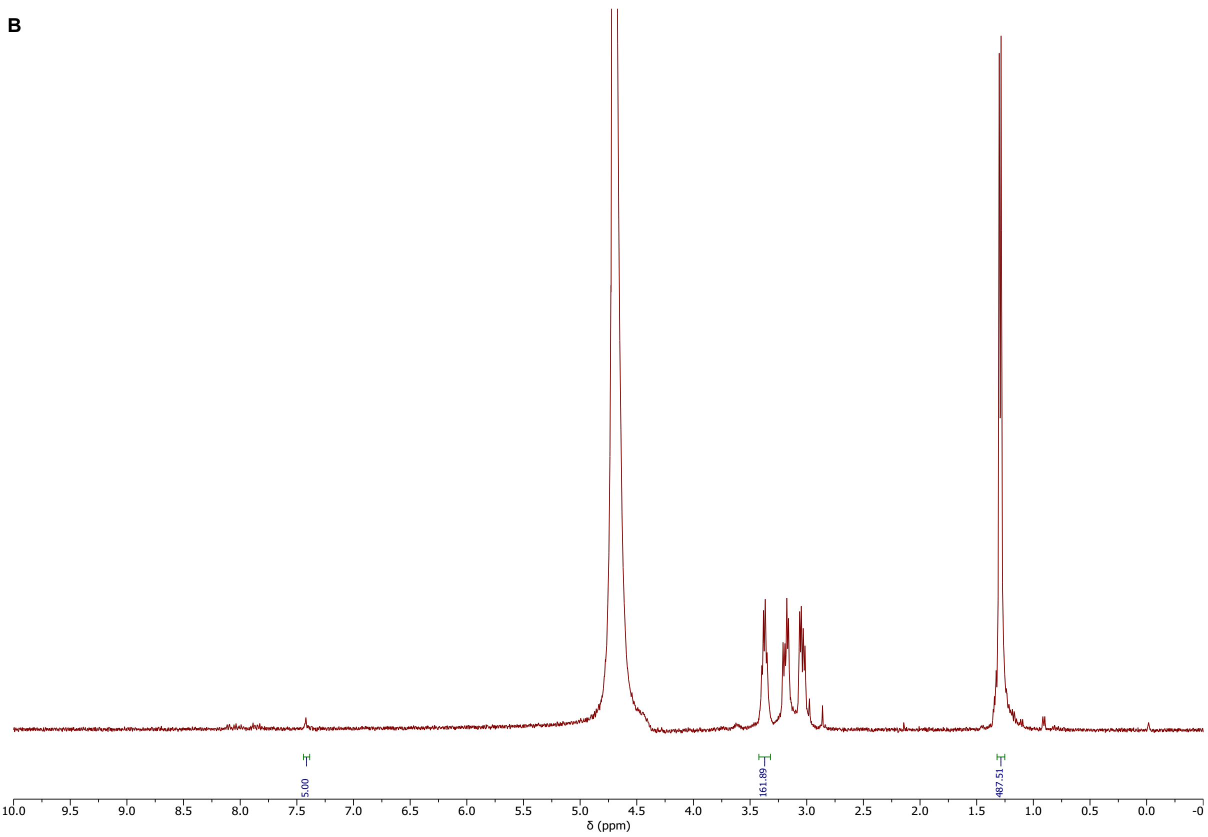

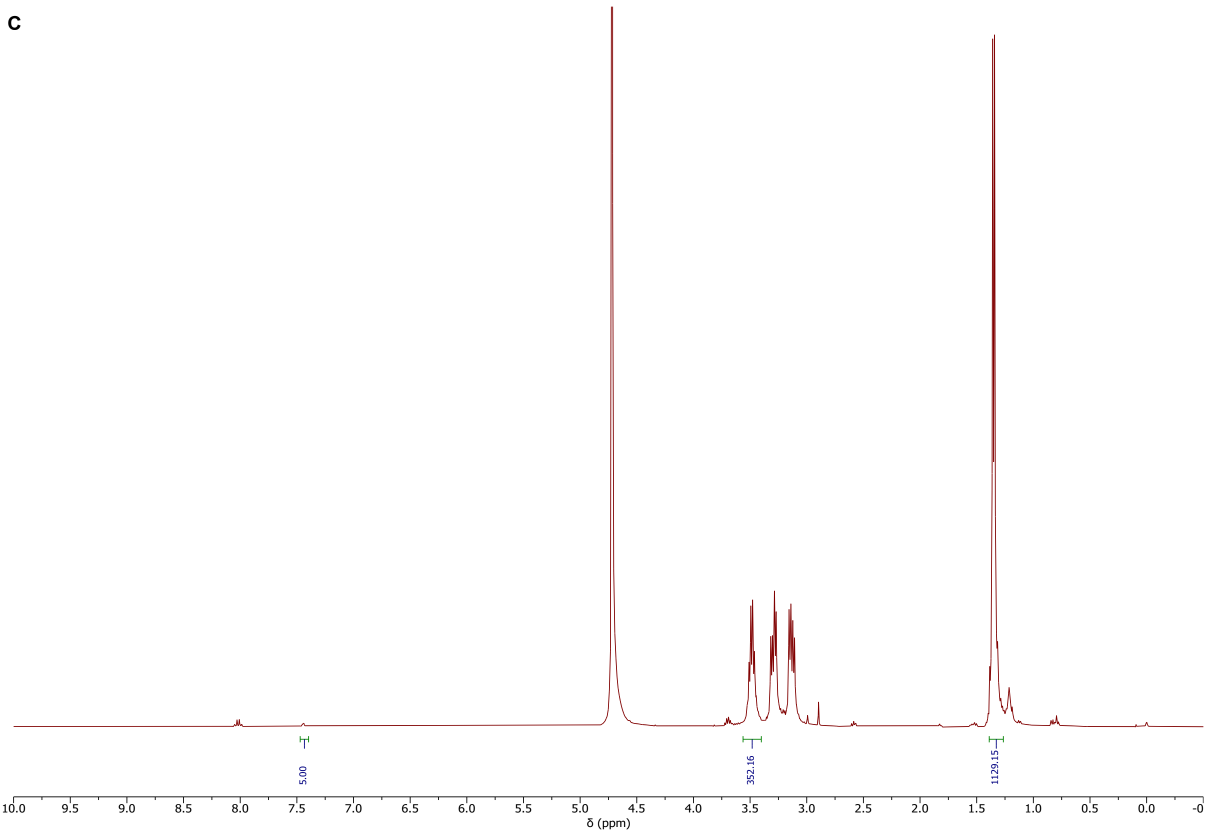

Figure S32. NMR with end-group integration of A: racemic with DP 60, B: R-150 and C: S-300.

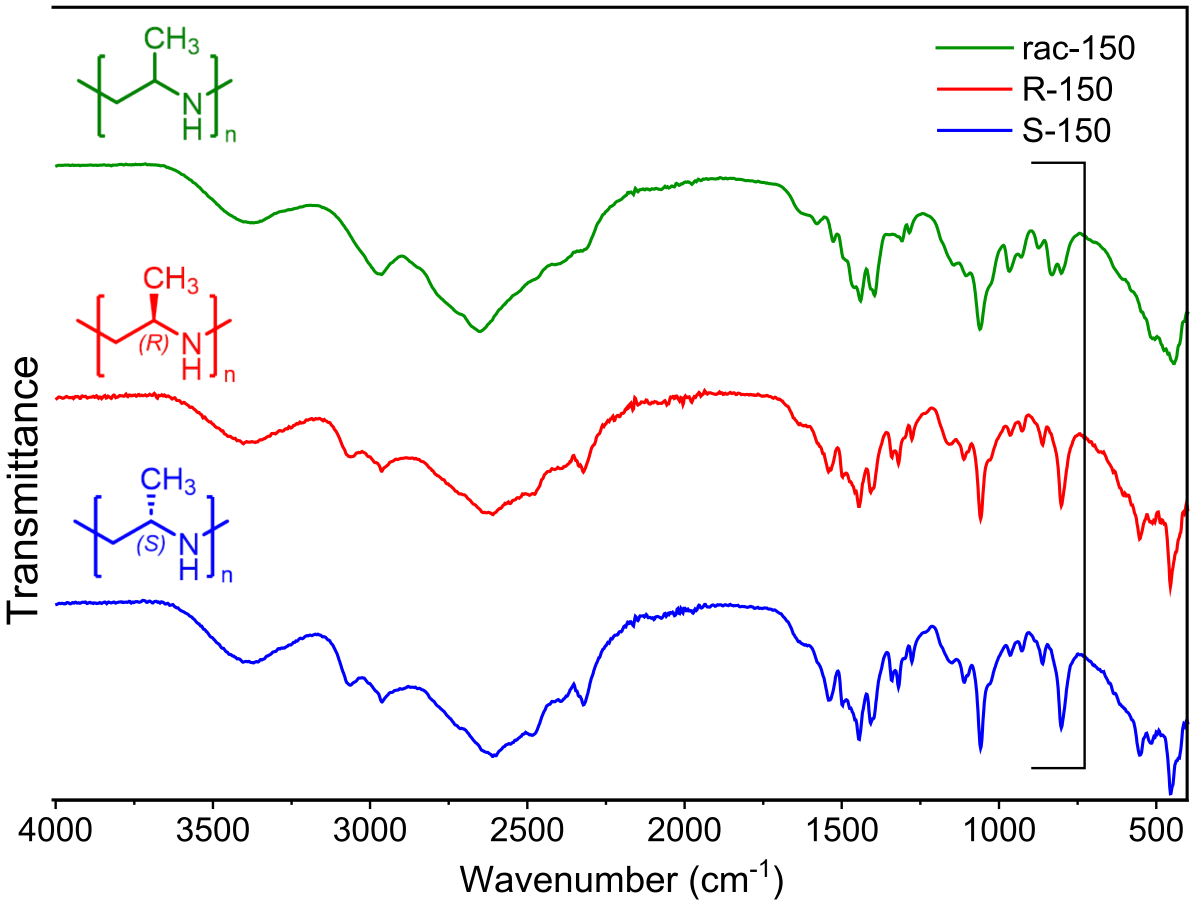

Figure S33. FTIR of DP 150 polyamine.HCl in rac, R and S and indicating the differences between atactic and isotactic polyamine regions.

A

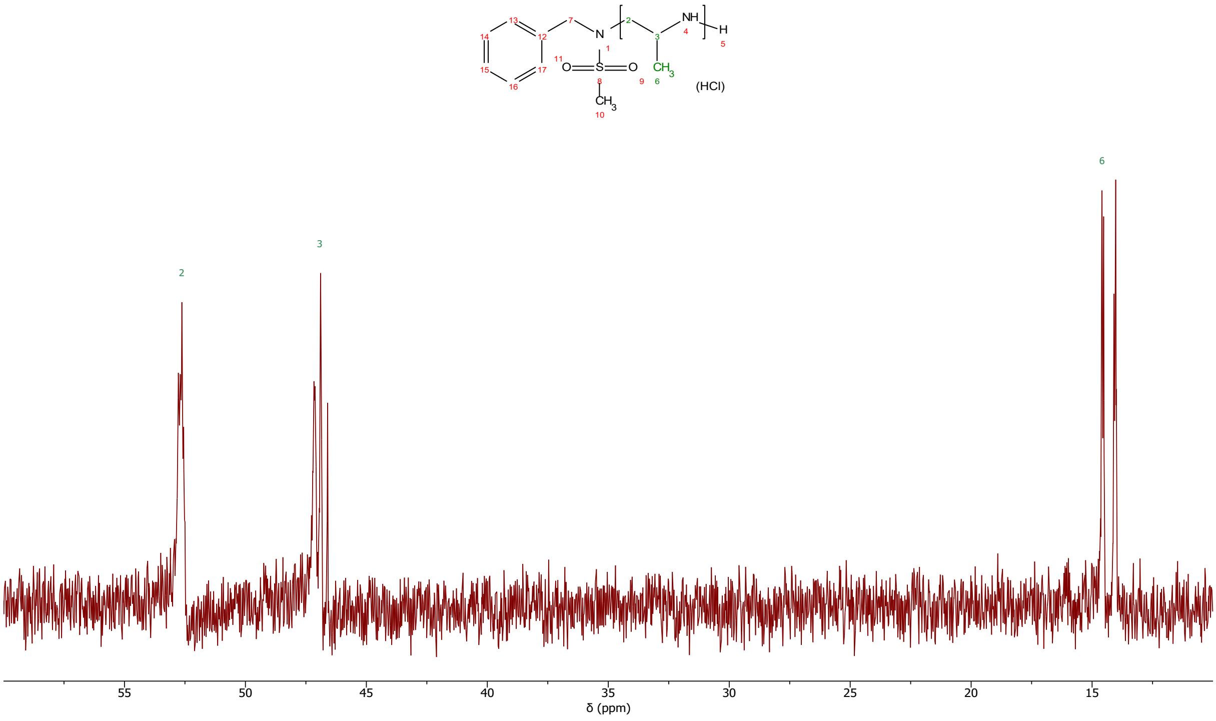

B

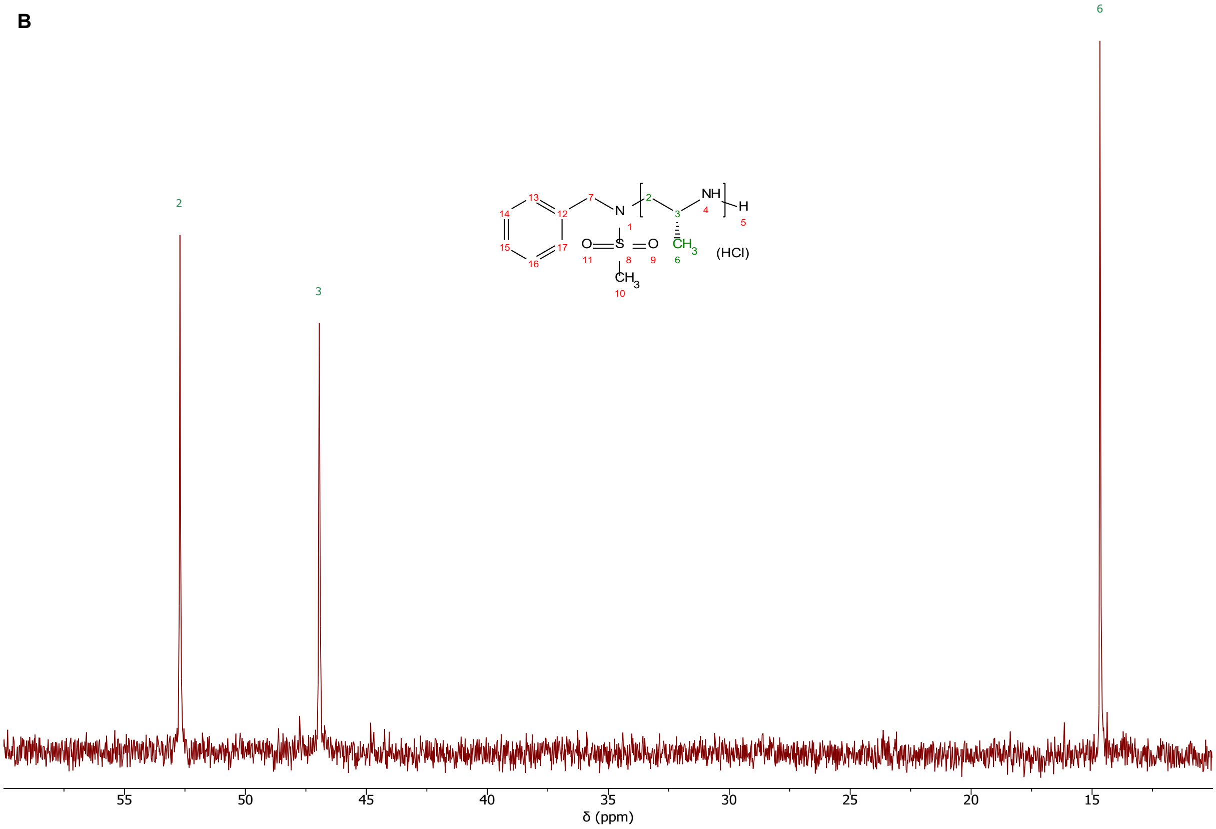

6
2
3
(HCl)
55
50
45
40
35
30
25
20
15
δ (ppm)

C

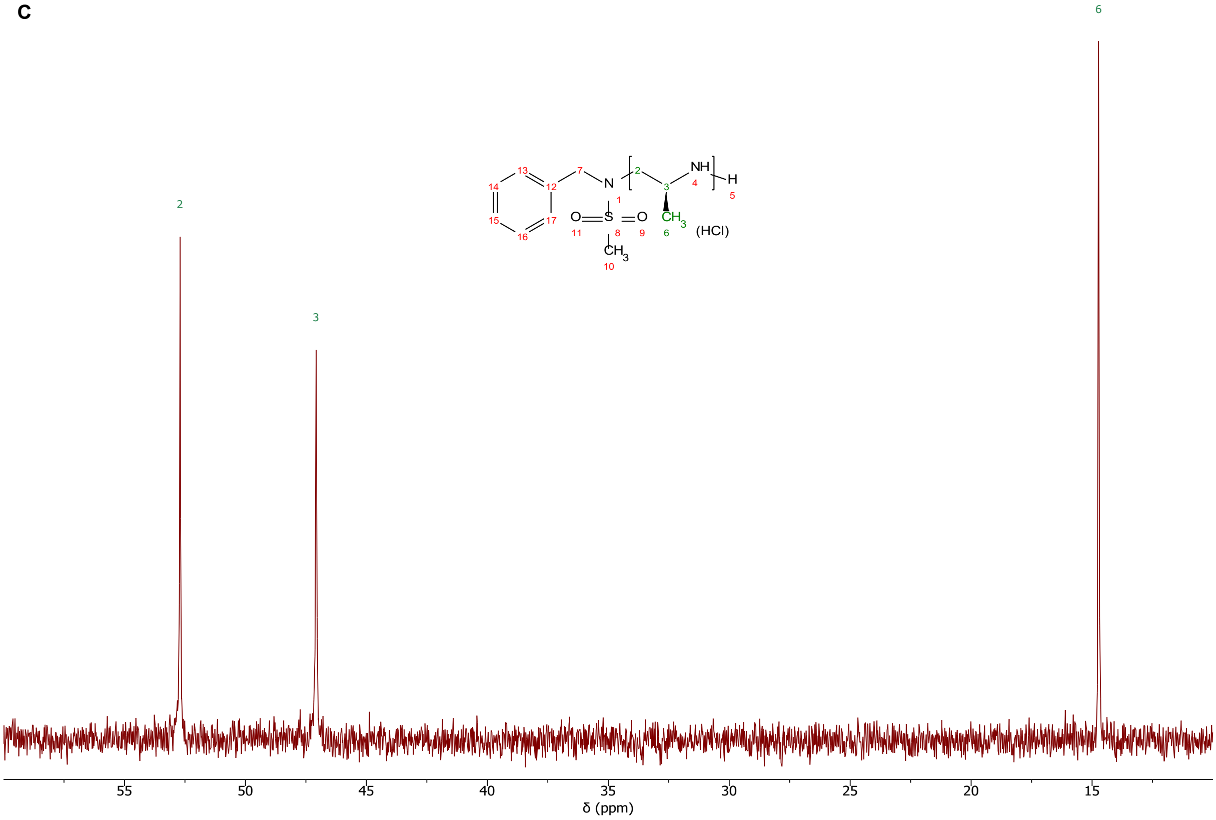

6
2
3
(HCl)
55
50
45
40
35
30
25
20
15
δ (ppm)

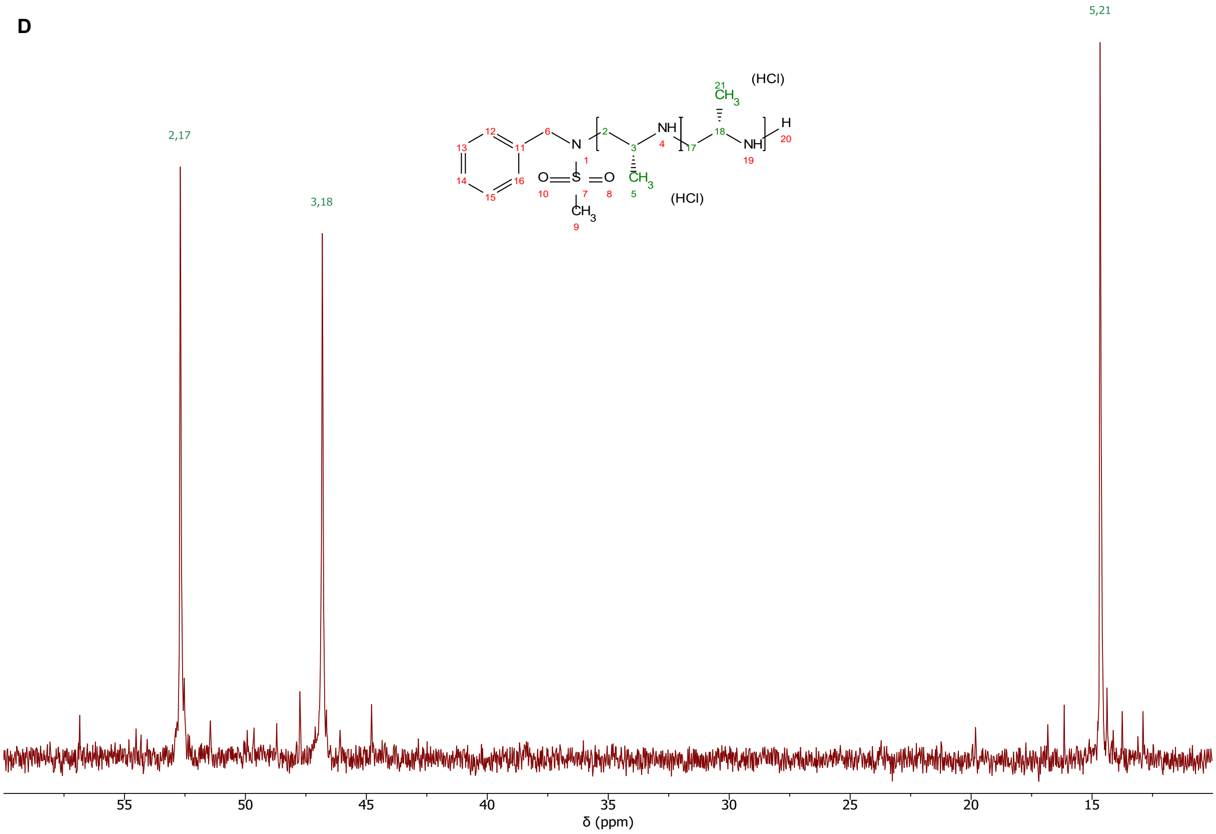

Figure S34. ^{13}C NMR of A: atactic rac-300, B: isotactic R-300, C: isotactic S-150 and D: block copolymer R-30/S-70 (100 MHz, D_2O).

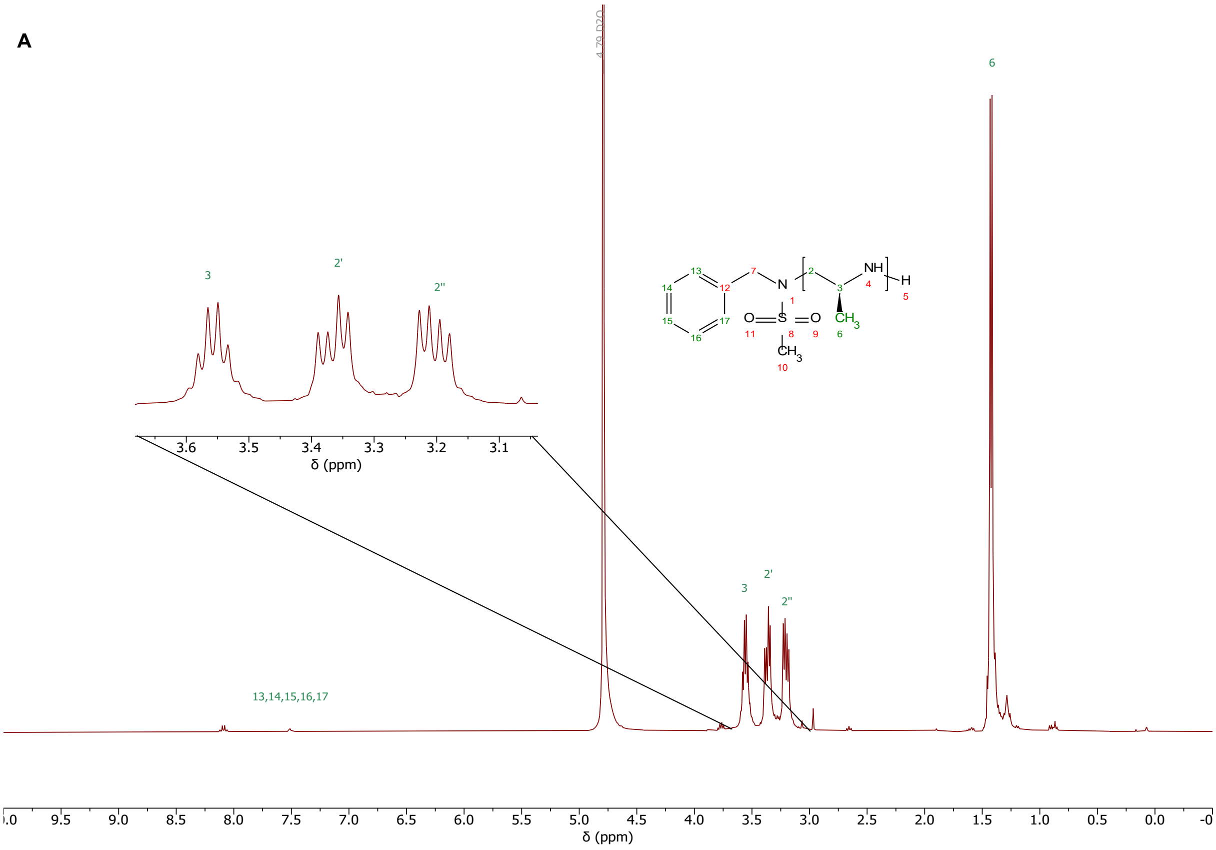

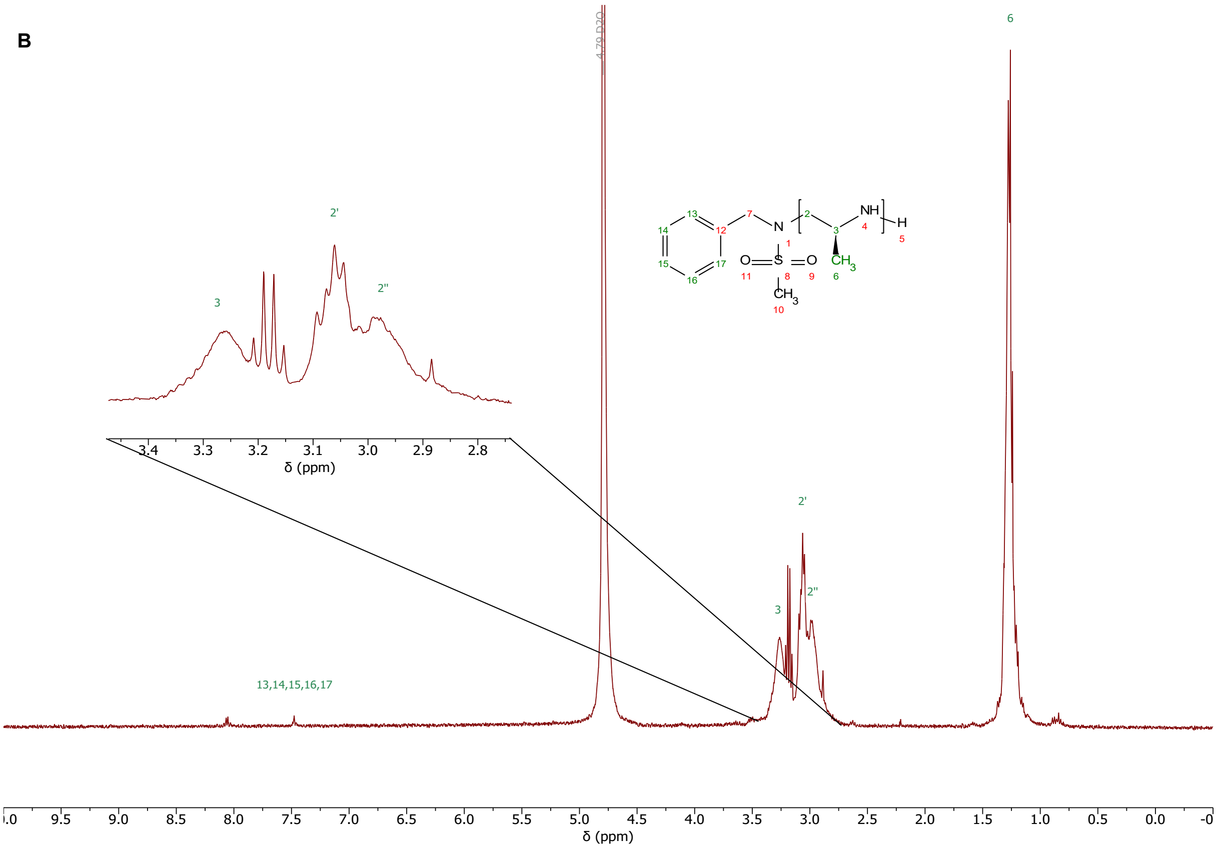

Figure S35. ^{1}H NMR of isotactic polyamine (S-300) as HCl salt (A) and as free base (B) (400 MHz, D_2O).

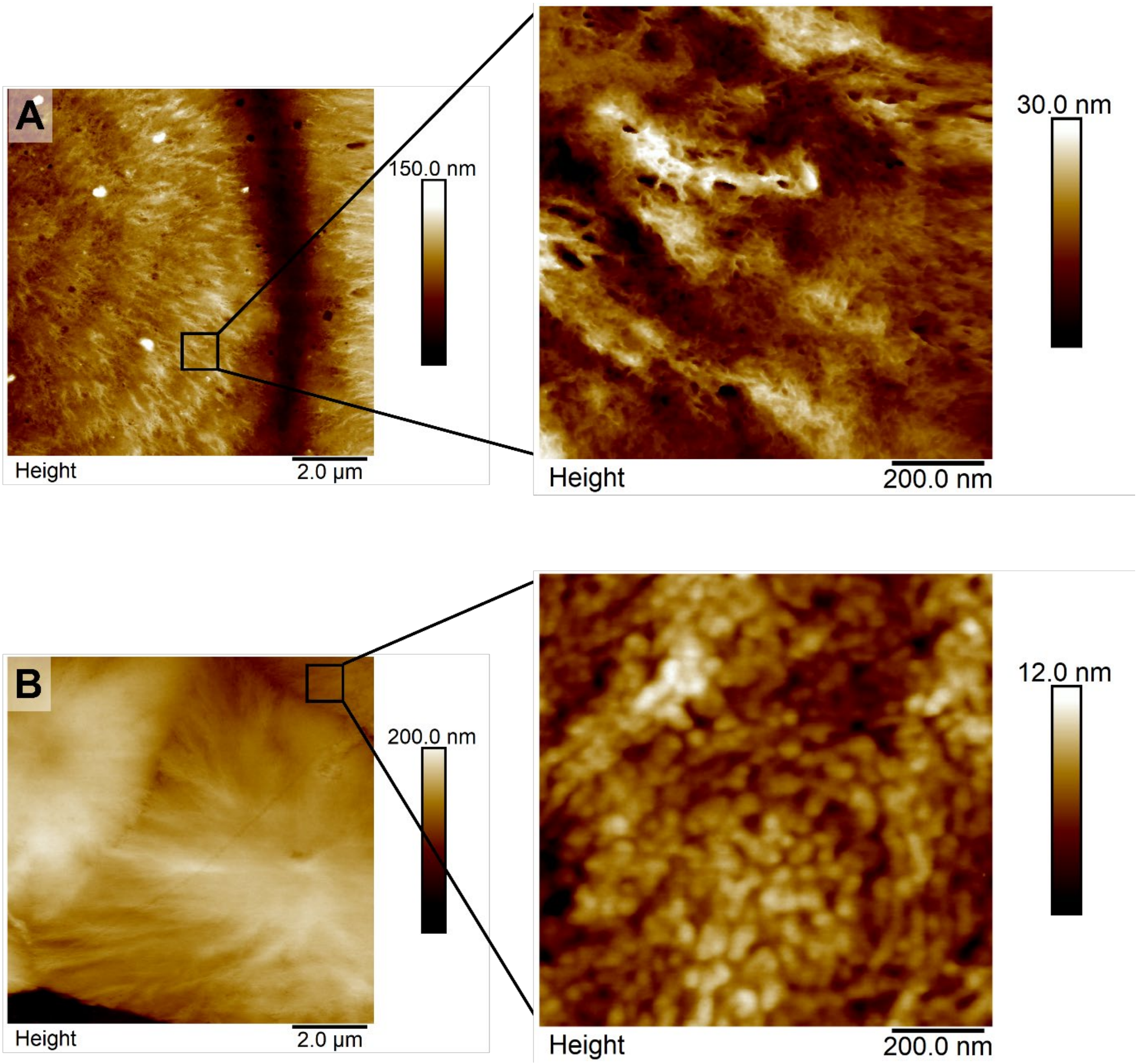

Figure S36. AFM height images of A: R polymer with DP of 300 and B: block copolymer with R50/S50. All block copolymers are structured with initiator:A:B. Squares indicate the zoom-ins.

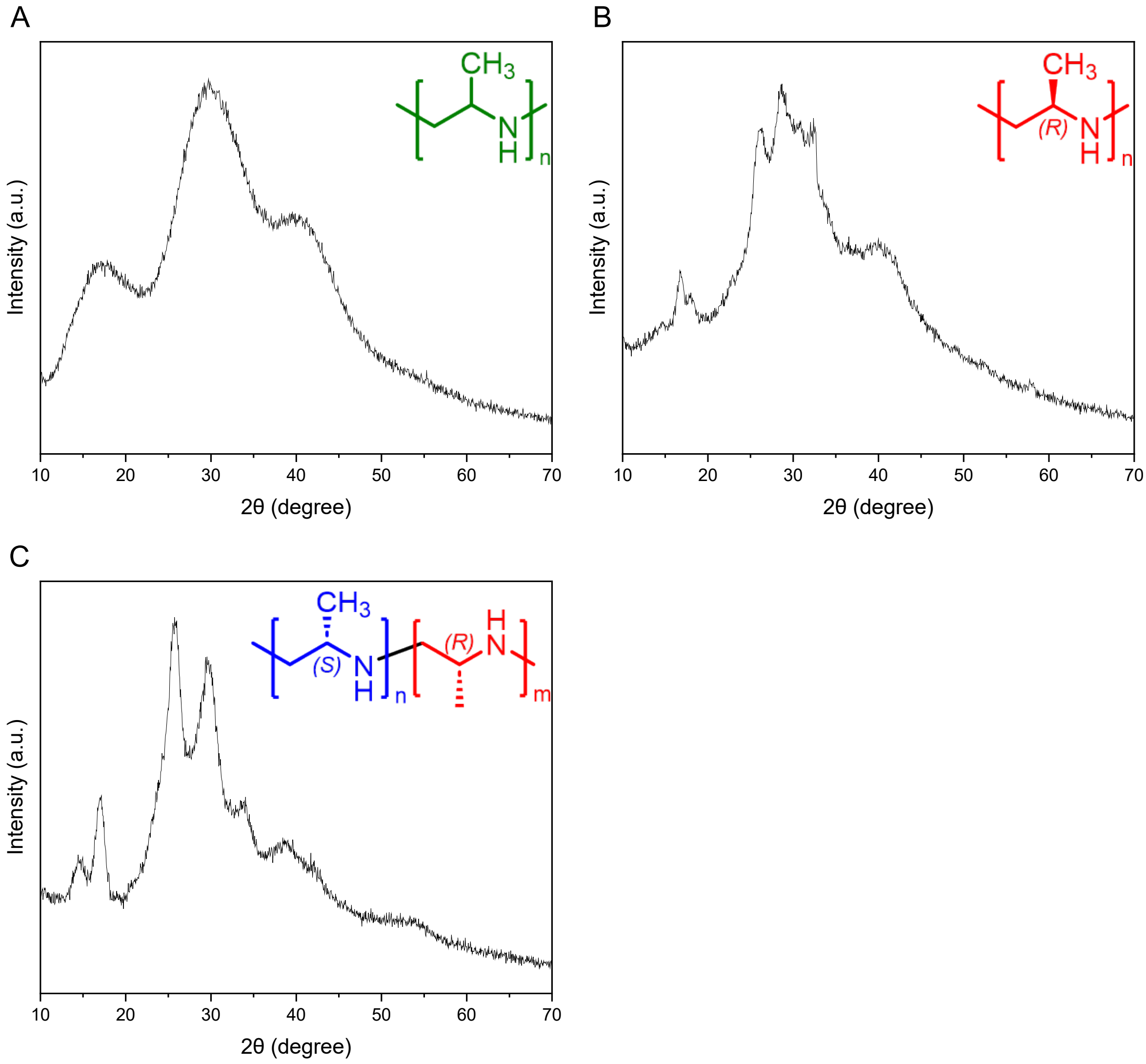

Figure S37. XRD of A: racemic polymer with DP of 300 B: R homopolymer with DP of 300 and C: block copolymer with S70/R30. All block copolymers are structured with initiator:A:B.

Supramolecular Assembly

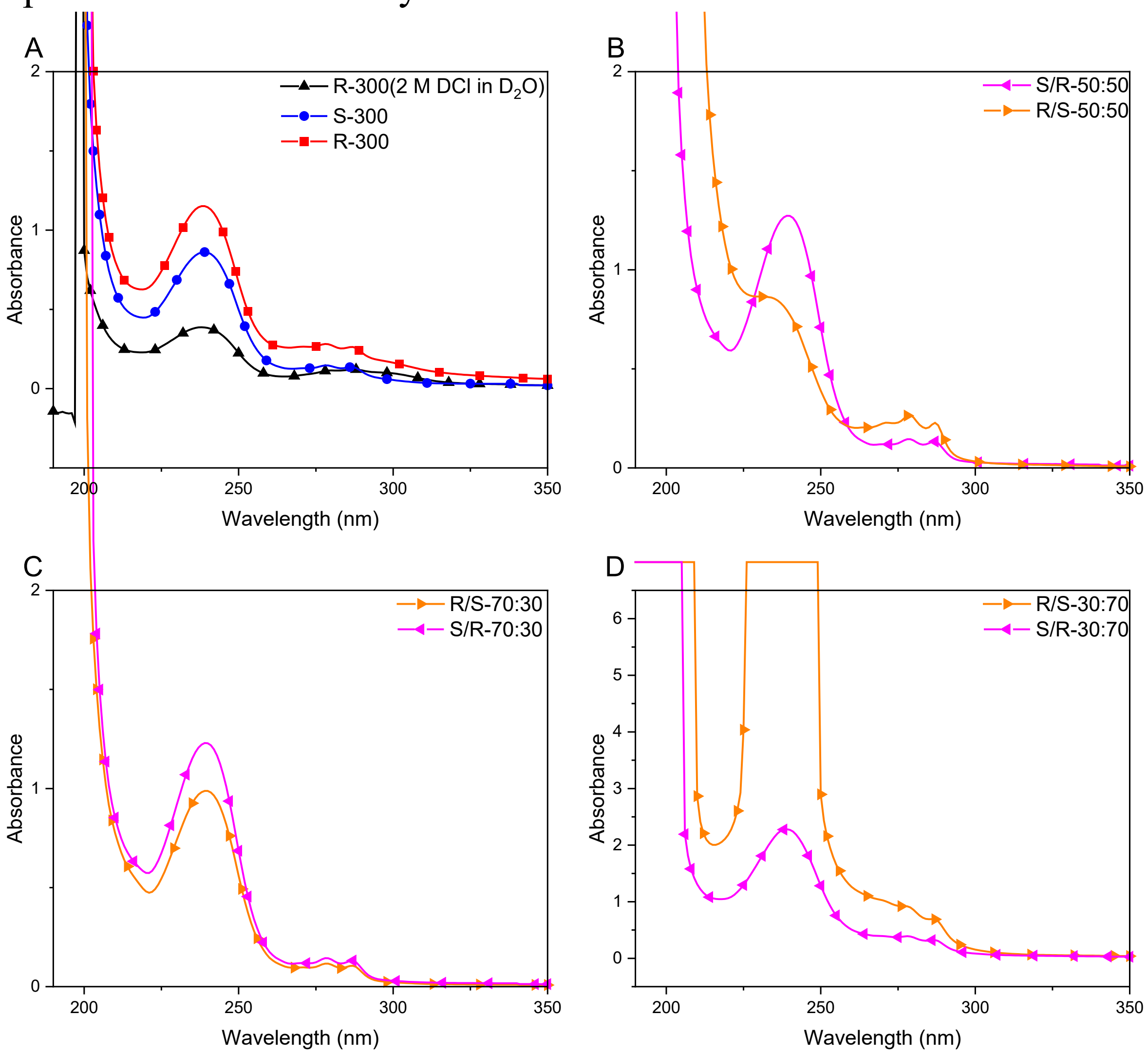

Figure S38. Comparison of different homo- and diblock (co-)polymers with UV-Vis, concentration: 5 mg/mL in demineralized water as HCl salt. A: R and S homopolymer with DP of 300 and R-300 in 2M DCl in D_2O. B: Block copolymer with equal parts. C: Block copolymer with 70/30. D: Block copolymer with 30/70. All block copolymers are structured with initiator:A:B.

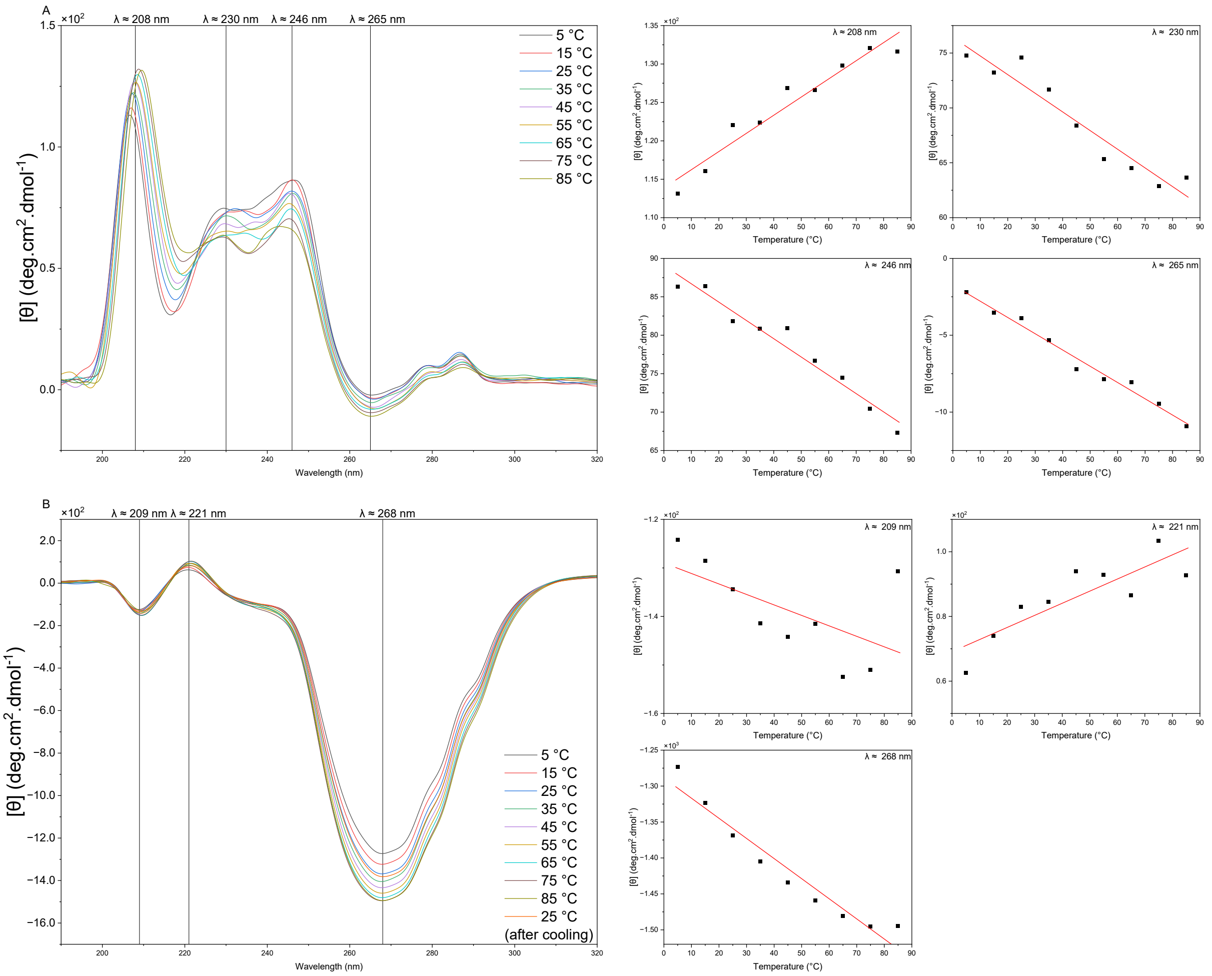

Figure S39. CD spectra of temperature influence on the secondary structure for A: R-300 with a concentration of 10 mg/mL in demineralized water and B: R/S-30/70 with a concentration of 5 mg/mL in demineralized water. Left side shows the whole wavelength range and the right side shows multiple CD bands from peaks. All block copolymers are structured with initiator:A/B. The Y-axis was calculated in mean residue ellipticity as described by Kelly *et.al.*[11]

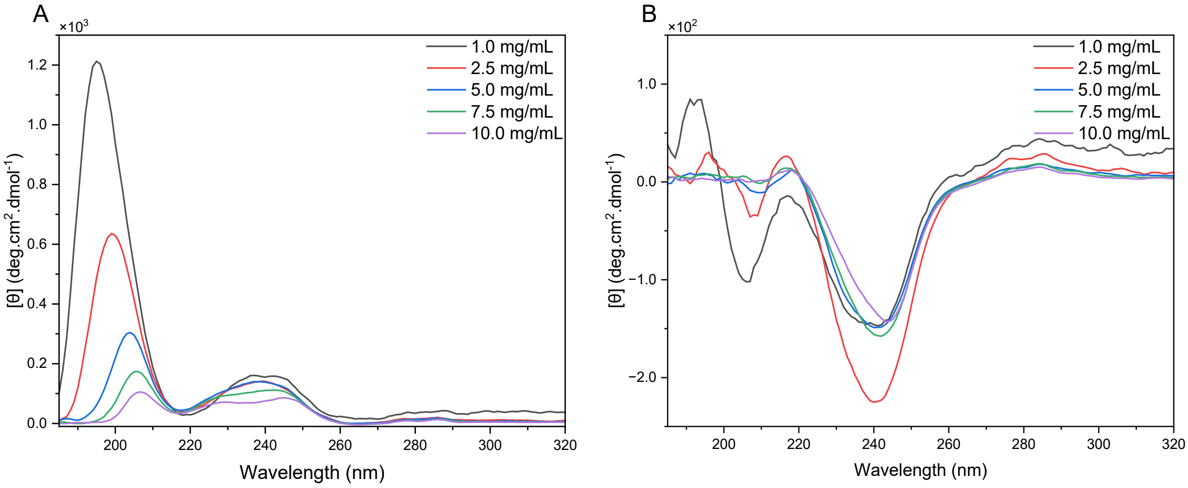

Figure S40. Comparison of different concentrations with CD spectroscopy. A: R-300 homopolymer B: R/S-50/50 block copolymer. All block copolymers are structured with initiator:A:B. The Y-axis was calculated in mean residue ellipticity as described by Kelly *et.al.*[11]

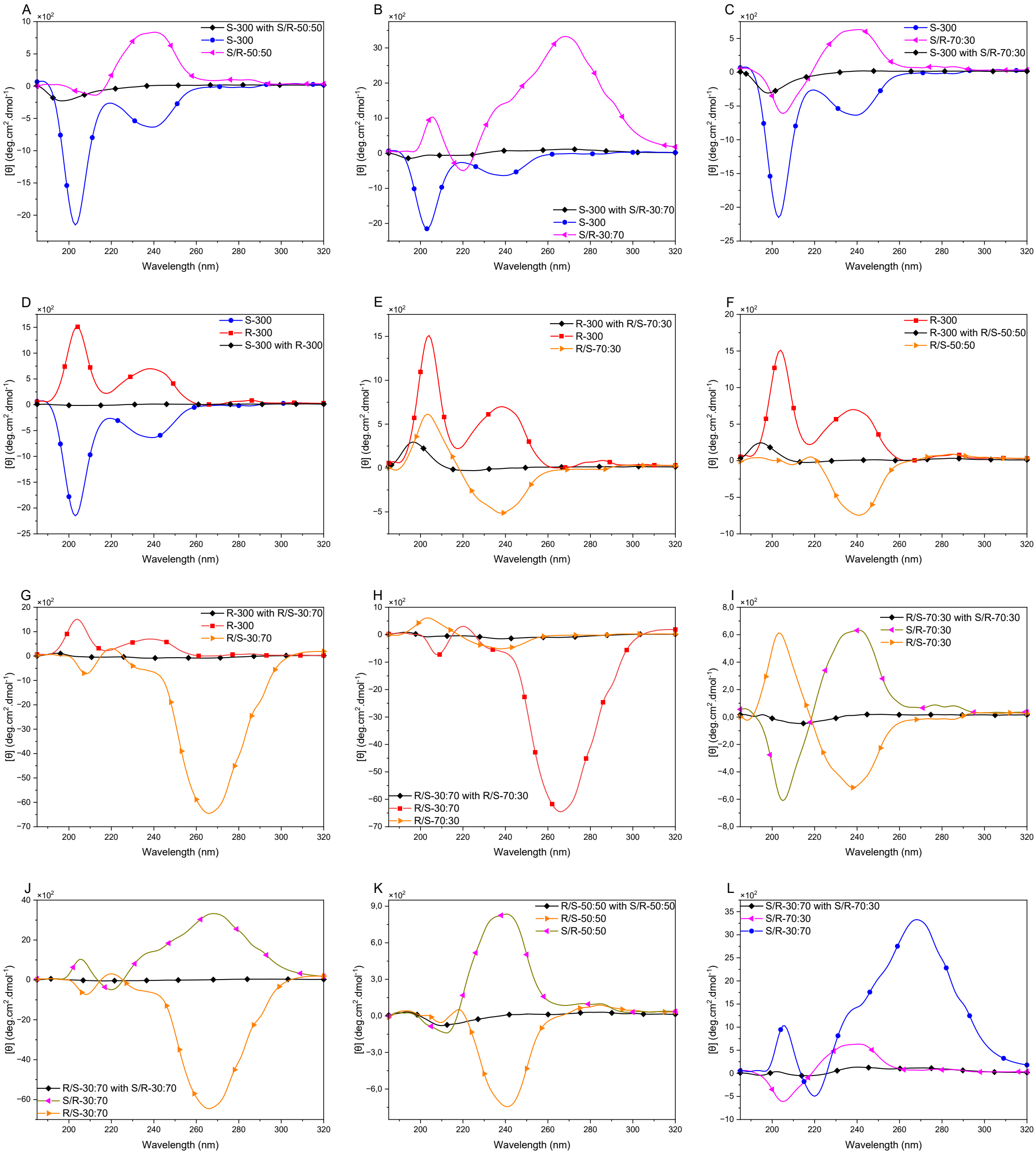

Figure S41. Comparison of different mixtures of homo- and stereo-block (co-)polymers with CD spectroscopy, concentration: 1 mg/mL in demineralized water as HCl salt. All mixtures were in an 1:1 ratio. All block copolymers are structured with initiator:A:B. The Y-axis was calculated in mean residue ellipticity as described by Kelly *et.al.*[11]

Molecular Dynamics

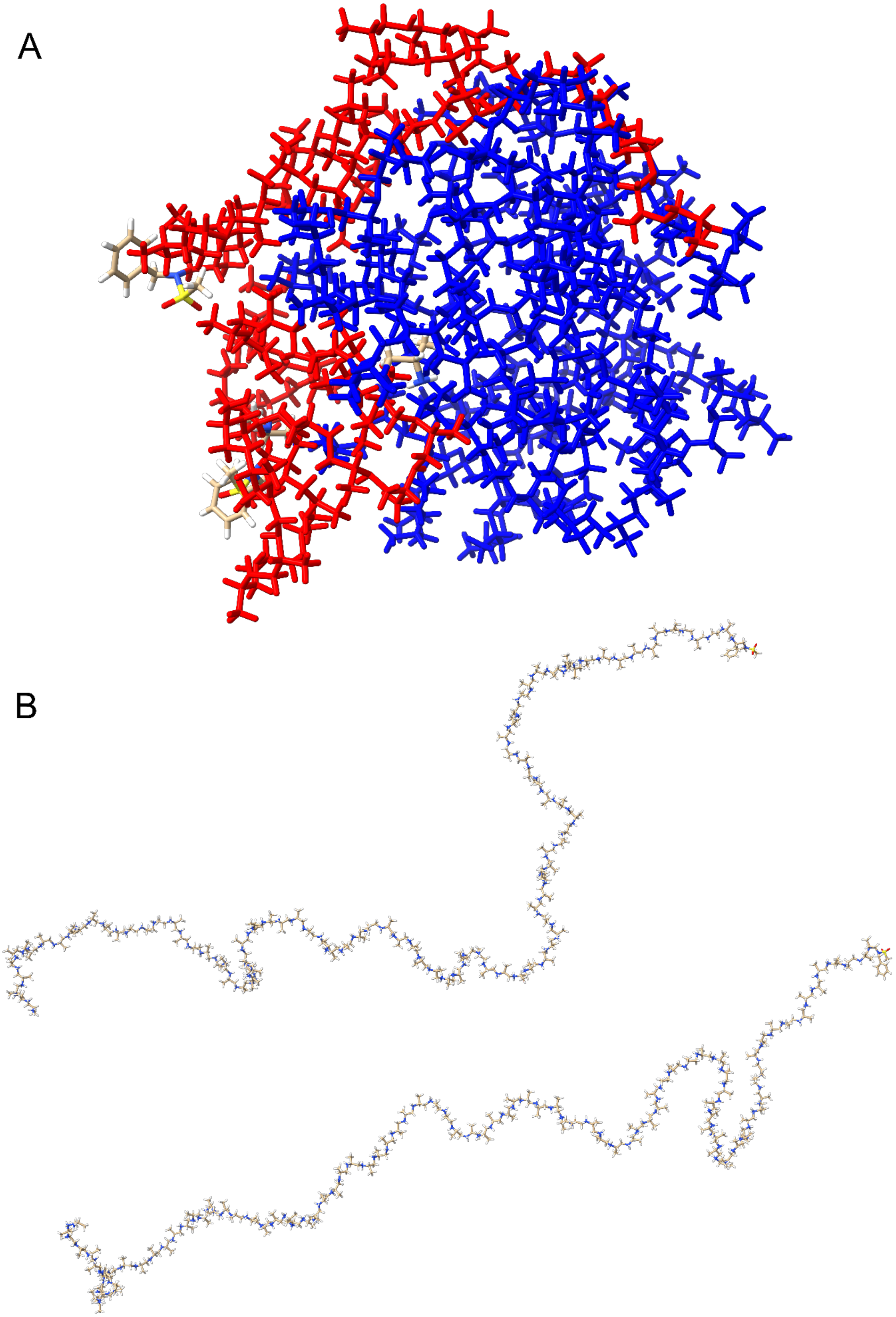

Figure S42. Snapshots of protonated and deprotonated duplexes by molecular dynamics. A: Non-protonated 30R-70S duplex, the R-units are marked as red and the S-ones as blue. B: Protonated 30R-70S duplex.

References

(1) Bannwarth, C.; Caldeweyher, E.; Ehlert, S.; Hansen, A.; Pracht, P.; Seibert, J.; Spicher, S.; Grimme, S. Extended <scp>tight-binding</Scp> Quantum Chemistry Methods. *WIREs Computational Molecular Science* **2021**, *11* (2). https://doi.org/10.1002/wcms.1493.

(2) Neese, F. Software Update: The <scp>ORCA</Scp> Program System—Version 5.0. *WIREs Computational Molecular Science* **2022**, *12* (5). https://doi.org/10.1002/wcms.1606.

(3) Bayly, C. I.; Cieplak, P.; Cornell, W.; Kollman, P. A. A Well-Behaved Electrostatic Potential Based Method Using Charge Restraints for Deriving Atomic Charges: The RESP Model. *J Phys Chem* **1993**, *97* (40), 10269–10280. https://doi.org/10.1021/j100142a004.

(4) *Red Server*. https://upjv.q4md-forcefieldtools.org/REDServer/ (accessed 2023-10-12).

(5) D.A. Case, H.M. Aktulga, K. Belfon, I.Y. Ben-Shalom, J.T. Berryman, S.R. Brozell, D.S. Cerutti, T.E. Cheatham, III, G.A. Cisneros, V.W.D. Cruzeiro, T.A. Darden, N. Forouzesh, G. Giambaşu, T. Giese, M.K. Gilson, H. Gohlke, A.W. Goetz, J. Harris, S. Izadi, S.A. Izmailov, K. Kasavajhala, M.C. Kaymak, E. King, A. Kovalenko, T. Kurtzman, T.S. Lee, P. Li, C. Lin, J. Liu, T. Luchko, R. Luo, M. Machado, V. Man, M. Manathunga, K.M. Merz, Y. Miao, O. Mikhailovskii, G. Monard, H. Nguyen, K.A. O'Hearn, A. Onufriev, F. Pan, S. Pantano, R. Qi, A. Rahnamoun, D.R. Roe, A. Roitberg, C. Sagui, S. Schott-Verdugo, A. Shajan, J. Shen, C.L. Simmerling, N.R. Skrynnikov, J. Smith, J. Swails, R.C. Walker, J. Wang, J. Wang, H. Wei, X. Wu, Y. Wu, Y. Xiong, Y. Xue, D.M. York, S. Zhao, Q. Zhu, and P.A. Kollman, Amber 2023. University of California: San Francisco 2023.

(6) He, X.; Man, V. H.; Yang, W.; Lee, T.-S.; Wang, J. A Fast and High-Quality Charge Model for the next Generation General AMBER Force Field. *J Chem Phys* **2020**, *153* (11). https://doi.org/10.1063/5.0019056.

(7) Wang, J.; Wolf, R. M.; Caldwell, J. W.; Kollman, P. A.; Case, D. A. Development and Testing of a General Amber Force Field. *J Comput Chem* **2004**, *25* (9), 1157–1174. https://doi.org/10.1002/jcc.20035.

(8) Johnson, D. C.; Widlanski, T. S. Cerium(III) Chloride-Mediated Reactions of Sulfonamide Dianions. *J Org Chem* **2003**, *68* (13), 5300–5309. https://doi.org/10.1021/jo034001w.

(9) Bieber, L. W.; De Araújo, M. C. F. Short and Efficient Synthesis of Optically Active N-Tosyl Aziridines from 2-Amino Alcohols. *Molecules* **2002**, *7* (12), 902–906. https://doi.org/10.3390/71200902.

(10) Splitstone, R. *Chiral Compounds – What to Know & How They Relate to Chromatography*. Phenomenex. https://phenomenex.blog/2020/06/11/chiral-compounds/.

(11) Kelly, S. M.; Jess, T. J.; Price, N. C. How to Study Proteins by Circular Dichroism. *Biochimica et Biophysica Acta (BBA) - Proteins and Proteomics* **2005**, *1751* (2), 119–139. https://doi.org/10.1016/j.bbapap.2005.06.005.
